# Folding‐Induced Promotion of Proton‐Coupled Electron Transfers via Proximal Base for Light‐Driven Water Oxidation

**DOI:** 10.1002/anie.202217745

**Published:** 2023-01-12

**Authors:** Niklas Noll, Tobias Groß, Kazutaka Shoyama, Florian Beuerle, Frank Würthner

**Affiliations:** ^1^ Institut für Organische Chemie Universität Würzburg Am Hubland 97074 Würzburg Germany; ^2^ Center for Nanosystems Chemistry (CNC) Universität Würzburg Theodor-Boveri-Weg 97074 Würzburg Germany; ^3^ Institut für Organische Chemie Universität Tübingen Auf der Morgenstelle 18 72076 Tübingen Germany

**Keywords:** Artificial Photosynthesis, Folded Macrocyles, Homogeneous Catalysis, Photocatalysis, Ruthenium Complexes

## Abstract

Proton‐coupled electron‐transfer (PCET) processes play a key role in biocatalytic energy conversion and storage, for example, photosynthesis or nitrogen fixation. Here, we report a series of bipyridine‐containing di‐ to tetranuclear Ru(bda) macrocycles **2 C**–**4 C** (bda: 2,2′‐bipyridine‐6,6′‐dicarboxylate) to promote O−O bond formation. In photocatalytic water oxidation under neutral conditions, all complexes **2 C**–**4 C** prevail in a folded conformation that support the water nucleophilic attack (WNA) pathway with remarkable turnover frequencies of up to 15.5 s^−1^ per Ru unit respectively. Single‐crystal X‐ray analysis revealed an increased tendency for intramolecular π‐π stacking and preorganization of the proximal bases close to the active centers for the larger macrocycles. H/D kinetic isotope effect studies and electrochemical data demonstrate the key role of the proximal bipyridines as proton acceptors in lowering the activation barrier for the crucial nucleophilic attack of H_2_O in the WNA mechanism.

## Graphical abstract

Structurally flexible, bipyridine‐containing Ru macrocyles have shown a remarkable increase in turnover per Ru center in visible‐light‐driven water oxidation catalysis. Conformational studies in the solid state revealed an increased tendency of the auxiliary base close to the active centers of the larger complexes to undergo intramolecular folding and preorganization, which facilitates proton abstraction in the water nucleophilic attack (WNA) pathway.

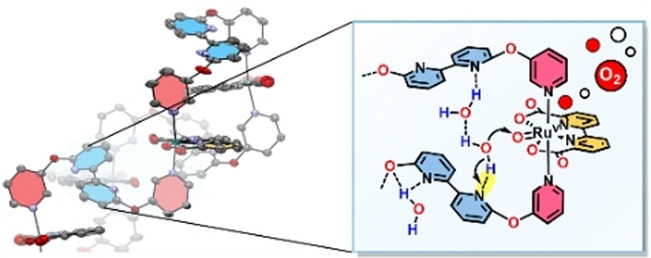

## Introduction

The molecular and biological properties of proteins are generally controlled by their three‐dimensional shape and conformation.[^1^, ^2^] To facilitate the high structural order of specific protein domains, sophisticated biological processes such as protein folding benefit from secondary, noncovalent interactions, e.g., hydrogen bonding, electrostatic interactions or hydrophobic effects.^[3]^ The folded, native structure is essential for the protein functionality, e.g., specific substrate recognition, as misfolding can lead to inactive or even toxic assemblies.^[4]^ Two prominent biological systems showcasing a highly sophisticated folded protein environment are the oxygen‐evolving complex of photosystem II (OEC‐PSII),[^5^, ^6^, ^7^] Nature's photosynthetic workhorse to accomplish the fourfold process of water oxidation, and the cytrochrome c oxidases, which catalyze the reverse reaction.[^8^, ^9^] Inspired by these natural archetypes, related principles have been applied in the development of artificial metal complexes to promote ligand‐substrate interactions via supramolecular approaches.[^10^, ^11^, ^12^, ^13^] For instance, reminiscent of natural [FeFe] hydrogenases, the incorporation of pendant amines into the ligand framework promoted intramolecular hydride transfer to accelerate proton reduction catalysis.[^14^, ^15^] Similar concepts have also been applied in ruthenium‐based water oxidation catalysis with several reports on the incorporation of proximal functionalities such as phosphonate or carboxylate groups. Under oxidative conditions, the auxiliary base acts as proton‐accepting unit to deprotonate incoming H_2_O molecules and, thus, significantly reduces the activation barrier for the water nucleophilic attack (WNA) pathway by facilitating PCETs.[^16^, ^17^, ^18^, ^19^] In recent years, our group has likewise demonstrated the enormous potential of implementing Ru(bda) (bda: 2,2′‐bipyridine‐6,6′‐dicarboxylate) catalysts into cyclic metallosupramolecular architectures, which led to a significant increase in both catalyst stability and performance via promotion of the WNA mechanism through cooperative effects between the catalytic centers.[^20^, ^21^, ^22^] Very recently, we have presented a high‐performing water oxidation catalyst (WOC) by the incorporation of a single Ru(bda) subunit into a well‐defined macrocyclic nanostructure equipped with a bipyridine‐functionalized ligand.^[23]^ Under acidic conditions, an enzyme‐mimetic molecular cleft is formed. In this catalytic pocket, a well‐defined water network is stabilized by hydrogen bonds to the protonated bipy (bipyridine) site, which cannot facilitate proton abstraction but rather serves as hydrogen bond acceptor for H_2_O preorganization. Under neutral conditions however, deprotonation of the bipyridinium site induces outward rotation of the free base, which breaks the catalytic pocket and significantly lowers the catalytic activity. Inspired by these exciting findings, we envisioned that the combination of both a preorganized H_2_O network and a proximal Brønsted base might further boost the catalytic performance of enzyme‐mimicking WOCs. Building on previously studied rigid macrocycles,[^20^, ^21^, ^22^] we expected that for more flexible, oxygen‐bridged macrocyclic frameworks of varying size, the interaction with and activation of the intracavity H_2_O environment can be optimized by tailored folding of the dynamic backbone. In particular, catalysis at pH 7 was envisaged to enable proton abstraction by pendant bipy units. Until now, synthetic foldamers^[24]^ have found broad application in the fields of nanotechnology or biomedicine[^25^, ^26^] while their catalytic functions have only been reported for selected examples focusing on C−C bond formation and cleavage reactions[^27^, ^28^, ^29^] but not for water oxidation catalysis.

Here, we introduce a novel series of structurally more flexible bipy‐functionalized di‐ to tetranuclear Ru(bda) catalysts **2 C**–**4 C**. Our detailed studies revealed a tremendous increase in catalytic performance under neutral photocatalytic conditions with turnover frequencies (TOFs) of 5.5 s^−1^, 14 s^−1^ and 15.5 s^−1^ per Ru unit for complexes **2 C**–**4 C**, respectively, which even outperform the previously reported benchmark macrocycle **MC3** with a TOF_Ru_ of 3.7 s^−1^.[^20^, ^22^] Conformational insights by single‐crystal X‐ray analysis for the whole series showcased how the tendency for intramolecular folding, driven by π‐π stacking of the bipy units, increased from **2 C** to **4 C** and orientated the proton‐accepting groups towards the active centers. This folding‐induced preorganization of both reactive Ru centers and pendant bipy bases in the larger macrocycles increases the catalytic performance from dimer **2 C** to tetramer **4 C** by facilitating PCETs in the WNA pathway, which was experimentally confirmed by H/D kinetic isotope effect (KIE) studies and electrochemical measurements.

## Results and Discussion

**Scheme 1:**
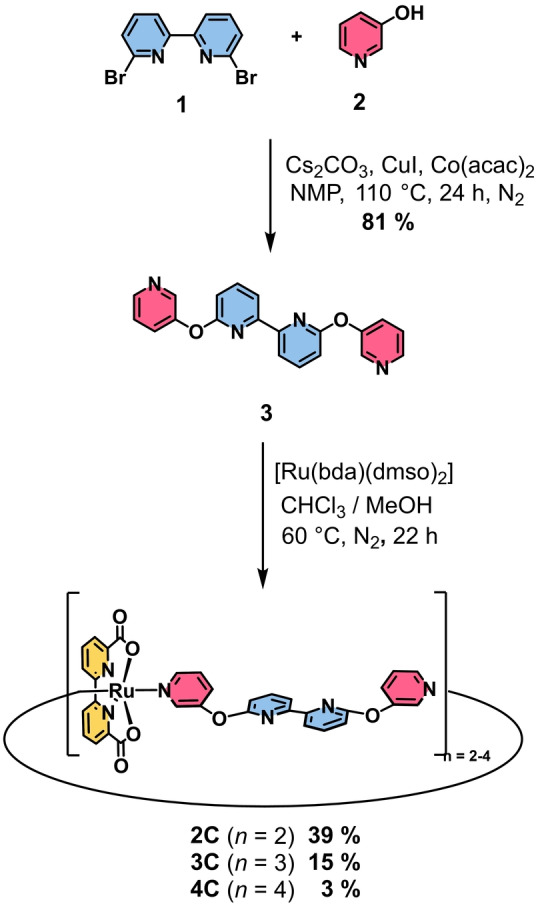
Two‐step procedure for the synthesis of multinuclear Ru(bda) complexes **2 C**–**4 C**.

Multinuclear Ru(bda) macrocycles **2 C**–**4 C** were synthesized in a two‐step procedure (Scheme 1). First, *m*‐hydroxypyridine (**2**) was attached to the bipy linker **1** via twofold nucleophilic aromatic substitution reaction to give ditopic ligand **3** with modestly flexible diaryl ether linkages. Subsequently, a mixture of multinuclear complexes **2 C**–**4 C** were synthesized via twofold ligand exchange reaction at the Ru precursor [Ru(bda)(dmso)_2_]^[30]^ with bidentate ligand **3**. The separation of the macrocycles of different size was achieved by size‐exclusion chromatography to yield pure dimer **2 C** and trimer **3 C** in 39 % and 15 % yield, respectively. Further purification of crude tetramer **4 C** by gel permeation chromatography (GPC) to remove open‐ and closed‐chain side products of higher nuclearity afforded pure **4 C** in 3 % yield. Detailed synthetic procedures and characterization data for all new compounds are provided in the Supporting Information.

**Figure 1:**
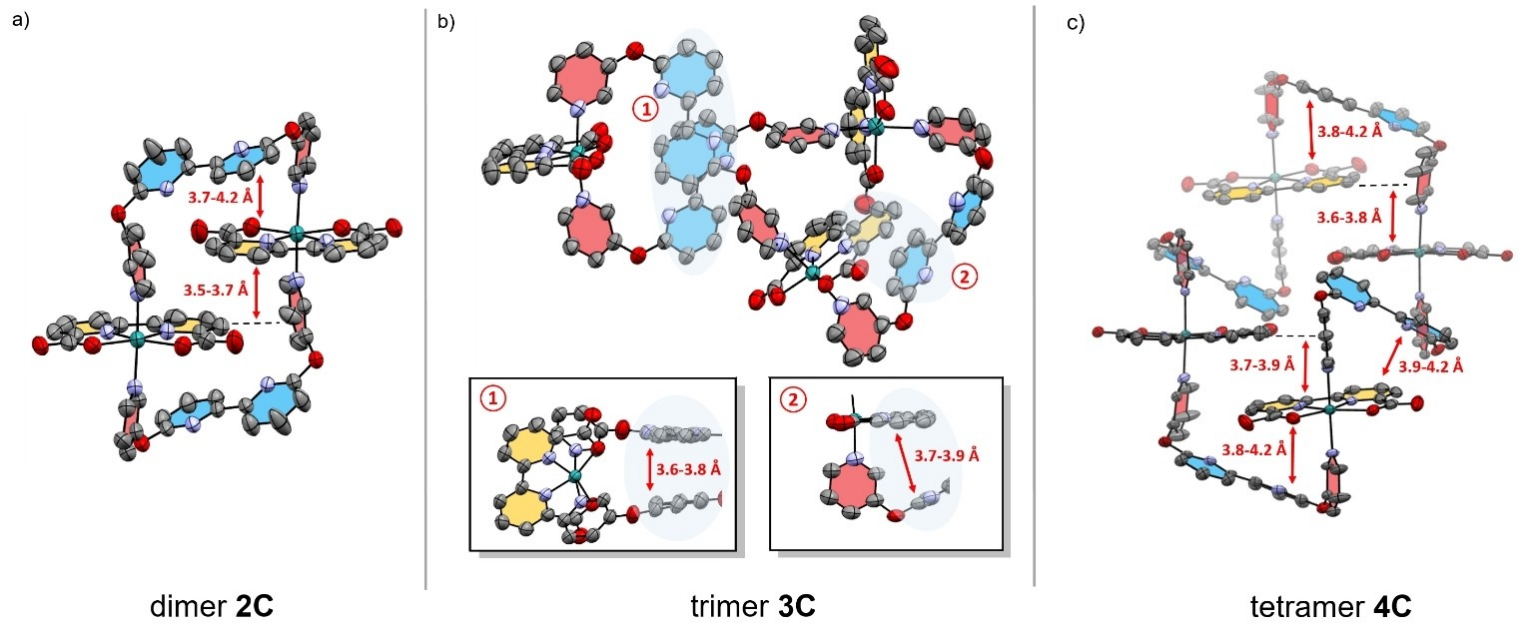
a)–c) Single‐crystal X‐ray structures for **2 C**–**4 C** under neutral conditions. The respective distances between intramolecular π‐π interactions are highlighted. For **2 C** only one of the two macrocyclic complexes in the unit cell is shown; organic solvent molecules and hydrogen atoms of the respective complexes are omitted for clarity. ORTEP diagram with thermal ellipsoids set at 50 % probability; C, grey; O, red; N, purple; Ru, turquoise.

Single crystals suitable for X‐ray diffraction of all complexes **2 C**–**4 C** in the initial Ru^II^ state were grown either by slow evaporation or vapour diffusion (for crystallographic details see Supporting Information). The three solid‐state structures shown in Figure 1 unequivocally confirm the cyclic nature of the multinuclear complexes. Solid‐state packing and macrocycle conformations are strongly affected by the *trans*‐oriented bipy units in the axial ligand backbone. All complexes exhibit distorted octahedrally coordinated Ru centers with obtuse O−Ru−O angles in the range of 121–123° (Table S1), which is in good accordance with the previously reported macrocyclic Ru complex **MC4** (123.0(2)°) from our group.^[21]^ The conformation of dimer **2 C** is dominated by intramolecular π‐π interactions (*d*=3.7–4.2 Å),^[31]^ which induce a rigid parallel stacking of the two reversely oriented Ru(bda) units between the two bipy moieties (Figure 1a). After incorporation of another molecular unit in trimer **3 C**, the closely folded structure opens up into a less symmetric conformation (Figure 1b). π‐π*‐*Stacking (*d*=3.6–3.8 Å) between two of the three bipy units in trimer **3 C** results in three crystallographically distinct Ru sites, with one Ru(bda) unit being directly aligned towards the π‐π‐interacting bipy units (cutout 1 in Figure 1b). For the largest macrocycle **4 C**, a more ordered conformation is again observed that is dictated by π‐π‐interactions between the axial and equatorial ligand spheres (Figure 1c). This arrangement leads to similar distances between the four reversely oriented Ru(bda) units and positions the basic bipy units near the reactive Ru sites. In summary, these solid‐state X‐ray structures demonstrate the ordering effect of intramolecular π‐π‐interactions within these series of semi‐rigid macrocycles. Whereas a rather parallel stacking between the bipy and bda units rigidifies the structure of dimer **2 C**, the incorporation of additional molecular units within the larger and more flexible complexes **3 C** and **4 C** allows for preorganization of the proximal base near the active centers.

**Figure 2:**
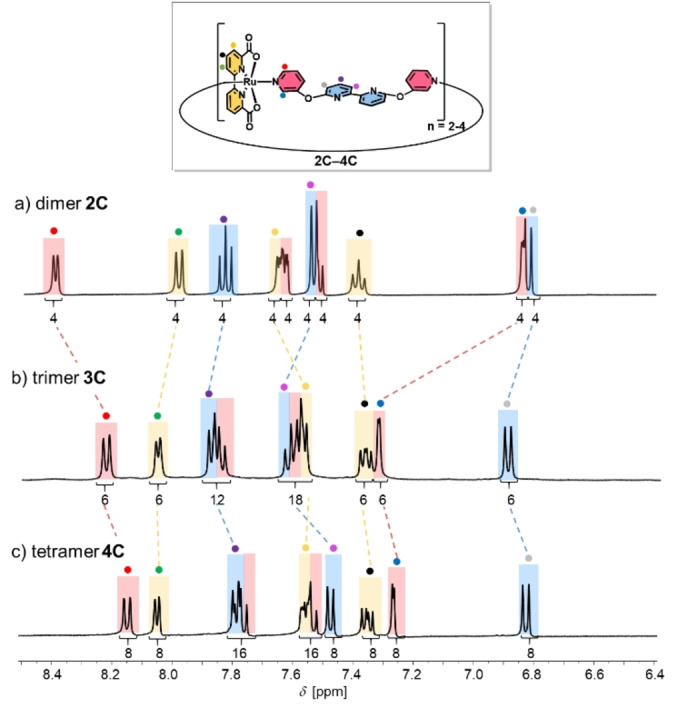
a)–c) Aromatic region of the ^1^H NMR spectra (1 : 1 TFE‐*d_3_*/D_2_O, 400 MHz, ascorbic acid, rt) of complexes **2 C**–**4 C** at pD 7.0. The colors of the signals correspond to bda (yellow), axial pyridine fragment (red) and bipyridine unit (blue) of the ligand backbone as highlighted in the structure.

To determine whether these conformational nuances of **2 C**–**4 C** in the solid state are maintained in aqueous solution, we measured ^1^H NMR spectra of the three complexes in aqueous mixtures of 1 : 1 D_2_O/TFE‐*d*_3_ (pD=7.0) (Figure 2). All proton signals were assigned based on 2D NMR spectroscopy (Figures S8–S10). For all three macrocycles, only one set of signals was observed for all chemically non‐equivalent protons. This indicates either a highly symmetrical structure or dynamic relaxation between more folded conformers that is fast on the NMR time scale. With increasing size of the cyclic complexes, distinct chemical shift changes are observed especially for the *ortho* protons of the axial pyridine units. While the red‐labelled *ortho* protons have a moderate upfield shift when going from dimer **2 C** (8.41 ppm) to trimer **3 C** (8.23 ppm), the reverse effect is observed for the blue‐labelled opposite *ortho* protons (6.85 ppm (**2 C**); 7.33 ppm (**3 C**); Figures 2a,b). These opposing chemical shift changes were attributed to a partially restricted rotation of the Ru(bda) units, which exposes the red‐ and blue‐labelled protons either to enhanced magnetic shielding from the adjacent equatorial bda ligands (upfield shift)^[32]^ or to the open Ru site (downfield shift). Further evidence for this conformational rotation in trimer **3 C** is given by nuclear Overhauser effect (NOE) cross signals between the bda unit (green proton) and both red‐ and blue‐labelled protons (Figure S9), which indicates a rather flexible character of the axial pyridine units and, thus, every Ru unit can rotate more freely. By contrast, only one NOE cross signal was observed for **2 C** between the green‐labelled proton of the bda unit and the blue‐labelled *ortho* proton (Figure S8), which suggest a much more rigid conformation for the smallest macrocycle. For the larger **4 C**, very similar ^1^H NMR data with only minor upfield shifts compared to **3 C** and equal NOE cross signals between the bda unit and both red‐ and blue‐labelled *ortho* protons were obtained (Figure 2c and Figure S10). Since only three sharp signals without any splitting at room temperature were observed for the bda units in the larger complexes **3 C** and **4 C**, fast switching of these axial ligands between different conformers on the NMR time scale is suggested. To check the possibility for freezing out the predominant conformers and to get deeper insight into structural peculiarities, VT‐NMR experiments were performed for both **3 C** and **4 C** from 190–295 K in CD_2_Cl_2_/CD_3_OD 1 : 1 (Figures S11 and S12). Unfortunately, no defined signal splitting due to symmetry breaking but only severe signal broadening was observed in the accessible temperature range. These results suggest still highly dynamic conformations and, thus, did not allow any structural assignment at lower temperatures. For dimer **2 C**, similar experiments could not be performed due to the poor solubility of the complex in organic media. To probe for the effect of protonation of the bipy units,^[23]^ we compared ^1^H NMR spectra for **2 C**–**4 C** at pD 7.0 and 1.0 (1 : 1 TFE‐*d*_3_/D_2_O, 0.1 M CF_3_SO_3_D) (Figures S13–S15 and Tables S3–S5). For all three macrocyles, the respective blue‐labelled protons show a moderate downfield shift at pD 1.0, whereas the signals of the red‐labelled protons only changed negligible for the larger complexes **3 C** and **4 C**. In addition, most of the signals for the respective axial bipy units showed minor downfield shifts under acidic conditions. These results indicate a more dynamic rotation of the Ru(bda) units due to protonation of the bipy units under acidic conditions.[^23^, ^33^] Due to repulsive interactions between the charged bipyridinium units, the folded structures open up, which diminishes any close intramolecular interactions between the axial and equatorial ligand sphere. This is also evidenced by the absence of NOE cross signals between the green‐labelled protons of the bda units and the purple‐labelled protons protons of the axial bipy units (Figures S16–S18). More proof for this lack of stabilizing intramolecular folding in the protonated macrocycles is given by moderate upfield shifts for the green‐labelled protons of the bda units in the larger complexes **3 C** and **4 C** (Figures S14, S15 and Tables S4, S5).

To probe the effect of pH change on size and conformation of **2 C**–**4 C** in solution, diffusion‐ordered spectroscopy (DOSY) measurements were performed in aqueous mixtures of D_2_O/TFE‐*d*_3_ 1 : 1 at pD 1.0 (0.1 M CF_3_SO_3_D) or pD 7.0 (Figures S19–S24). Notably, varying diffusion coefficients for **2 C**–**4 C** were obtained by DOSY NMR and the hydrodynamic radii were calculated via the Stokes–Einstein equation. Under neutral conditions, a continuous increase of the hydrodynamic radii was observed with increasing complex size for **2 C**–**4 C** (*r*_H_(**2 C**)=11.6 Å; *r*_H_(**3 C**)=11.9 Å; *r*_H_(**4 C**)=15.6 Å) (Figures S20, S22, S24). The obtained values are in good accordance with space‐filling models obtained from the solid‐state structures of **2 C**–**4 C**. Under acidic conditions, only slightly larger hydrodynamic radii were observed for **2 C** and **3 C**, while a larger difference of 1.1 Å between the hydrodynamic radii at pH 1 and pH 7 was observed for tetranuclear complex **4 C** (d(*r*_H_(pH 1, **2 C**)–*r*_H_(pH 7, **2 C**)=0.2 Å; d(*r*_H_(pH 1, **3 C**)–*r*_H_(pH 7, **3 C**)=0.1 Å; d(*r*_H_(pH 1, **4 C**)–*r*_H_(pH 7, **4 C**)=1.1 Å) (Figures S19, S21, S23). In summary, these results provide further evidence for the strong intramolecular folding of complexes **2 C**–**4 C** under neutral conditions, while a continuous trend towards larger unfolded complex sizes was observed under acidic conditions.

**Figure 3:**
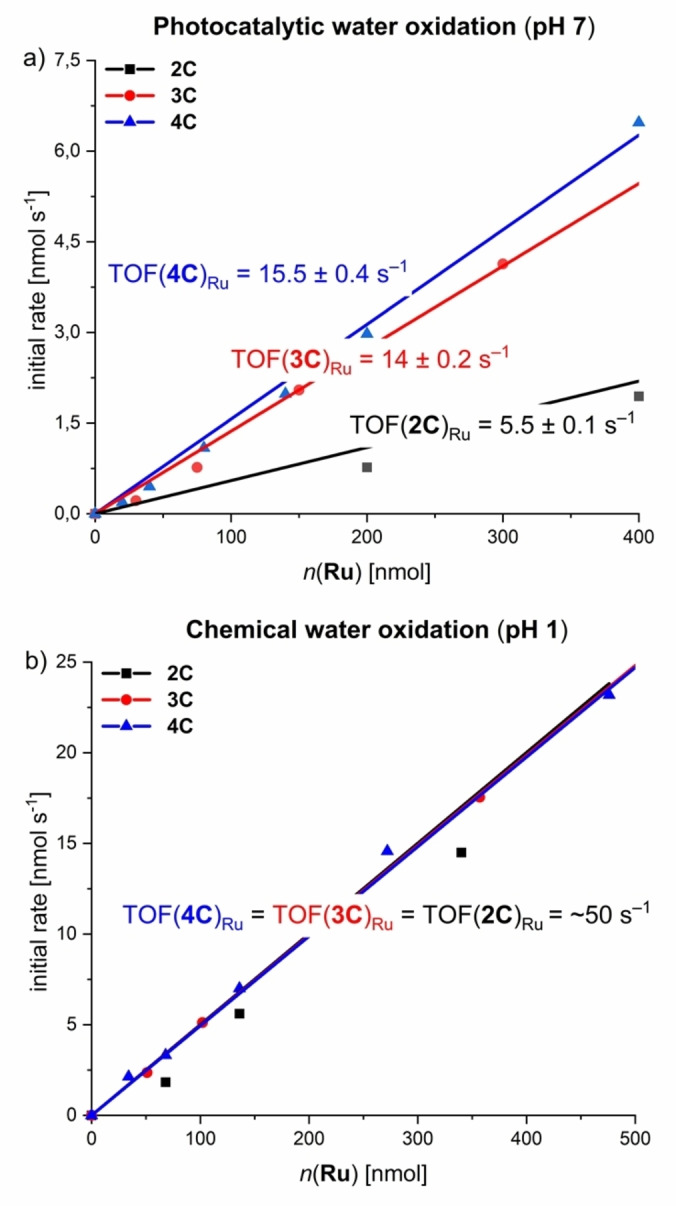
a) Photochemical water oxidation catalysis with **2 C**–**4 C**: Plot of initial rates (obtained by linear fit of O_2_ evolution curve after light exposure between 50–60 s) of O_2_ evolution against the WOC concentration and linear fit for first order kinetics. O_2_ evolution experiments were performed at varying the WOC concentrations in CH_3_CN/H_2_O 4 : 6 (pH 7, 50 mM phosphate buffer, *c*(PS)=1.5 mM, *c*(Na_2_S_2_O_8_)=37 mM). b) Chemical water oxidation experiments with **2 C**–**4 C** as WOCs in 4 : 6 CH_3_CN/H_2_O mixture using CAN as a sacrificial oxidant (pH 1, triflic acid, *c*(CAN)=0.6 M). Plots of initial rates of O_2_ evolution against the WOC concentration with corresponding linear regression fit. Individual reaction rates were obtained by a linear fit of O_2_ evolution curves for the first 2 s of catalysis for **2 C**–**4 C**, respectively.

Towards artificial photosynthesis, the catalytic properties of complexes **2 C**–**4 C** were investigated under photochemical conditions in a three‐component system in 50 mM phosphate buffered CH_3_CN/H_2_O 4 : 6 mixtures at pH 7 with [Ru(bpy)_3_Cl_2_] as photosensitizer and Na_2_S_2_O_8_ as sacrificial electron acceptor (Figures 3a and S25; for detailed experimental conditions see Supporting Information).[^34^, ^35^, ^36^, ^37^, ^38^, ^39^, ^40^, ^41^] Irradiation was performed with a xenon lamp (*I*=100 mW cm^−1^) and a Clark electrode was used for O_2_ detection. All complexes displayed a first‐order dependency of the evolution of O_2_ on the WOC concentration, which is typical for catalysts following the WNA pathway as previously shown for multinuclear macrocycles from our group.[^20^, ^21^, ^22^, ^42^] As shown in Figure 3a, the catalytic activity of **2 C**–**4 C** strongly correlates with the size of the macrocycle. Apparently, the activity per Ru unit is almost three times higher for trimer **3 C** (TOF_Ru_(**3 C**)=14±0.2 s^−1^, Figure S28) compared to dinuclear counterpart **2 C** (TOF_Ru_(**2 C**)=5.5±0.1 s^−1^, Figure S27). For the largest macrocyle **4 C**, only a moderate increase in catalytic performance (TOF_Ru_(**4 C**)=15.5±0.4 s^−1^, Figure S28) was observed. These activities also correlate with the turnover numbers (TON) per Ru center, as the larger macrocycles **3 C** and **4 C** (TON(**3 C**)_Ru_=550±50; TON(**4 C**)_Ru_=600±50) exhibit significantly higher turnover compared to dimer **2 C** (TON_Ru_(**2 C**)=200±20). The catalytic performance of these macrocyclic Rub(bda) complexes is among the most active homogeneous WOCs reported to date (see table S6 for a comparison with recent literature examples). While the smallest macrocyle **2 C** has already a catalytic activity comparable to our previously reported tri‐ and tetranuclear macrocycles **MC3** (TOF_Ru_=3.7 s^−1^)[^20^, ^22^] and **OEG‐MC4** (TOF_Ru_=5.8 s^−1^),^[42]^ the larger analogs significantly outperform most of the literature benchmarks.

To probe if this trend in activity is maintained after protonation of the bipy units, all three Ru(bda) complexes were investigated in chemical water oxidation under acidic conditions (CH_3_CN/H_2_O 4 : 6, pH 1, triflic acid) using cerium ammonium nitrate as sacrificial oxidant^[34]^ (for experimental details see Supporting Information). Surprisingly, a similar activity for each Ru center with a TOF/Ru of ≈50 s^−1^ was observed for the whole series **2 C**–**4 C** via linear regression of the first‐order kinetics of O_2_ evolution (Figure 3b and Figures S30–S32). This activity is even comparable to our previously reported macrocycle **MC3** with a TOF_Ru_ of 45 s^−1^ under identical conditions.[^20^, ^22^] The same trend is reflected in the TON of the respective complexes, which gave very similar values for the whole series (TON(**2 C**)_Ru_=500±100; TON(**3 C**)_Ru_=500±100;TON(**4 C**)_Ru_=450±100). Analysis of the reaction mixtures of **2 C**–**4 C** by MALDI‐TOF mass spectrometry revealed high stabilities of the complexes after catalysis, as indicated by identical fragmentation patterns before and after catalysis, which was attributed to decomposition of the complexes by ionization during mass spectrometry (Figures S34–S42). Accordingly, the macrocyclic nature of **2 C**–**4 C** confers higher stability through the chelate effect of the ditopic ligand **3**.[^20^, ^21^] For further insight into the mechanistic pathway, kinetic isotope effect (KIE) studies were performed for **2 C**–**4 C** under previously described photocatalytic conditions in 50 mM phosphate buffered aqueous solutions (H_2_O and D_2_O, pH 7) containing 40 % acetonitrile.^[43]^ For all three multinuclear complexes, linear kinetics were observed with a significantly higher reaction rate in H_2_O compared to D_2_O. The highest KIEs of 1.9 and 2.4 were observed for tri‐ and tetranuclear complexes **3 C** and **4 C**, respectively. In contrast, dinuclear complex **2 C** exhibits a significantly lower KIE of 1.4 (Figures S43–S45). Therefore, a competing intramolecular I2M pathway under these conditions cannot completely be ruled out for the compact conformation of rigid **2 C**. In accordance with previous results,^[21]^ these findings indicate a stronger degree of proton coupling in the rate‐determining Ru^IV^−OH to Ru^V^=O oxidation within the mechanistic pathway of the larger macrocycles **3 C** and **4 C** at pH 7. Under these conditions, the proximal bipy groups serve as proton acceptor in close proximity to the reactive Ru sites compared to its protonated state at pH 1.

**Figure 4:**
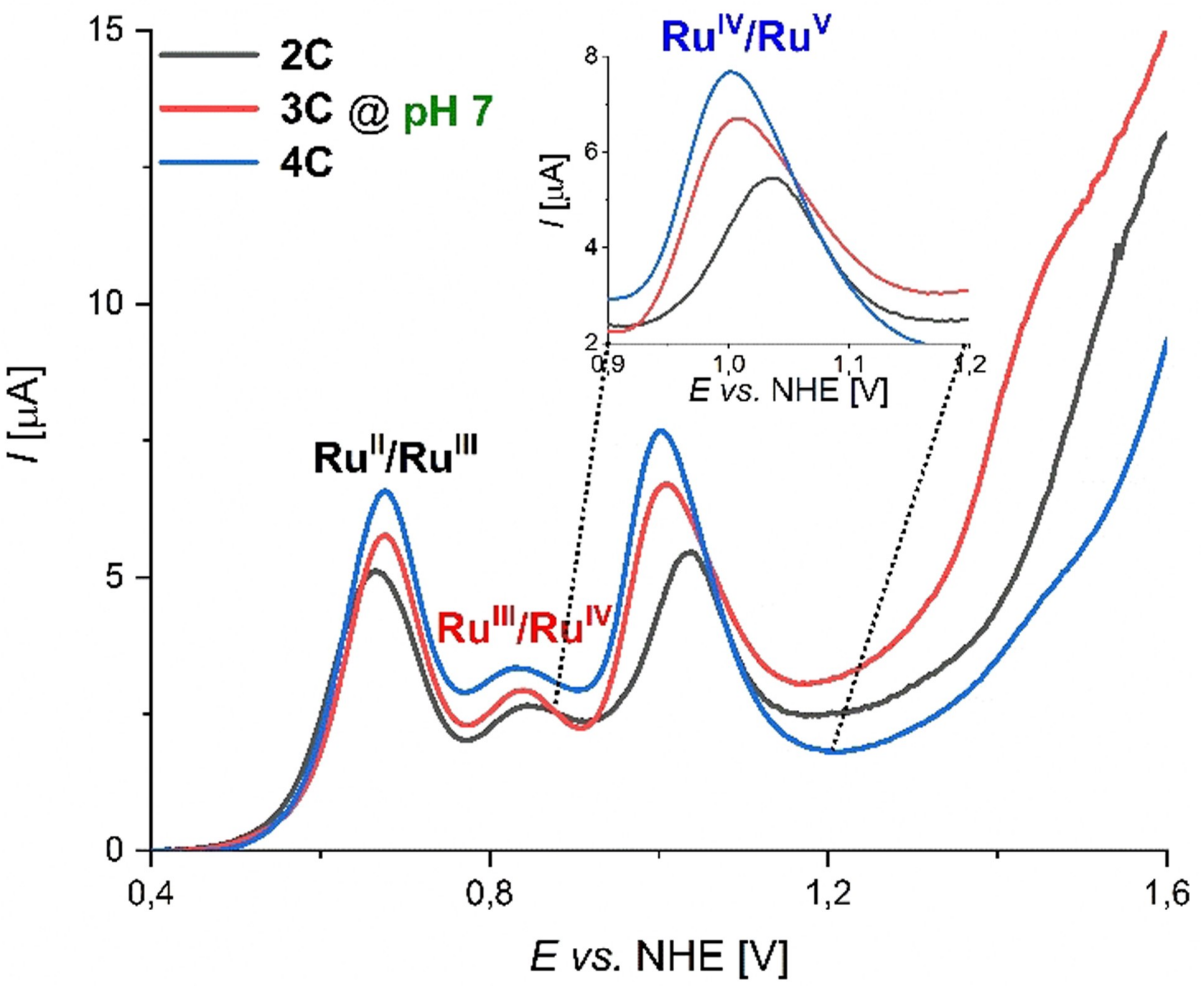
Differential pulse voltammograms of macrocycles **2 C**–**4 C** at pH 7 in TFE/H_2_O 4 : 6 (phosphate buffer with *I*=0.1 M, *c*(**WOC**)=2.5×10^−4^ M). The inset shows the amplified region between 0.9–1.2 V at pH 7.

Comparative studies on photocatalytic (pH 7) and chemical (pH 1) water oxidation for a series of base‐functionalized di‐ to tetranuclear Ru(bda) macrocyles **2 C**–**4 C** revealed a significant increase in activity induced by basic bipy moieties in the semi‐flexible ligand framework. Under photocatalytic conditions, an unprecedented boost in catalytic performance was observed with TOF_Ru_ values of 5.5 s^−1^, 14 s^−1^, and 15.5 s^−1^ for **2 C**–**4 C**, respectively. Under chemical conditions however, each Ru unit of macrocycles **2 C**–**4 C** with varying size exhibited an average TOF of ≈50 s^−1^ with no apparent size effects. In comparison to our previously reported trinuclear macrocycle **MC3**, very similar activities are obtained under acidic conditions (TOF_Ru_ (**MC3**)=45 s^−1^).[^20^, ^22^] At pH 7 however, the base‐containing complexes **2 C**–**4 C** clearly outperform **MC3** (TOF_Ru_(**MC3**)=3.7 s^−1^). Interestingly, H/D KIE studies under neutral conditions revealed a direct correlation between the catalytic activity and the degree of proton coupling in the rate‐determining nucleophilic attack of an H_2_O molecule in the WNA pathway. Additional support for these findings was obtained by cyclic voltammetry (CV) and differential pulse voltammetry (DPV). Measurements for **2 C**–**4 C** were performed in phosphate‐buffered aqueous solutions at pH 7 containing 40 % 2,2,2‐ trifluorethanol (TFE) as a non‐coordinating co‐solvent for better solubilization and the redox properties are summarized in Table S7. Under neutral conditions, three subsequent oxidation processes were observed for all three compounds **2 C**–**4 C**, which can be assigned to the Ru^II^/Ru^III^, Ru^III^/Ru^IV^ and Ru^IV^/Ru^V^ redox couples, respectively (Figures S46–S48). At pH 7, the typically rate‐determining Ru^IV^/Ru^V^ oxidations for **3 C** and **4 C** are cathodically shifted by 30–40 mV with regard to dimer **2 C** and a gradual enhancement of the catalytic current density is observed (Figure 4). This thermodynamically more favourable oxidation to Ru^V^ for the larger macrocycles is presumably induced by a stronger contribution of the proximal proton acceptor unit in the rate‐determining step of O−O bond formation for the larger complexes, as it was recently shown for a Ru‐based catalyst.^[44]^ In addition, Pourbaix diagrams for **2 C** and **3 C** display the involvement of proton‐coupled electron transfer (PCET) processes for the Ru^III^/Ru^IV^ and Ru^IV^/Ru^V^ redox couples, while the oxidation from Ru^II^ to Ru^III^ is independent of the pH value (Figure S49). For the Ru^III^/Ru^IV^ oxidation event, slopes in the range of −57 to −74 mV per pH unit are found for these complexes indicating 2 e^−^/2 H^+^ or 3 e^−^/3 H^+^ processes, respectively, in accordance with the Nernstian ideal of 59 mV/pH for a general *n* e^−^/*n* H^+^ process.^[45]^

**Figure 5:**
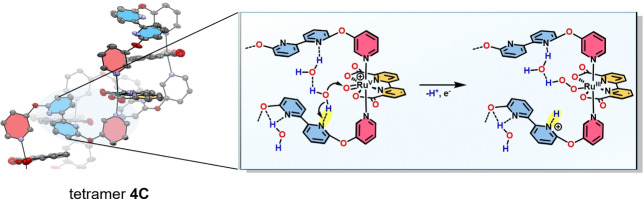
Cutout from the single‐crystal X‐ray structure of tetramer **4 C** showcasing the effect of proton abstraction of the pendant base (highlighted in yellow) within the rate‐determining step of O−O bond formation in the WNA pathway.

Based on the combined analytical and kinetic data, we propose the following mechanistic picture for the excellent water oxidation catalysis with **2 C**–**4 C**. As shown previously, a hydrogen‐bonded H_2_O network can either be stabilized by cooperative effects within the cavities of macrocyclic Ru(bda) assemblies of different size by utilizing the carboxy groups of the bda ligands as directional bonding sites or via hydrogen‐bonding interactions to a protonated bipy site located opposite to the active site within a synthetic molecular cleft.[^22^, ^23^, ^42^, ^46^] In this work, we demonstrate the powerful effect of an auxiliary base in the ligand sphere on the catalytic performance of more flexible macrocycles of varying size. Single‐crystal X‐ray analysis of the multinuclear series **2 C**–**4 C** showed that within the larger complexes **3 C** and **4 C**, intramolecular π‐π interactions of the *trans*‐oriented bipy units put the proximal base in close proximity to the active sites. Detailed ^1^H‐NMR experiments gave further support that these solid‐state conformations of **2 C**–**4 C** are to some extent preserved in solution. Therefore, we conclude that the free bipy moieties act as proton‐accepting units at pH 7 and facilitate PCETs by deprotonating incoming H_2_O molecules at the rds of O−O bond formation, which promotes the subsequent hydroperoxide formation (Figure 5). The observed boost in catalytic performance for the larger and more folded macrocycles can be presumably attributed to a stronger preorganization of the proton‐accepting unit near the active site, since the proton transfer efficiency strongly depends on the donor‐acceptor distance and the right structural orientation.[^16^, ^47^, ^48^, ^49^] At pH 1, the now *cis*‐oriented bipyridinium cations[^23^, ^33^] can no longer accept any protons and a constant TOF of ≈50 s^−1^ without any size effect is observed for the whole series **2 C**–**4 C**. Here, the non‐folded conformations of the macrocycles are reminiscent of **MC3**, with the protonated bases preorganizing several water molecules and presumably stabilizing a hydrogen‐bonding network.

## Conclusion

In this work, we have integrated a base‐functionalized ligand as a proton acceptor within the flexible architecture of a series of di‐ to tetranuclear Ru macrocyles **2 C**–**4 C**. Single‐crystal X‐ray analysis of complexes **2 C**–**4 C** revealed that the conformations of the differently sized complexes are dominated by intramolecular π‐π interactions from the bipy unit in the axial ligand sphere. For the larger tri‐ and tetranuclear macrocyles, this leads to an increased tendency of intramolecular folding and a more pronounced orientation of the active centers towards the auxiliary base. In photocatalytic water oxidation at pH 7, a remarkable increase in TOF per Ru center to 5.5 s^−1^, 14 s^−1^ and 15.5 s^−1^ was observed for **2 C**–**4 C**, respectively, while following the WNA pathway. Under chemical conditions at pH 1, however, the activity per Ru unit remained constant with a value of about 50 s^−1^ for the whole series. Our detailed kinetic studies and electrochemical measurements under neutral conditions revealed an increasing H/D kinetic isotope effect and a cathodic shift of the rate‐determining Ru^IV^/Ru^V^ oxidation in the series **2 C**–**4 C**. This is most likely explained by the more flexible proximal base facilitating proton abstraction with increased tendency of intramolecular folding in the rate‐determining Ru^IV^−OH to Ru^V^=O oxidation in the WNA pathway. Under acidic conditions, transformation of the bipy units to the respective *cis*‐conjugated acids opens up the folded structures and shuts down the proton relay function. In summary, this study highlights the importance of second coordination sphere engineering for efficient molecular water oxidation catalysis and showcases the importance of a proximal base as an auxiliary ligand to enhance the crucial substrate–catalyst interaction.

## Conflict of interest

The authors declare no conflict of interest.

1

## Supporting information

As a service to our authors and readers, this journal provides supporting information supplied by the authors. Such materials are peer reviewed and may be re‐organized for online delivery, but are not copy‐edited or typeset. Technical support issues arising from supporting information (other than missing files) should be addressed to the authors.

Supporting Information

Supporting Information

Supporting Information

Supporting Information

## Data Availability

The data that support the findings of this study are available from the corresponding author upon reasonable request.
