# Supplementary material for: Folding‐Induced Promotion of Proton‐Coupled Electron Transfers via Proximal Base for Light‐Driven Water Oxidation
Source: Angew Chem Int Ed Engl. 2023 Jan 12;62(7):e202217745. doi: 10.1002/anie.202217745 (PMC10107485; doi:10.1002/anie.202217745)
Supplement: Supplementary file 1 — Supporting Information [file ANIE-62-0-s003.pdf]

Angewandte Chemie
Eine Zeitschrift der Gesellschaft Deutscher Chemiker

Supporting Information

Folding-Induced Promotion of Proton-Coupled Electron Transfers via Proximal Base for Light-Driven Water Oxidation

*N. Noll, T. Groß, K. Shoyama, F. Beuerle, F. Würthner**

Table of Contents

Materials and methods S3

Experimental Procedures S5

UV/Vis absorption spectroscopy S6

Single crystal X-ray analysis S6

NMR experiments @ pD 7 .... S11

VT–NMR experiments S14

NMR experiments @ pD 1 .... S16

DOSY NMR experiments S22

Photocatalytic water oxidation S25

Chemical water oxidation S27

Stability tests before chemical water oxidation S32

Stability tests under conditions of chemical water oxidation S35

Kinetic Isotope Effect S41

Electrochemistry S43

NMR spectra S45

HR mass spectra S49

References S51

Materials and methods

General
All chemicals and solvents were purchased from commercial suppliers and used as received. 2,2'-Bipyridine-6,6'-dicarboxylic acid (**bda**),[S1] [RuCl$_2$(dmso)$_4$],[S2,S3] and [Ru(bda)(dmso)$_2$][S4,S5] were synthesized according to literature known procedures. Reactions with air or moisture sensitive reagents were carried out under a nitrogen atmosphere using standard Schlenk techniques. Crude isolated compounds were further purified by column chromatography on silica gel (60M, 0.04-0.063 mm, Macherey-Nagel) with freshly distilled solvents. For experiments under aqueous conditions, 50 mM phosphate buffer solutions at pH 7 were used. The respective solution was prepared by dissolving NaH_2PO_4 (3.71 g, 30.9 mmol) and Na_2HPO_4 (2.7 g, 19.1 mmol) in deionized water or deuterated water (1000 mL).

Chromatography
For analytic thin-layer chromatography (TLC), pre-coated sheets with silica gel 60 layer (0.20 mm) with fluorescent indicator UV_{254} (Alugram Xtra SIL G/UV_{254}, Macherey-Nagel) were used. Crude isolated compounds were purified by column chromatography on silica gel (60M, 0.04-0.063 mm, Macherey-Nagel) with freshly distilled solvents. For further purification, size exclusion chromatography with Bio-Beads particles (S-X1 Support (1% crosslinkage, 40–80 μM bead size, 600–14000 MW exclusion range) or SX-3 Support (3% crosslinkage, 40–80 μM bead size, ≤2000 MW limit), Bio-Rad) suspended in a 9:1 DCM/MeOH mixtures (HPLC grade) were used. Recycling gel permeation chromatography (GPC) was performed on a LC-5060 system of Japan Analytical Industries (three columns from Agilent, PLgel, 10 μm, 100 Å; 10 μm, 100 Å; 10 μm, 500 Å) using a mixture of 9:1 $CHCl_3$/MeOH (HPLC grade).

NMR spectroscopy
^1H NMR and proton decoupled ^{13}C NMR spectra were recorded on a Bruker Avance III HD 400 spectrometer at 298 K. Chemical shifts (δ) are reported in parts per million (ppm) relative to tetramethylsilane and referenced internally to the residual solvent signal.[S6] The respective coupling constants (J) are given in Hertz (Hz). To describe signal multiplicities, the following abbreviations were applied: s = singlet, d = doublet, t = triplet and m = multiplet. In addition, 2D NMR spectra (COSY, NOESY, HSQC and HMBC) were recorded to allow a correct assignment of 1D NMR spectra of novel compounds.

DOSY NMR
DOSY (Diffusion-ordered spectroscopy) spectra were carried out in aqueous mixtures of 1:1 D_2O/TFE-d_3 (pD = 1.0 (0.1 M CF_3SO_3D) or pD = 7.0) on a Bruker Avance III HD 600 spectrometer at 295 K. The hydrodynamic radius of the differently sized complexes **2C**—**4C** were calculated assuming a spherical shape in solution according to the Stokes-Einstein equation (1).

$$D = \frac{k_B\, T}{6\, \pi\, \mu\, r_H} \quad (1)$$

Here, D is the calculated diffusion coefficient based on DOSY experiments, k_B is the Boltzman constant (1.38 * 10^{-23} m^2 kg s^{-2} K^{-1}),T is the temperature, μ is the solution viscosity and r_H is the hydrodynamic radius. For the viscosity (μ) of the 1:1 D_2O/TFE-d_3 mixture a literature reported value (η = 1.78*10^{-3} m^2 s^{-1}) of the undeuterated 1:1 solvent mixture was used.[S7]

Mass spectrometry
High resolution electrospray ionization (HR-ESI) mass spectra were measured on an ESI micrOTOF focus or an ESI micrOTOF-Q III mass spectrometer (Bruker Daltonics). MALDI TOF (Matrix-assisted laser desorption/ionisation time-of-flight) mass spectrometry measurements were acquired on a Bruker Daltonics UltrafleXtreme mass spectrometer using DCTB (*trans*-2-[3-(4-tert-butylphenyl)-2-methyl-2-propenylidene] malononitrile) as matrix. All mass spectra were reported in positive reflector mode.

Melting points
Melting points were measured with an BX41 optical microscope (Olympus) connected to a temperature controller TP94 (Linkam Scientific Instruments Ltd.) and are uncorrected.

Elemental analysis
Elemental analysis was performed with a Vario MICRO cube (Elementar Analysensysteme).

UV/Vis absorption and emission spectroscopy
Spectroscopic measurements were recorded on a Jasco V-670 spectrometer under ambient conditions (298 K) in 1 cm quartz cuvettes using spectroscopic grade solvents.

Electrochemistry
Cyclic and differential pulse voltammetry (CV and DPV) experiments were performed on a BAS Cell Stand C3 (BAS Epsilon) using a glassy carbon disc as working electrode, a Pt wire as counter electrode and Ag/AgCl (3 M KCl) as reference electrode. The measurements were conducted at a scan rate of 100 mV s^{-1} and 20 mV s^{-1} at 298 K. All experiments were measured in homogenous phase in 4:6 mixtures of 2,2,2-trifluorethanol/H_2O (pH 7, 50 mM phosphate buffer). All measured potential are reported vs. normal hydrogen electrode (NHE) by addition of +0.21 V.[S8] The respective Pourbaix diagrams were created based on multiple DPV measurements in phosphate buffered aqueous solution at different pH values (I = 0.1 M) in presence of 40% TFE.

Single crystal X-ray analysis
Single crystals of dimer **2C** and tetramer **4C** were grown by slow crystallization in a TFE-d_3/D_2O solution (1:1) of the respective compound used for NMR experiments and stored under ambient conditions. Single crystals of trimer **3C** were obtained by slow diffusion of diethyl ether either in a DCM/MeOH/TFE (1:1:1) solution of the complex stored in the fridge. Single crystal X-ray diffraction data for **2C**—**4C** were collected at the P11 beamline at DESY. The diffraction data were collected by a single 360° ϕ scan at 100 K. The diffraction

data were indexed, integrated, and scaled using the XDS program package.[S9] For **2C**, in order to compensate low completeness due to single-axis measurement two data sets were merged using the XPREP program from Bruker.[S10] The structures were solved using SHELXT,[S11] expanded with Fourier techniques and refined using the SHELX software package.[S12] Hydrogen atoms were assigned at idealized positions and were included in the calculation of structure factors. All non-hydrogen atoms in the main residue were refined anisotropically. Heavily disordered solvent molecules that cannot be modelled satisfactorily were treated by the SQUEEZE[S13] routine implemented in the program package PLATON.[S14] Other disordered solvent molecules and disordered parts in the main residue were modelled with constraints and restraints using standard SHELX commands EADP, FLAT, SAME, RIGU, DELU, DFIX, DANG, and ISOR. The diffraction data for **4C** was analysed as twins using HKLF5 data generated by the TwinRotMat function implemented in PLATON.[S14] Crystallographic data for the structures reported in this Article have been deposited at the Cambridge Crystallographic Data Centre under deposition numbers CCDC 2214081 (**2C**), 2214083 (**3C**) and 2214082 (**4C**) Copies of these data can be obtained free of charge from the Cambridge Crystallographic Data Centre via www.ccdc.cam.ac.uk/structures/.

Photocatalytic water oxidation
Photocatalytic water oxidation experiments were conducted after a standardized procedure following our previous publications.[S15–S18] An Oxygraph Plus Clark-electrode system with a transparent and temperature-controlled reaction chamber (Hansatech Instruments Ltd.) was used for oxygen detection. For sample irradiation, a complete plug and play light source system including a 150 W Xenon arc lamp equipped with a cutoff filter (400 nm, Thorlabs) and an arc lamp power supply (Newport) was used. Prior to measurement, the power of the sun simulation was calibrated to an intensity of 100 mW cm^{-1} applying a collinear light path with collimated light. The calibration was performed with a PM 200 optical power meter equipped with a S121C sensor (Thorlabs), which was installed in a modified oxygen chamber (Hansatech Instruments Ltd.) combined with a CCS 200/M wide range spectrometer (Thorlabs). All experiments were conducted under the same operation conditions (V = 2 mL, T = 20 °C, stirring speed = 100 rpm). Following a standard procedure, a stock solution of photosensitizer (PS) $[Ru(bpy)_3]Cl_2$ and $Na_2S_2O_8$ as sacrificial electron acceptor (SEA) in 4:6 CH_3CN/H_2O mixture (pH 7, 50 mM phosphate buffer) was prepared in the dark ($c(Na_2S_2O_8)$ = 37 mM, $c([Ru(bpy)_3]Cl_2)$ = 1.5 mM) before each experiment. Then, an aliquot of this solution (1.5 mL) was transferred to the reaction chamber and mixed with varying amount of catalyst concentration (0.5 mL) in the dark. Irradiation of the sample was started after the baseline was constant (~50 s). For data evaluation, a blank measurement in absence of catalyst was subtracted from each measurement of concentration-dependent experiments. To benchmark the novel catalysts, the respective turnover frequency (TOF) and turnover number (TON) were determined. The TON, which is defined as total amount of evolved oxygen divided by the amount of used catalyst, is calculated for each concentration and the highest TON is reported. The TOF for each concentration can be determined by linear regression fit of the first 5−10 s of catalysis from the plot of evolved oxygen vs. reaction time. The extracted value represents the initial rate of catalysis for the respective concentration. The averaged, reported TOF was then determined from the slope of a linear regression of the initial rates of each concentration vs. the respective catalyst amount.

Chemical water oxidation
In accordance to previous publications,[S15–S18] chemical water oxidation experiments were carried out in Schlenk reaction vessels (V = 20.6 mL) connected to pressure transducers (Honeywell, SSCDANN030PAAA5, absolute pressure, 0 to 30 psi) at 20 °C and a constant stirring speed (1000 rpm). For each concentration-dependent measurement, cerium ammonium nitrate (CAN, 1.0 g, 1.82 mmol) was dissolved in 4:6 CH_3CN/H_2O mixture (3.0 mL, pH 1, triflic acid) and then a stock solution of the catalyst (400 µL) was injected through a septum. To determine the turnover number (TON), the total amount of evolved oxygen was divided by the amount of injected catalyst. The total amount of evolved oxygen during catalysis was calculated by applying the ideal gas law:

$$\Delta p \times V = \Delta n \times R \times T \qquad (1)$$

with T = 293.15 K, V = 20.6 mL, R = 8.314 J K^{-1} mol^{-1}.

Based on the measured pressure increase in the reaction vessel, the pressure increase (Δp) can be converted into the amount of generated oxygen (Δn). A TON was calculated for each concentration and the highest TON is reported. From the same concentration-dependent measurements, a TOF was calculated for each concentration from the obtained initial rates by linear regression of the oxygen evolution curve during the first two seconds of catalysis. The reported TOF was then determined from the slope of a linear regression of the initial rates against the catalyst amount.

Catalyst stability test
To test the stability of the catalyst after water oxidation catalysis, each compound (10.0 mg, 3.0 µmol) was dissolved in 4:6 CH_3CN/H_2O mixture (1.0 mL, pH 1, triflic acid). After addition of CAN (500 mg, 960 µmol), vigorous oxygen evolution occurred (~40 catalytic cycles). Subsequently, ammonium hexafluorophosphate (ca. 250 mg) was added and a precipitate was formed. After filtration and several washing with H_2O, the sample was dried under high vacuum. The oxidized catalyst was further investigated by MALDI TOF mass spectrometry (positive, DCTB, CH_2Cl_2/CH_3OH 1:1) in presence of ascorbic acid.

Kinetic Isotope Experiments
Using our standardized procedures,[S15,S16,S18] the experiments were performed using a Oxygraph Plus Clark-electrode system (Hansatech Instruments Ltd.) for oxygen detection (T = 20 °C, stirring speed = 100 rpm).
Kinetic Isotopic effect under conditions of photocatalytic water oxidation: Experiments were performed in accordance with the procedures described for photocatalytic water oxidation. A stock solution of PS ($c([Ru(bpy)_3]Cl_2)$ = 1.5 mM) and SEA ($c(Na_2S_2O_8)$ = 37 mM) in 4:6 CH_3CN/H_2O (H_2O or D_2O (99.9% purity); pH 7, 50 mM phosphate buffer) was prepared in the dark. An aliquot of this solution (1.5 mL) was mixed with catalyst solution at varying concentrations (0.5 mL) in the dark. Irradiation was started at 50 s to allow thermal equilibration of the sample at a constant temperature of 20 °C.
To evaluate the catalytic rates in H_2O ($k(H_2O)$) and D_2O ($k(D_2O)$), the initial rate of catalysis for each concentration was determined by linear regression of the evolved oxygen curve during the first five to ten seconds of catalysis.

Experimental Procedures

6,6'-bis(pyridin-3-yloxy)-2,2'-bipyridine (3)

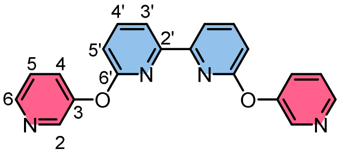

3

A mixture of 6,6-dibromo-2,2'-bipyridine (**1**) (314 mg, 1.00 mmol, 1.0 equiv.), 3-hydroxypyridine (**2**) (285 mg, 300 mmol, 3.0 equiv.), Cs_2CO_3 (977 mg, 3.00 mmol, 3.0 equiv.), CuI (57.1 mg, 300 μmol, 0.3 equiv.) and $Co(acac)_2$ (77.2 mg, 300 μmol, 0.3 equiv.) were suspended in *N*-methyl-2-pyrrolidon (3 mL) under nitrogen and the resulting mixture was stirred for 20 hours at 110 °C. After cooling down to room temperature, water (50 mL) was added, and the aqueous phase was extracted with ethyl acetate (4 x 50 mL). After removing the solvent under reduced pressure, the crude product was suspended in water and filtrated. The filtrate was further purified by flash chromatography (SiO_2, ethyl acetate/methanol 99:1 to 90:10) to yield ligand **3** as a light-brown solid (256 mg, 748 μmol, 75%). **m.p.** 112 °C. **^{1}H NMR** (400 MHz, $CDCl_3$): *δ* [ppm] = 8.61 (m, 2H, *H2*), 8.49 (m, 2H, *H6*), 7.77 – 7.70 (m, 4H, *H4*' & *H5*'), 7.58 (ddd, $^3J_{H-H}$ = 8.3 Hz, $^4J_{H-H}$ = 2.7 Hz, 1.4 Hz, 2H, *H4*), 7.38 (dd, $^3J_{H-H}$ = 8.3 Hz, $^4J_{H-H}$ = 4.7 Hz, 2H, *H5*), 6.95 (dd, $^3J_{H-H}$ = 7.5 Hz, $^4J_{H-H}$ = 1.4 Hz, 2H, *H3*'). **^{13}C NMR** (100 MHz, $CDCl_3$) *δ* [ppm] = 162.1, 153.1, 150.6, 145.7, 143.9, 140.8, 129.0, 124.0, 116.2, 112.0. **HRMS** (ESI-TOF, pos. mode, $MeCN/CHCl_3$ (1:1)): *m/z* calcd for $C_{20}H_{14}N_4O_2+H^+$: 343.1195 [*M*+H]$^+$; found: 343.1197. **Elemental analysis** calcd. (%) for $C_{20}H_{14}N_4O_2$: C 70.17, H 4.12, N 16.37; found: C 69.93, H 4.15, N 16.26.

Synthesis of [Ru(bda)(6,6'-bis(pyridin-3-yloxy)-2,2'-bipyridine)]$_n$ (n = 2–4), (dimer 2C, trimer 3C and tetramer 4C)

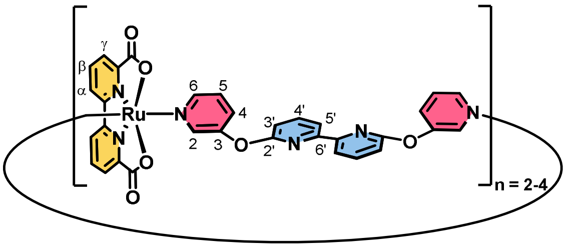

2C–4C

$[Ru(bda)(dmso)_2]$ **1** (165 mg, 330 μmol, 1.1 equiv.) and ligand **3** (103 mg, 300 μmol, 1.0 equiv.) were dissolved in a degassed mixture of chloroform (50 mL) and methanol (50 mL) and stirred under nitrogen at 60 °C for 22 hours . After cooling to room temperature, the solvent was removed under reduced pressure. The residue was purified by size-exclusion chromatography (Bio-Beads S-X1, dichloromethane/methanol 9:1) and three fractions were isolated. The last fraction corresponded to dimer **2C**. As second red fraction, trimer **3C** was collected. Tetramer **4C** was isolated as first broad fraction and was further purified by subsequent recycling gel permeation chromatography (GPC, chloroform/methanol 9:1) to yield pure **4C**.

Dimer **2C** (n=2): **Yield**: 81.0 mg, 59.1 μmol (39%) of a red powder. **m.p.** >300 °C. **^{1}H NMR** (400 MHz, DMSO−d_6): *δ* [ppm] = 9.27 (s, 4H, *H2*), 8.79 (dd, $^3J_{H-H}$ = 6.6 Hz, $^4J_{H-H}$ = 2.6 Hz, 4H, *H3*'), 8.57 (d, $^3J_{H-H}$ = 7.6 Hz, 4H, *Hα*), 8.26 (t, $^3J_{H-H}$ = 7.8 Hz, 4H, *Hβ*), 7.98 – 7.91 (m, 8H, *H4*' & *H5*'), 7.61 (ddd, $^3J_{H-H}$ = 8.4 Hz, $^4J_{H-H}$ = 2.6 Hz, $^5J_{H-H}$ = 1.1 Hz, 4H, *H6*), 7.17 (dd, $^3J_{H-H}$ = 8.4 Hz, $^4J_{H-H}$ = 5.6 Hz, 4H, *H5*), 7.10 (dd, $^3J_{H-H}$ = 8.0 Hz, $^5J_{H-H}$ = 0.5 Hz, 4H, *Hγ*), 6.59 (d, $^3J_{H-H}$ = 5.4 Hz, 4H, *H4*). **^{13}C NMR** (100 MHz, DMSO−d_6) *δ* [ppm] = 172.0, 159.8, 159.3, 156.2, 152.4, 149.0, 148.5, 145.1, 142.7, 131.4, 127.91, 126.1, 125.0, 124.8, 119.5, 111.8. **UV/Vis** (CH_3CN/H_2O 4:6 (pH 7, 50 mM phosphate buffer)): λ_{max} (ε) = 255 (44095), 305 (73722), 347 (24564), 458 (8768), 491 nm (7577 M^{-1} cm^{-1}). **HRMS** (ESI-TOF, pos. mode, $MeCN/CHCl_3$ (1:1)): *m/z* calcd for $C_{64}H_{40}N_{12}O_{12}Ru_2+Na^+$: 1395.0898 [*M+Na*]$^+$; found: 1395.0889. **Elemental analysis** calcd. (%) for $C_{64}H_{40}N_{12}O_{12}Ru_2 \cdot 2H_2O$: C 54.62, H 3.15, N 11.94; found: C 54.17, H 3.21, N 11.67.

Trimer **3C** (n=3): **Yield**: 27.8 mg, 13.5 μmol (14%) of a dark red solid. m.p. >300 °C. **^{1}H NMR** (400 MHz, CD_2Cl_2/CD_3OD (1:1)): *δ* [ppm] = 8.58 (dd, $^3J_{H-H}$ = 8.1 Hz, $^4J_{H-H}$ = 1.0 Hz, 6H, *Hα*), 7.94 (dd, $^3J_{H-H}$ = 7.7 Hz, $^4J_{H-H}$ = 1.0 Hz, 6H, *Hγ*), 7.88 – 7.83 (m, 6H, *H4*'), 7.75 – 7.68 (m, 24H, *Hβ* & *H2* & *H6* & *H5*'), 7.53 (ddd, $^3J_{H-H}$ = 8.5 Hz, $^4J_{H-H}$ = 2.5 Hz, 1.2 Hz, 6H, *H4*), 7.17 – 7.12 (m, 6H, *H5*), 6.76 (dd, $^3J_{H-H}$ = 8.1 Hz, $^4J_{H-H}$ = 0.7 Hz, 6H, *H3*'). **^{13}C NMR** (100 MHz, CD_2Cl_2/CD_3OD (1:1)) *δ* [ppm] = 174.6, 161.3, 160.4, 157.5, 153.2, 152.1, 148.5, 145.8, 142.7, 132.8, 129.4, 126.7, 126.0, 126.0, 118.3, 113.2. **UV/Vis** (CH_3CN/H_2O 4:6 (pH 7, 50 mM phosphate buffer)): λ_{max} (ε) = 255 (58372), 306 (96419), 349 (31993), 456 (12670), 491 nm (10595 M^{-1} cm^{-1}). **HRMS** (ESI-TOF, pos. mode, CH_3OH/CH_2Cl_2 (1:1)): *m/z* calcd for $C_{96}H_{60}N_{18}O_{18}Ru_3+H^{2+}$: 1030.0805 [*M*+H]$^{2+}$; found: 1030.0845. **Elemental analysis** calcd. (%) for $C_{96}H_{60}N_{18}O_{18}Ru_3 \cdot 3H_2O$: C 54.62, H 3.15, N 11.94; found: C 54.18, H 3.29, N 11.67.

Tetramer **4C** (n=4): **Yield**: 5.40 mg, 2.00 μmol (3%) of a dark red solid. m.p. >300 °C. **^{1}H NMR** (400 MHz, CD_2Cl_2/CD_3OD (1:1)): *δ* [ppm] = 8.42 (d, $^3J_{H-H}$ = 8.1 Hz, 8H, *Hα*), 7.87 – 7.80 (m, 16H, *Hγ* & *H4*'), 7.73 (d, $^3J_{H-H}$ = 4.9 Hz, 8H, *H6*), 7.65 (t, $^3J_{H-H}$ = 7.9 Hz, 8H, *Hβ*), 7.60 (d, $^4J_{H-H}$ = 2.4 Hz, 8H, *H2*), 7.55 – 7.52 (m, 8H, *H5*'), 7.47 (ddd, $^3J_{H-H}$ = 8.5 Hz, $^4J_{H-H}$ =2.4 Hz, 1.0 Hz, 8H, *H4*), 7.18 (dd, $^3J_{H-H}$ = 8.4 Hz, 5.6 Hz, 8H, *H5*), 6.78 (dd, $^3J_{H-H}$ = 8.1 Hz, $^4J_{H-H}$ = 0.5 Hz, 8H, *H3*'). **^{13}C NMR** (100 MHz, CD_2Cl_2/CD_3OD (1:1)) *δ* [ppm] = 174.5, 161.8, 160.4, 157.4, 153.3, 152.1, 148.8, 146.0, 142.6, 132.7, 130.0, 126.7, 126.0, 125.9, 118.0, 113.2. **UV/Vis** (CH_3CN/H_2O 4:6 (pH 7, 50 mM phosphate buffer)): λ_{max} (ε) = 255 (65385), 305 (114341), 353 (36989), 457 (15627), 491 nm (13207 M^{-1} cm^{-1}). **HRMS** (ESI-TOF, pos. mode, CH_3OH/CH_2Cl_2 (1:1)): *m/z* calcd for $C_{128}H_{80}N_{24}O_{24}Ru_4^{2+}$: 1371.6005 [*M*]$^{2+}$; found: 1370.5998. **Elemental analysis** calcd. (%) for $C_{128}H_{80}N_{24}O_{24}Ru_4 \cdot 4H_2O$: C 54.62, H 3.15, N 11.94; found: C 54.53, H 3.04, N 11.69.

UV/Vis absorption spectroscopy

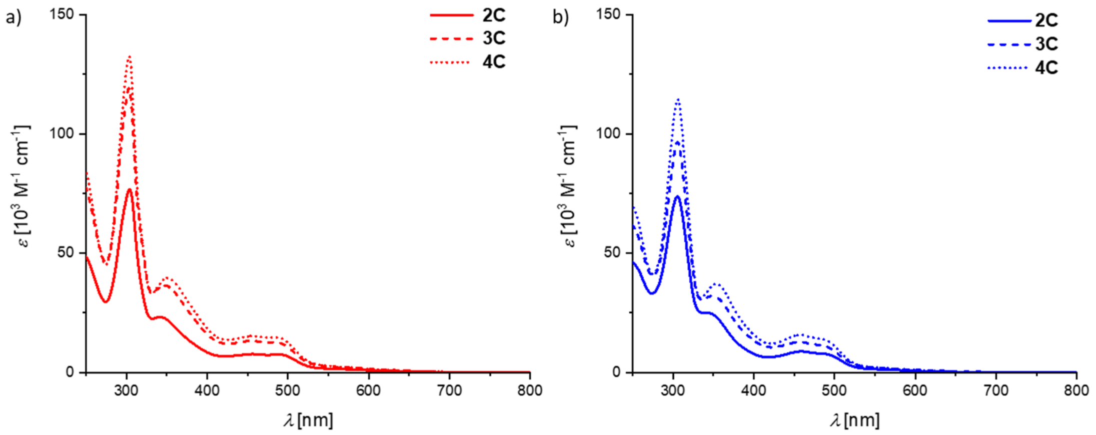

Figure S1. UV/Vis absorption spectra of Ru complexes **2C**—**4C** (c(WOC) = 10^{-5} M) at the Ru^{II} state in CH_3CN/H_2O 4:6 at a) pH 1 (red) and b) pH 7 (blue).

Single crystal X-ray analysis

Table S1. Comparison of selected bond lengths and angles for compounds **2C**—**4C** in the solid state.

	**2C**[a]	**3C**[b]			**4C**	
	Ru	Ru1	Ru2	Ru3	Ru1_1/ Ru1_2	Ru2_1/ Ru2_2
O-Ru-O [°]	121.7(2)/122.7(2)	121.8(8)	121.9(2)	121.1(1)	122.5(3)	122.0(2)
N_{ax}-Ru-N_{ax} [°]	171.3(2)/172.4(2)	170.8(7)	175.0(2)	169.9(6)	170.8(2)	171.3(2)
d (Ru-N_{ax}) [Å]	2.072(6)/2.064(5)	2.074(2)	2.050(2)	2.066(2)	2.079(6)	2.074(6)
	2.089(6)/2.067(5)	2.087(2)	2.104(2)	2.074(2)	2.087(6)	2.077(6)
Torsion py_{ax}−Ru−py_{ax} [°]	30.6/27.9	45.4	27.8	15.1	13.7(9)	27.9(9)

[a] Crystallographic data are given for both asymmetric conformers of the macrocycle within the unit cell. [b] Crystallographic data are given for the main Ru2(bda) unit of the macrocycle within the unit cell.

Contents in an asymmetric unit

2C: Each asymmetric unit contains half of two crystallographic isomers of dinuclear Ru(bda) complexes, four TFE solvent molecules and half ascorbic acid molecule.
3C: Each asymmetric unit contains one macrocyclic Ru(bda) complex with a disordered Ru2(bda) unit. In addition, each asymmetric unit contains 0.43 DCM solvent molecules and 0.84 TFE solvent molecules.
4C: Each asymmetric unit contains half of two similar but crystallographically independent tetranuclear Ru(bda) complexes and eight TFE solvent molecules.

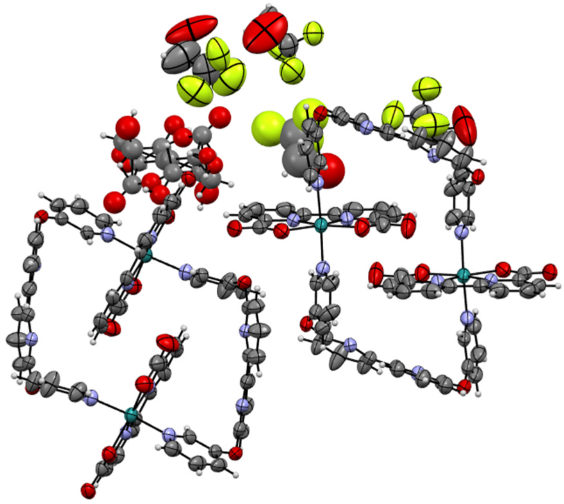

Figure S2. ORTEP diagram (thermal ellipsoids set at 50% probability; C, grey; H, white, O, red, N, purple; Ru, turquoise) for dimer **2C**.

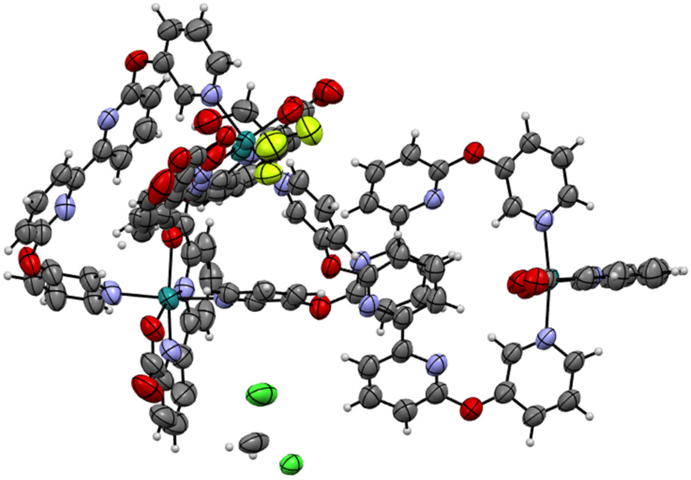

Figure S3. ORTEP diagram (thermal ellipsoids set at 50% probability; C, grey; H, white, O, red, N, purple; Ru, turquoise) for trimer **3C**.

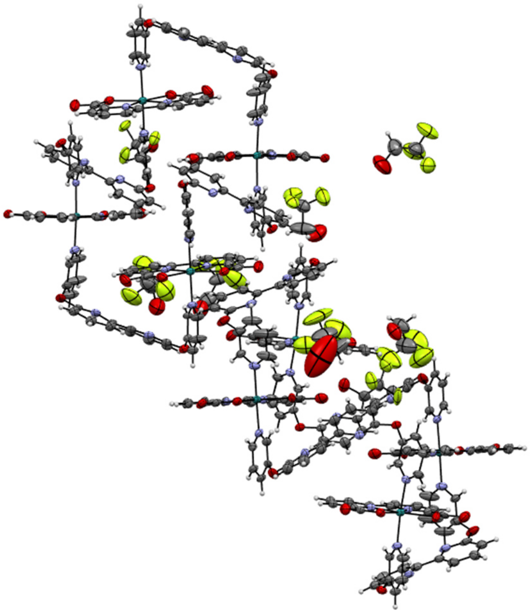

Figure S4. ORTEP diagram (thermal ellipsoids set at 50% probability; C, grey; H, white, O, red, N, purple; Ru, turquoise) for tetramer **4C**.

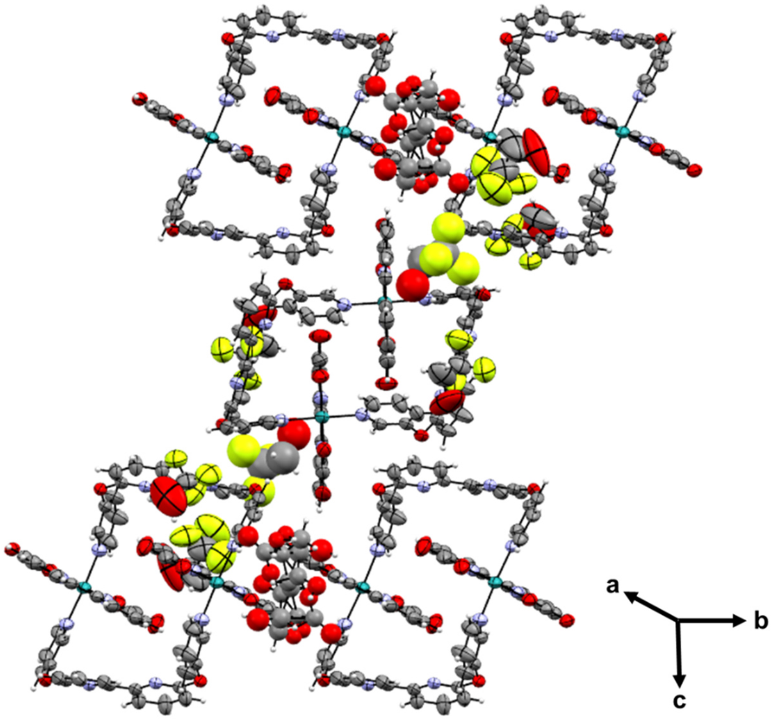

Figure S5. Crystal packing of **2C** viewed along the cell axis *a* with TFE solvent and ascorbic acid molecules in the crystal lattice.

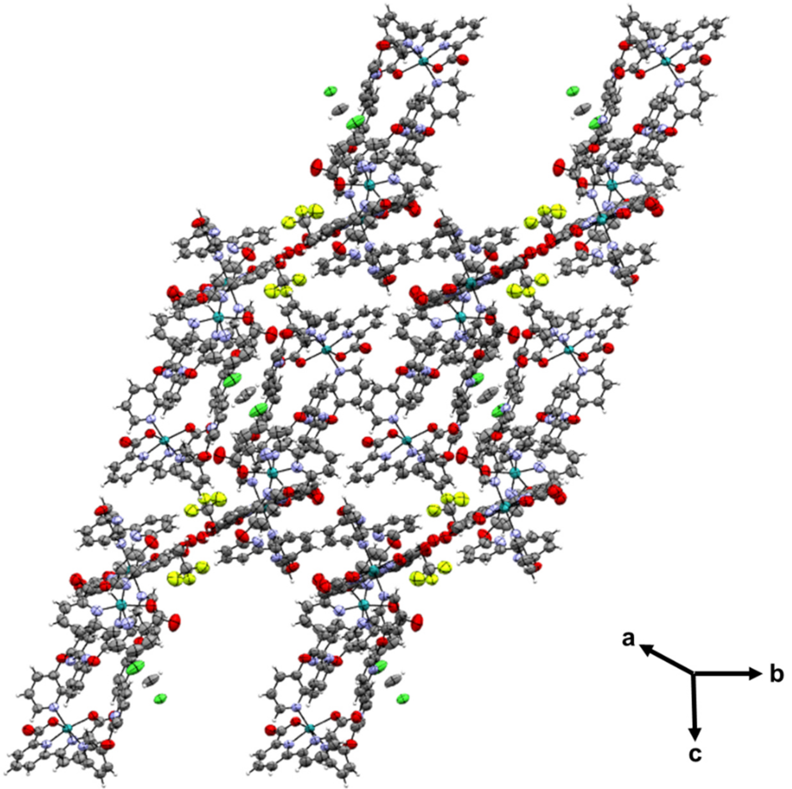

Figure S6. Crystal packing of **3C** viewed along the cell axis *a* with TFE and DCM solvent molecules in the crystal lattice.

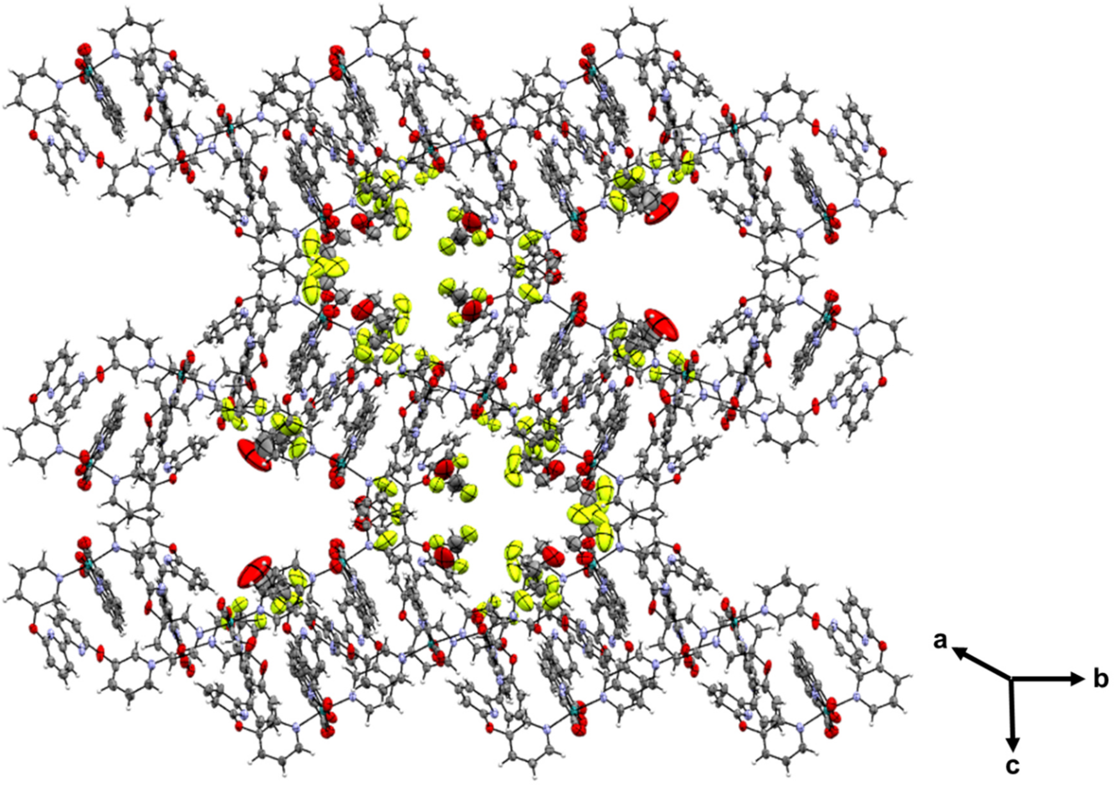

Figure S7. Crystal packing of 4**C** viewed along the cell axis *a* with TFE solvent molecules in the crystal lattice.

Table A2. Crystal data and structure refinement for compounds **2C**—**4C**.

Compound	**2C**	**3C**	**4C**
CCDC Number	2214081	2214083	2214082
Empirical formula	$C_{75}H_{52}F_{12}N_{12}O_{19}Ru_2$	$C_{98.11}H_{63.38}Cl_{0.86}F_{2.52}N_{18}O_{18.84}Ru_3$	$C_{144}H_{96}F_{24}N_{24}O_{32}Ru_4$
M / g mol^{-1}	1855.43	2177.36	3534.73
Temperature / K	100(2)	100(2)	100(2)
Wavelength / Å	0.61991	0.61992	0.61991
Crystal system, space group	Triclinic, $P\bar{1}$	Monoclinic, C2/c	Monoclinic, P2/c
Unit cell dimensions:			
a / Å	12.602(3)	44.496(8)	18.882(5)
b / Å	14.511(2)	15.520(5)	32.122(4)
c / Å	24.251(3)	35.529(6)	31.482(4)
α / °	102.832(6)	90	90
β / °	100.374(10)	108.633(6)	92.142(9)
γ / °	102.512(8)	90	90
Volume V / Å^3	4098.2(13)	23249(9)	19081(6)
Z	2	8	4
Calculated density ρ_{cal} / g cm^{-3}	1.504	1.244	1.230
Absorption coefficient / mm^{-1}	0.326	0.327	0.276
F(000)	1868	8784.4	7104
Crystal size / mm^3	0.050 × 0.020 × 0.020	0.100 × 0.100 × 0.100	0.200 × 0.200 × 0.200
Measurement range of Θ / °	0.774 to 28.172	0.842 to 27.672	0.553 to 26.282
Limiting indices	–17<=h<=17, -21<=k<=21, -33<=l<=34	-61<=h<=62, -20<=k<=20, -47<=l<=46	-25<=h<=24, -45<=k<=45, -40<=l<=40
Reflections collected / unique	140045	197369	320182
Independent reflections	23359 [*R*(int) = 0.0705]	31284 [*R*(int) = 0.0334]	52811 [*R*(int) = 0.0314]
Completeness / %	99.1	98.2	98.9
Absorption correction	none	None	none
Refinement method	Full-matrix least-squares on F^2	Full-matrix least-squares on F^2	Full-matrix least-squares on F^2
Data / restraints / parameters	23359 / 302 / 1036	31284 / 423 / 1472	52811 / 720 / 2053
Goodness of fit for F^2	1.019	1.074	1.108
Final *R* indices [$I > 2\sigma(I)$]	R_1 = 0.1040, wR_2 = 0.3202	R_1 = 0.0451, wR_2 = 0.1423	R_1 = 0.1190, wR_2 = 0.3680
R indices (all data)	R_1 = 0.1266, wR_2 = 0.3412	R_1 = 0.0485, wR_2 = 0.1458	R_1 = 0.1216, wR_2 = 0.3696
Extinction cooeficient	n/a	n/a	n/a
Largest diff. peak and hole	1.444 and –1.032 e·Å^{-3}	0.767 and –1.262 e·Å^{-3}	3.345 and –3.307 e·Å^{-3}

NMR experiments @ pD 7

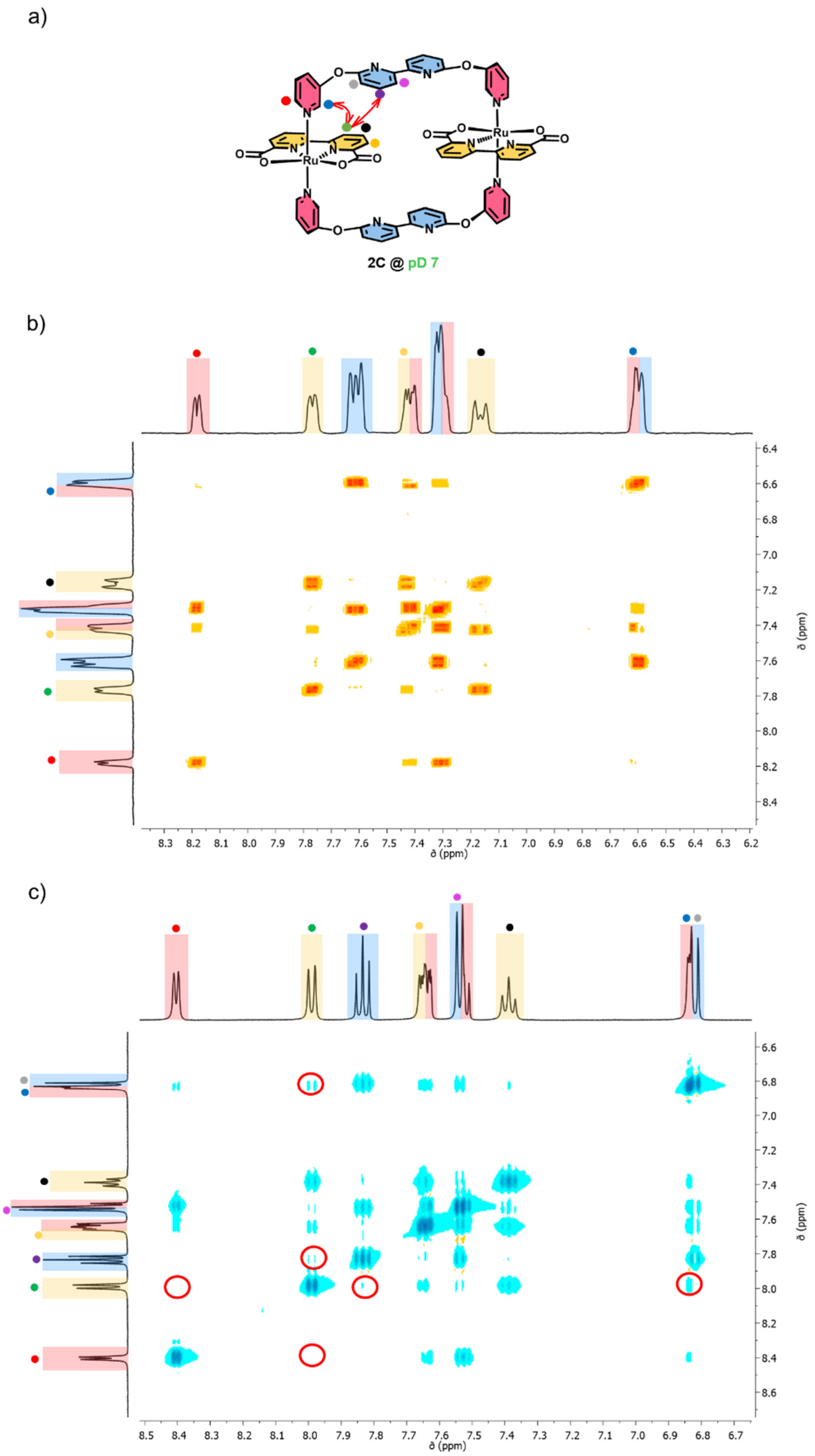

Figure S8. a) Molecular structure of **2C** with specific protons marked in colour. b) ^{1}H-^{1}H COSY NMR spectra (red) and c) ^{1}H-^{1}H NOESY NMR spectra (cyan: negative signal/red: positive signal) spectra of **2C** in TFE-d_3/D_2O 1:1 (pD 7, 400 MHz). The red double-headed arrows in figure a) indicate close spatial proximity between the protons according to the cross peaks marked with red circles in the NOESY spectra of **2C**.

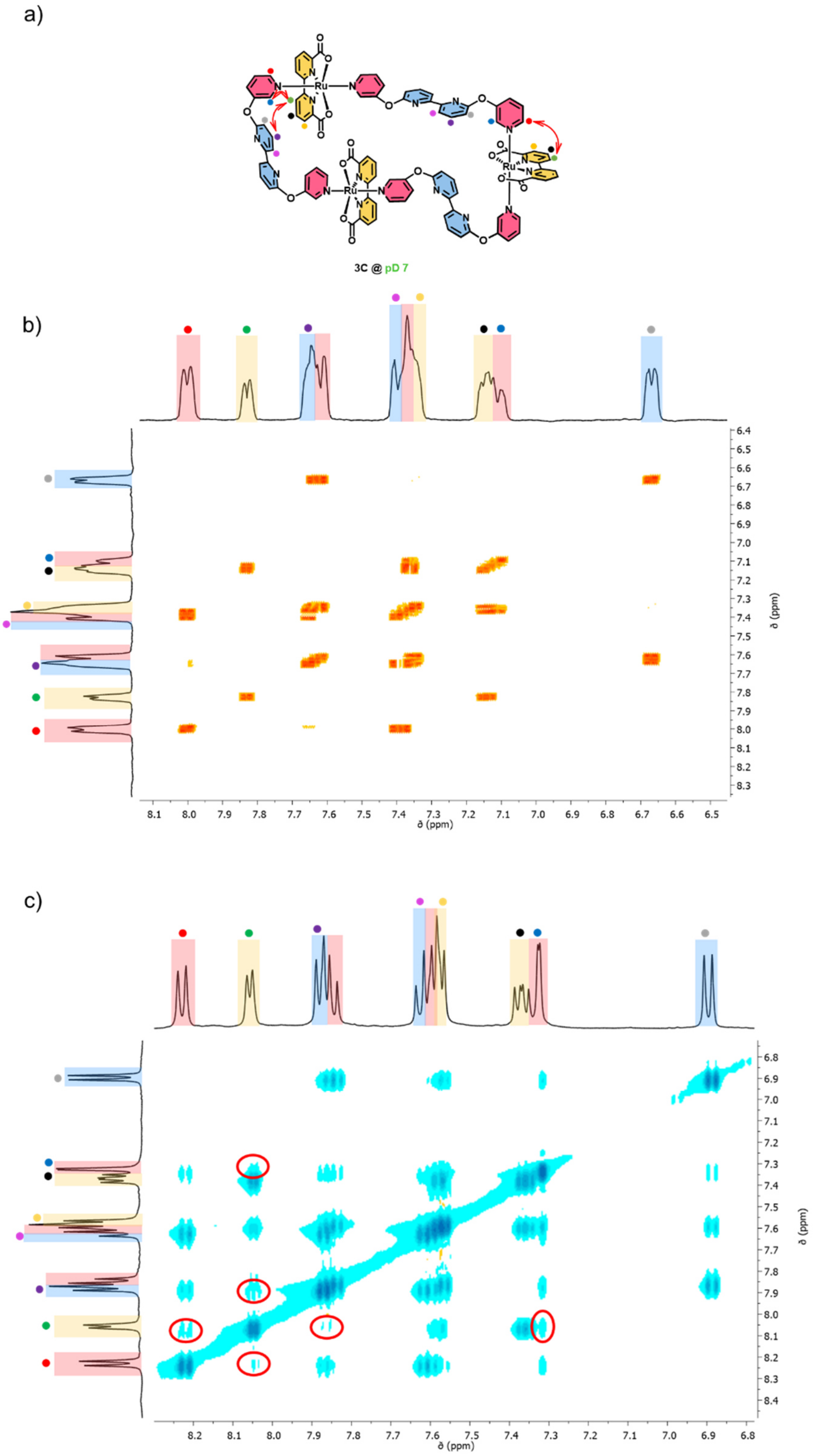

Figure S9. a) Molecular structure of **3C** with specific protons marked in colour. b) ^1H-^1H COSY NMR spectra (red) and c) ^1H-^1H NOESY NMR spectra (cyan: negative signal/red: positive signal) spectra of **3C** in TFE-d_3/D_2O 1:1 (pD 7, 400 MHz). The red double-headed arrows in figure a) indicate close spatial proximity between the protons according to the cross peaks marked with red circles in the NOESY spectra of **3C**.

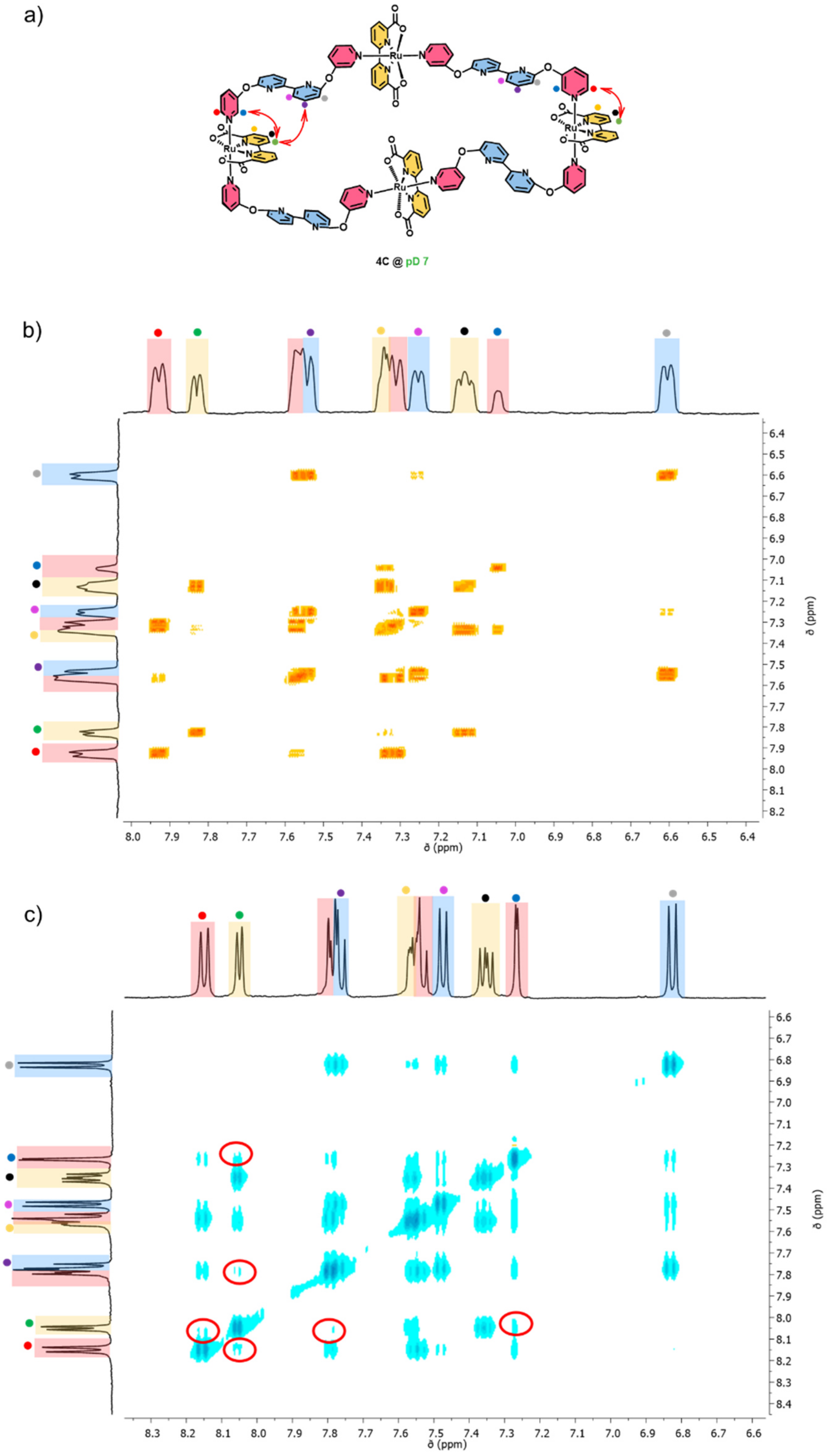

Figure S10. a) Molecular structure of **4C** with specific protons marked in colour. b) ^{1}H-^{1}H COSY NMR spectra (red) and c) ^{1}H-^{1}H NOESY NMR spectra (cyan: negative signal/red: positive signal) spectra of **4C** in TFE-d_3/D_2O 1:1 (pD 7, 400 MHz). The red double-headed arrows in figure a) indicate close spatial proximity between the protons according to the cross peaks marked with red circles in the NOESY spectra of **4C**.

VT–NMR experiments

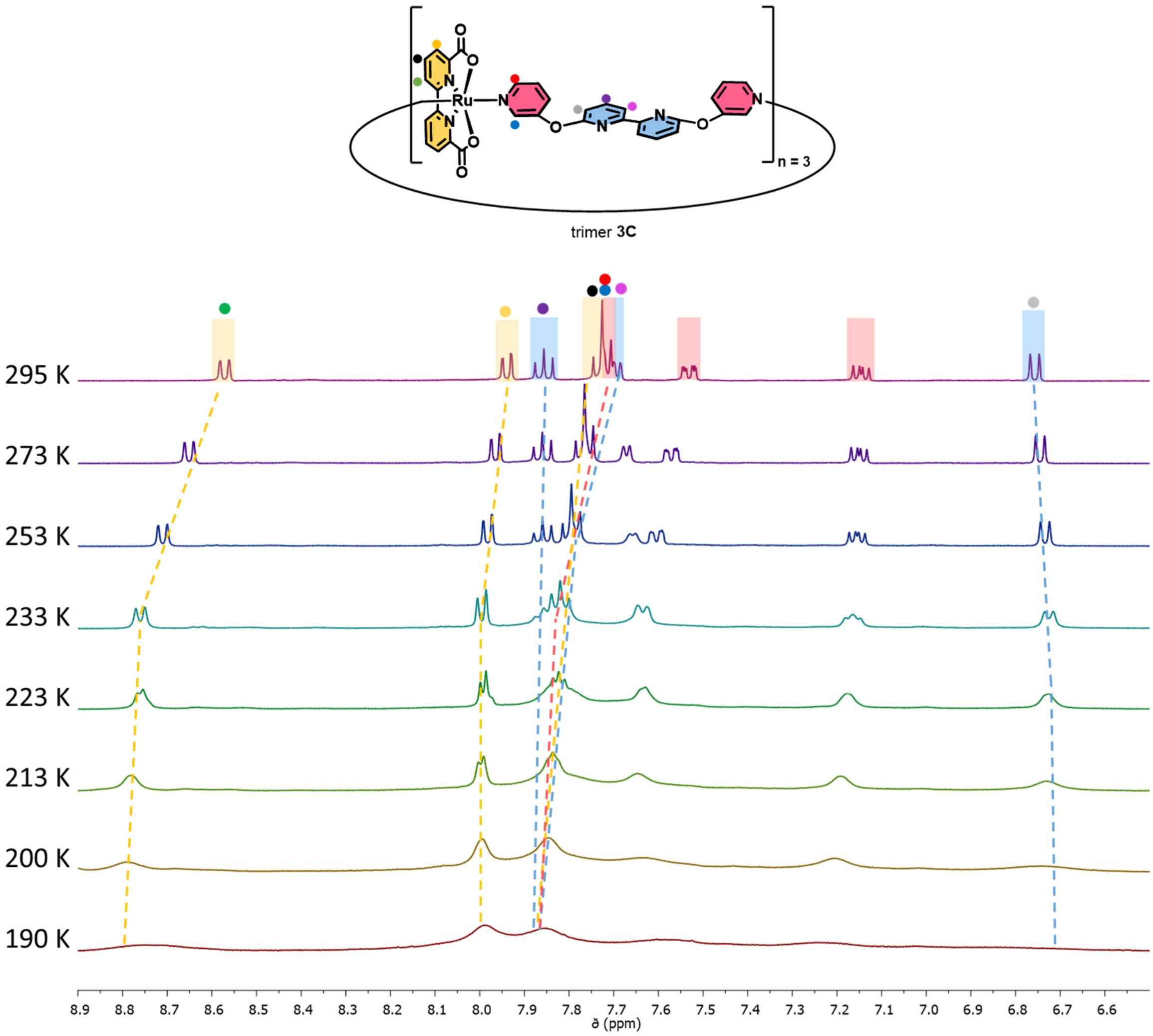

Figure S11. Aromatic region of temperature-dependent ^{1}H NMR spectra of **3C** in CD_2Cl_2/CD_3OD 1:1 (400 MHz) from 295 K to 190 K.

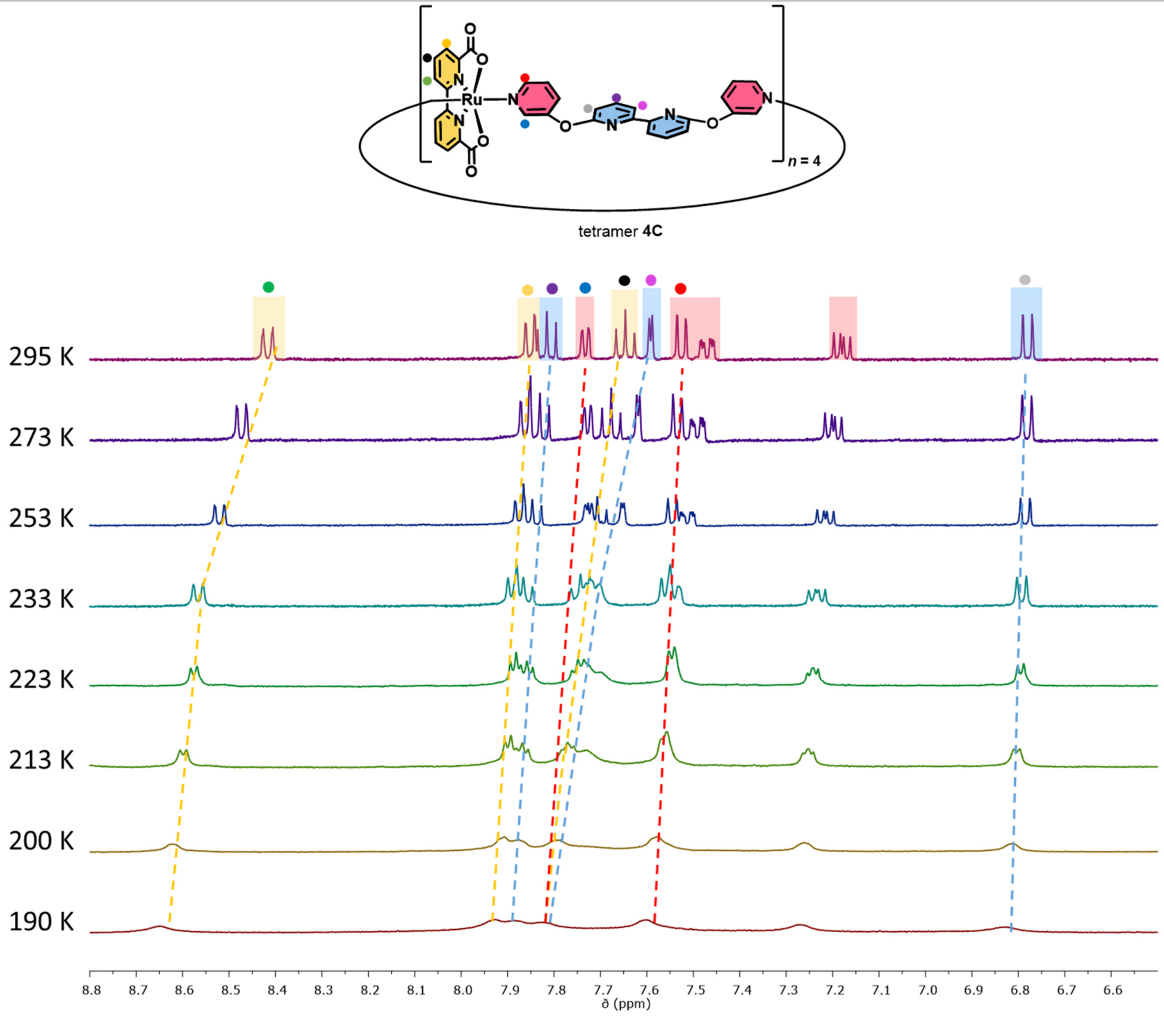

Figure S12. Aromatic region of temperature-dependent ^{1}H NMR spectra of **4C** in CD_2Cl_2/CD_3OD 1:1 (400 MHz) from 295 K to 190 K.

NMR experiments @ pD 1

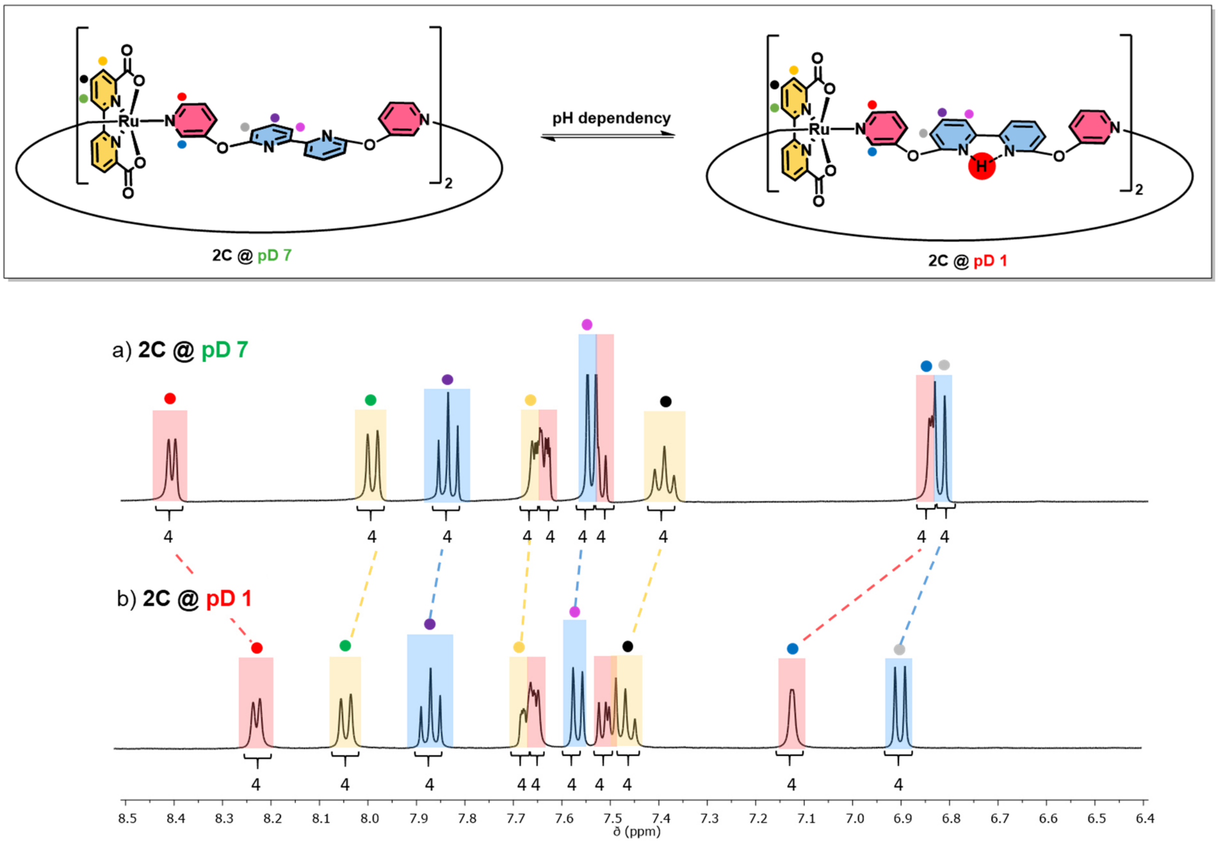

Figure S13. Comparison of the aromatic region of the ^{1}H NMR spectra of complex **2C** in 1:1 TFE-d_3/D_2O ((a) pD 7 and (b) pD 1, 0.1 M CF_3SO_3D, 400 MHz, ascorbic acid, rt). Colors of the signals correspond to bda (yellow), axial pyridine fragment (red) and bipyridine unit (blue) of the axial ligand as highlighted in the structure.

Table S3. Chemical shifts δ (ppm), chemical shift changes Δδ (ppm) and assignment of significant ^{1}H-NMR signals (400 MHz) of complex **2C** in TFE-d_3/D_2O 1:1 (pD 7 or pD 1, 0.1 M CF_3SO_3D).

	axial pyridine		equatorial bda ligand			axial bpy unit		
**2C**	red	blue	green	yellow	black	purple	magenta	grey
pD 7	8.41	6.85	7.99	7.65	7.39	7.83	7.54	6.82
pD 1	8.21	7.11	8.03	7.65	7.44	7.86	7.55	6.89
Δδ	**-0.20**	**+0.26**	**+0.04**	0	**+0.05**	**+0.03**	**+0.01**	**+0.07**

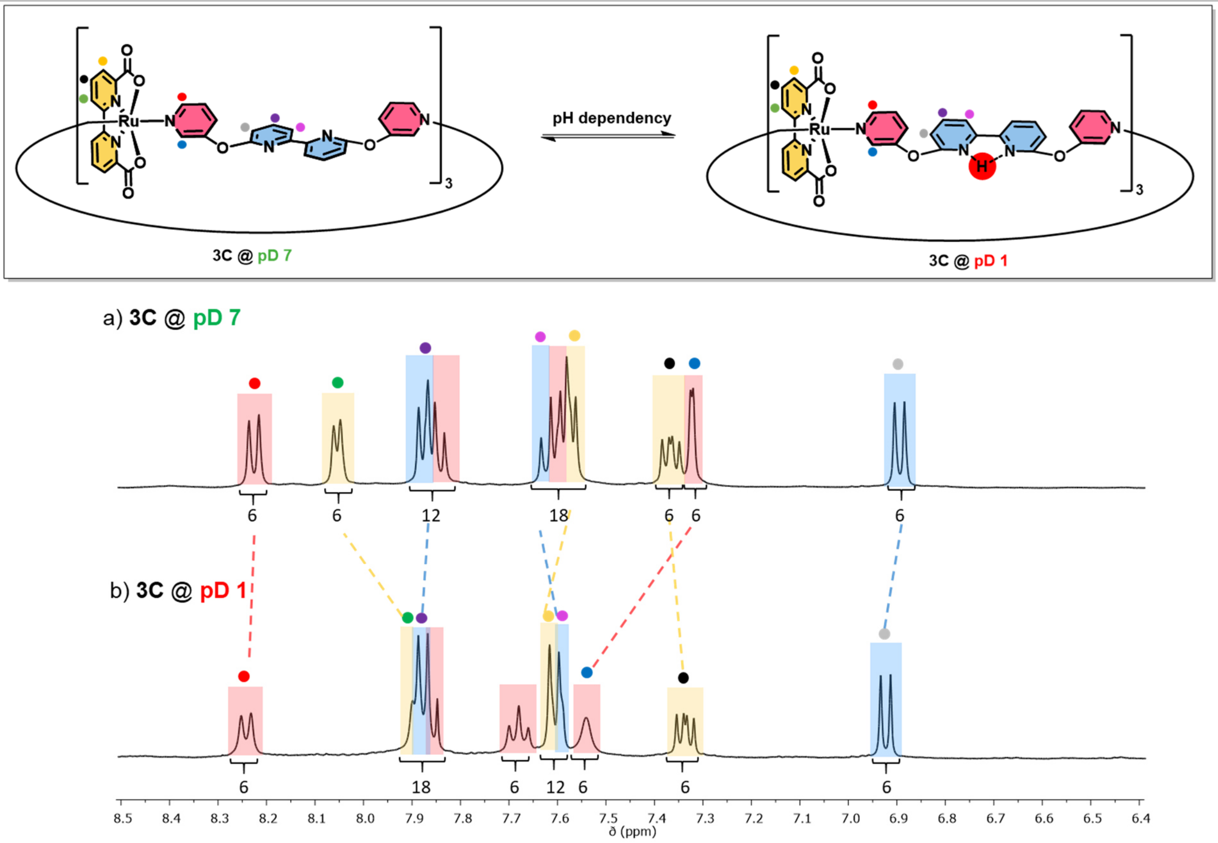

Figure S14. Comparison of the aromatic region of the ^{1}H NMR spectra of complex **3C** in 1:1 TFE-d_3/D_2O ((a) pD 7 and (b) pD 1, 0.1 M CF_3SO_3D, 400 MHz, ascorbic acid, rt). Colors of the signals correspond to bda (yellow), axial pyridine fragment (red) and bipyridine unit (blue) of the axial ligand as highlighted in the structure.

Table S4. Chemical shifts δ (ppm), chemical shift changes Δδ (ppm) and assignment of significant ^{1}H-NMR signals (400 MHz) of complex **3C** in TFE-d_3/D_2O 1:1 (pD 7 or pD 1, 0.1 M CF_3SO_3D).

	axial pyridine		equatorial bda ligand			axial bpy unit		
**3C**	● (red)	● (blue)	● (green)	● (yellow)	● (black)	● (purple)	● (pink)	● (grey)
pD 7	8.23	7.33	8.06	7.57	7.37	7.88	7.63	6.90
pD 1	8.25	7.54	7.90	7.62	7.34	7.89	7.60	6.93
Δδ	**+0.02**	**+0.21**	**-0.16**	**+0.05**	**-0.03**	**+0.01**	**-0.03**	**+0.03**

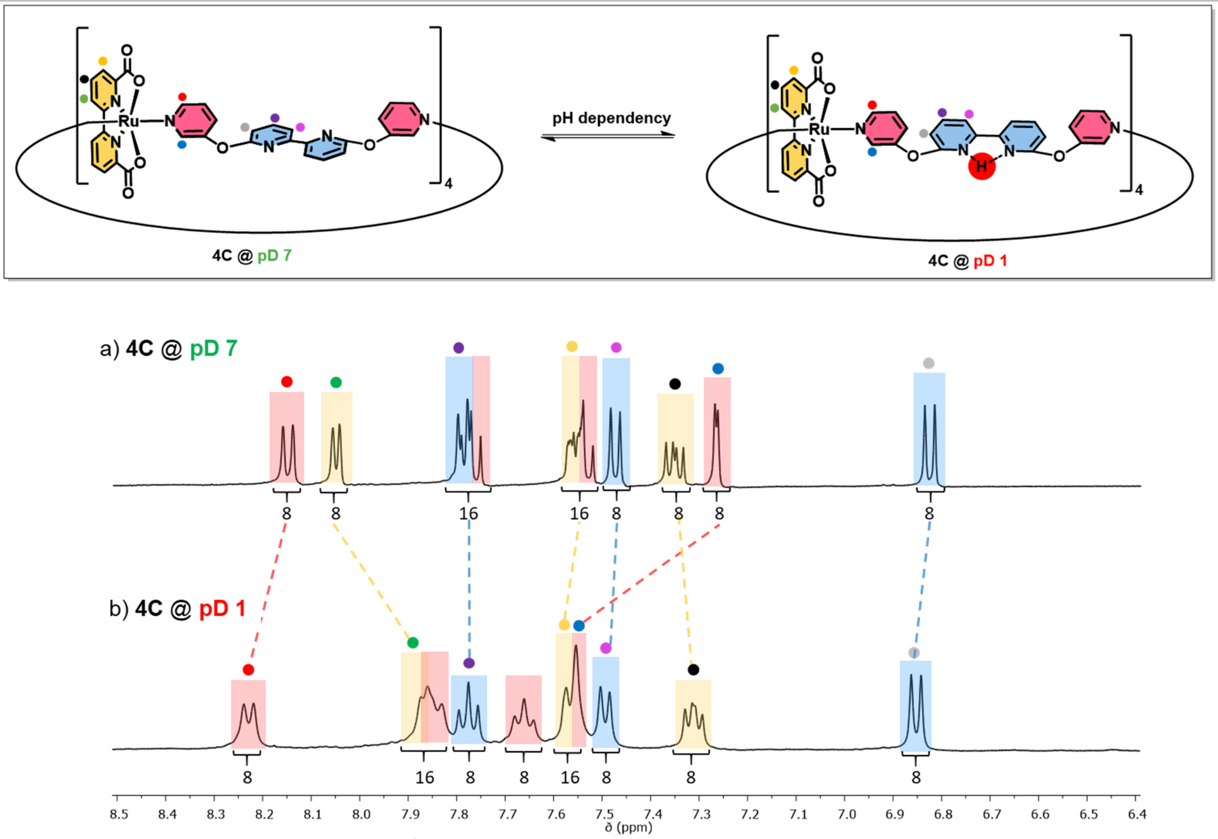

Figure S15. Comparison of the aromatic region of the ^{1}H NMR spectra of complex **4C** in 1:1 TFE-d_3/D_2O ((a) pD 7 and (b) pD 1, 0.1 M CF_3SO_3D, 400 MHz, ascorbic acid, rt). Colors of the signals correspond to bda (yellow), axial pyridine fragment (red) and bipyridine unit (blue) of the axial ligand as highlighted in the structure.

Table S5. Chemical shifts δ (ppm), chemical shift changes Δδ (ppm) and assignment of significant ^{1}H-NMR signals (400 MHz) of complex **4C** in TFE-d_3/D_2O 1:1 (pD 7 or pD 1, 0.1 M CF_3SO_3D).

	axial pyridine		equatorial bda ligand			axial bpy unit		
**4C**	●	●	●	●	●	●	●	●
pD 7	8.15	7.27	8.05	7.57	7.35	7.78	7.47	6.83
pD 1	8.23	7.55	7.87	7.57	7.31	7.77	7.49	6.85
Δδ	**+0.08**	**+0.28**	**-0.18**	**0**	**-0.04**	**-0.01**	**+0.02**	**+0.02**

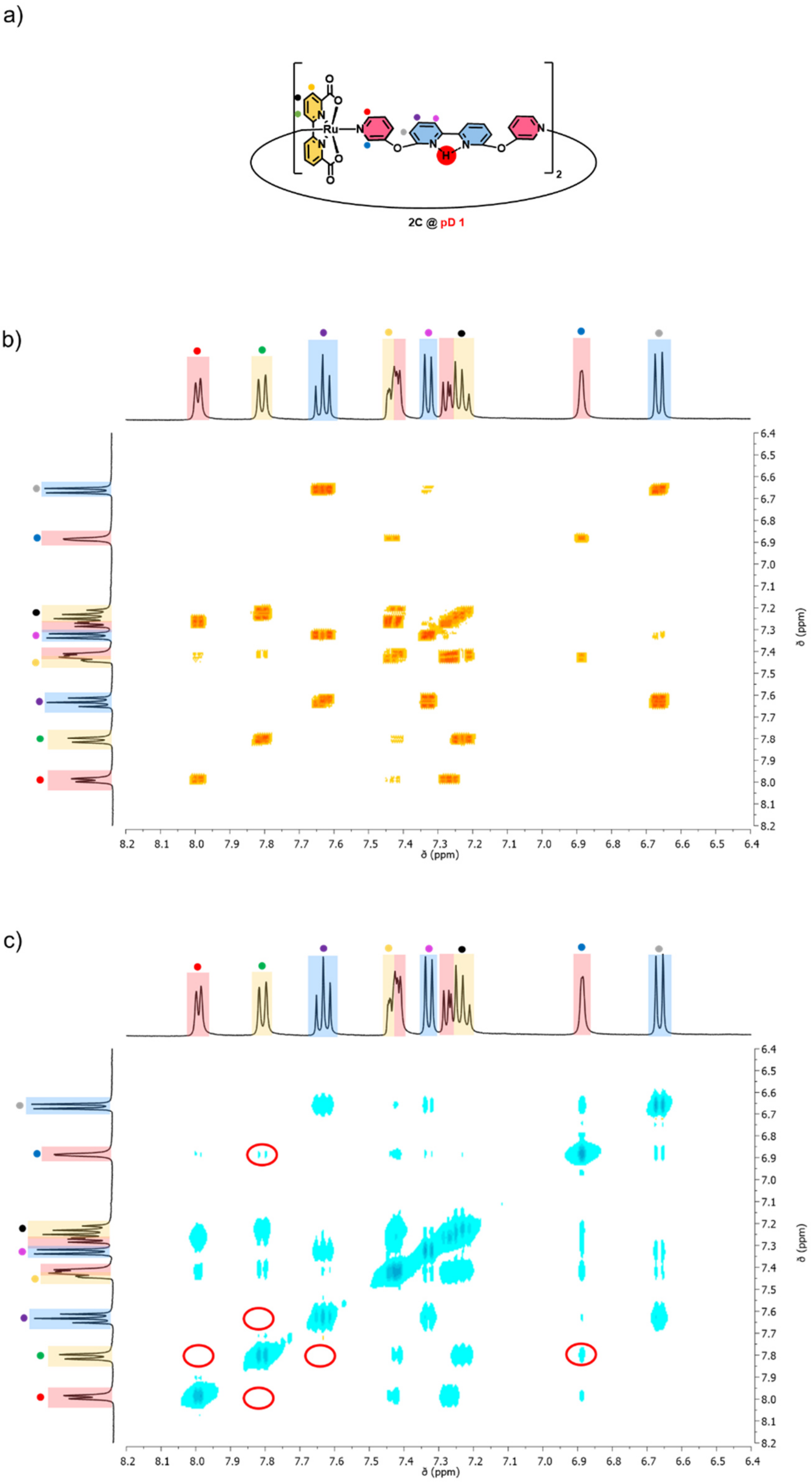

Figure S16. a) Molecular repeating unit of **2C** with specific protons marked in colour. b) ^{1}H-^{1}H COSY NMR spectra (red) and c) ^{1}H-^{1}H NOESY NMR spectra (cyan: negative signal/red: positive signal) spectra of **2C** in TFE-d_3/D_2O 1:1 (pD 1, 0.1 M CF_3SO_3D, 400 MHz).

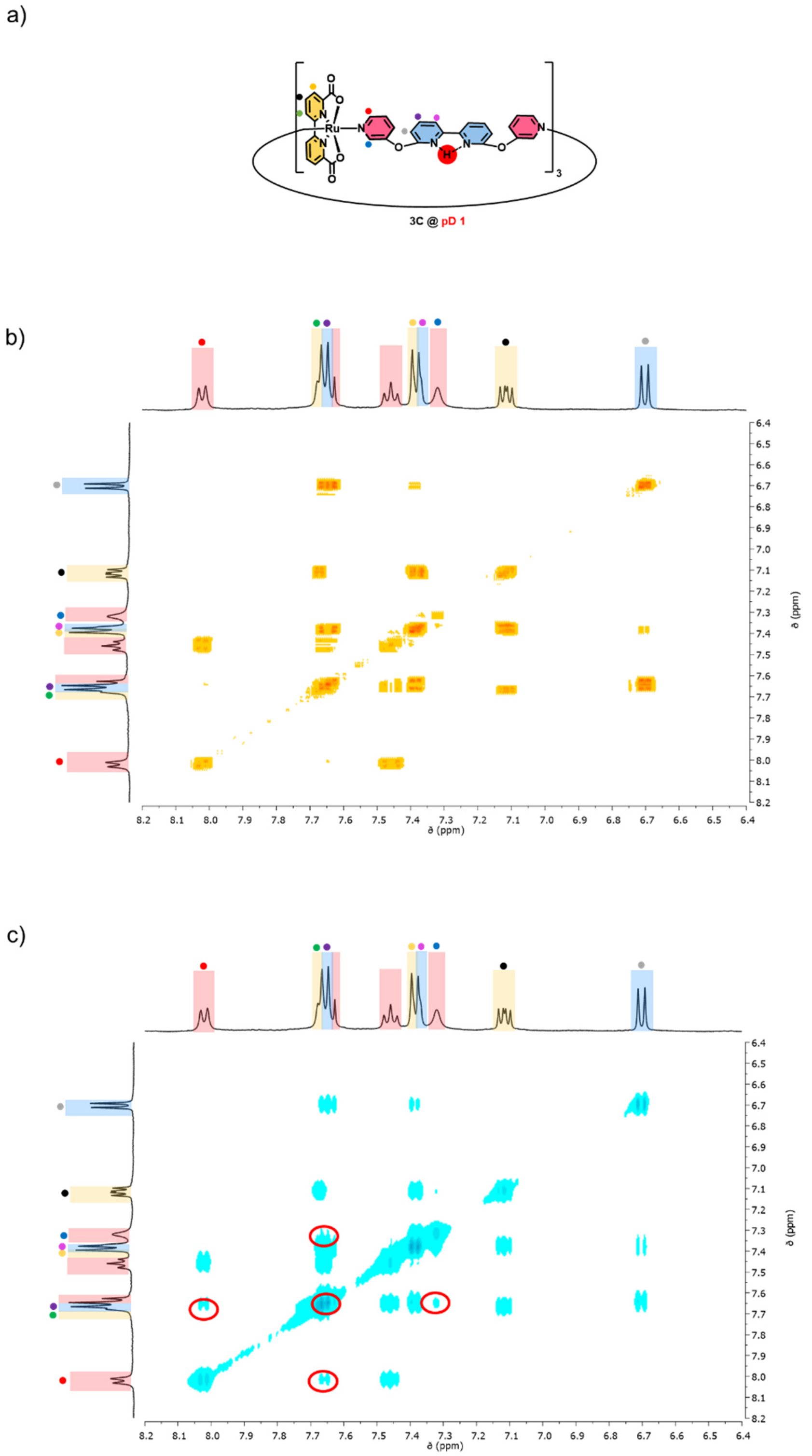

Figure S17. a) Molecular repeating unit of **3C** with specific protons marked in colour. b) ^{1}H-^{1}H COSY NMR spectra (red) and c) ^{1}H-^{1}H NOESY NMR spectra (cyan: negative signal/red: positive signal) spectra of **3C** in TFE-d_3/D_2O 1:1 (pD 1, 0.1 M CF_3SO_3D, 400 MHz).

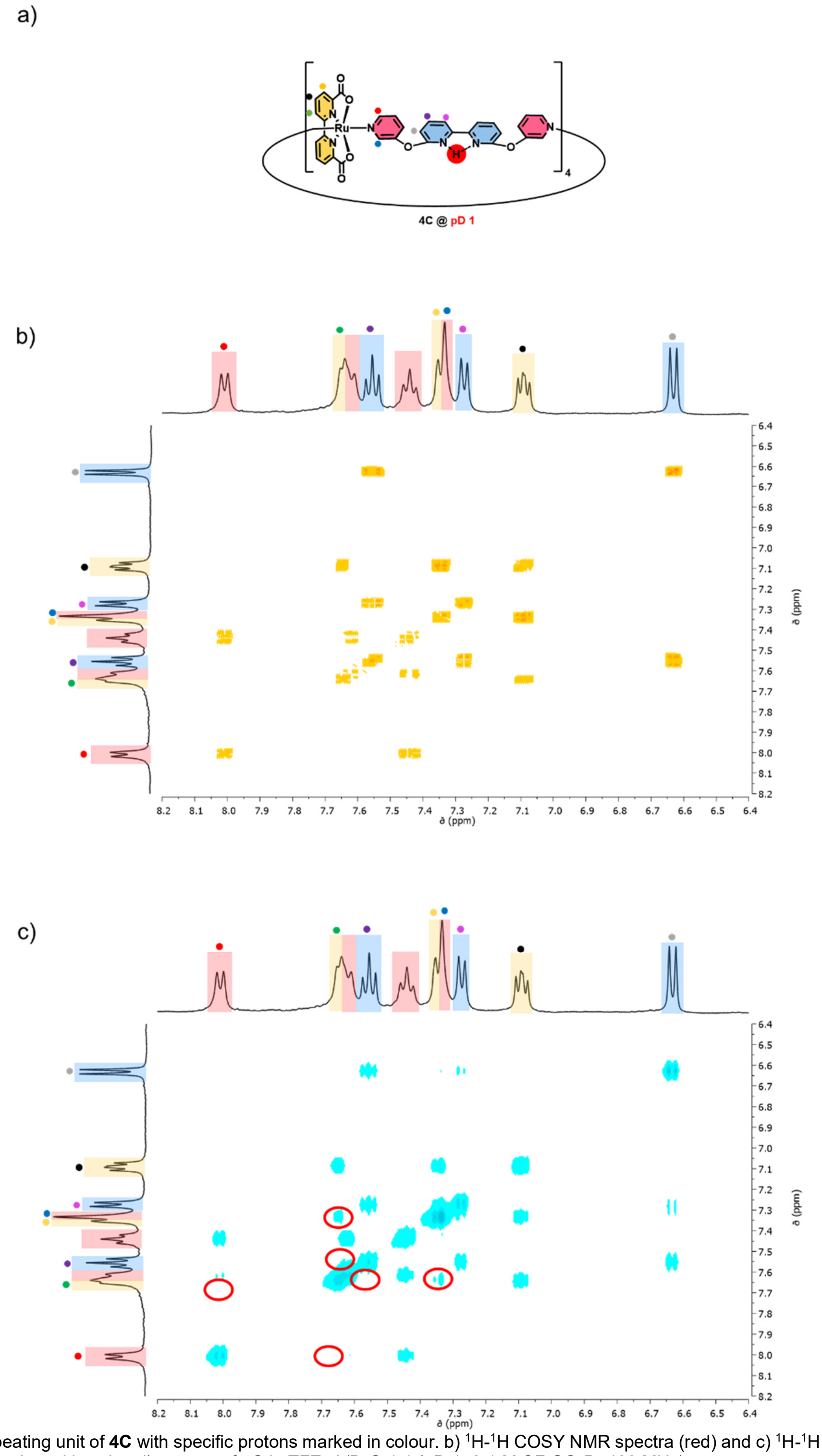

Figure S18. a) Molecular repeating unit of **4C** with specific protons marked in colour. b) ¹H-¹H COSY NMR spectra (red) and c) ¹H-¹H NOESY NMR spectra (cyan: negative signal/red: positive signal) spectra of **4C** in TFE-d_3/D_2O 1:1 (pD 1, 0.1 M CF_3SO_3D, 400 MHz).

DOSY NMR experiments

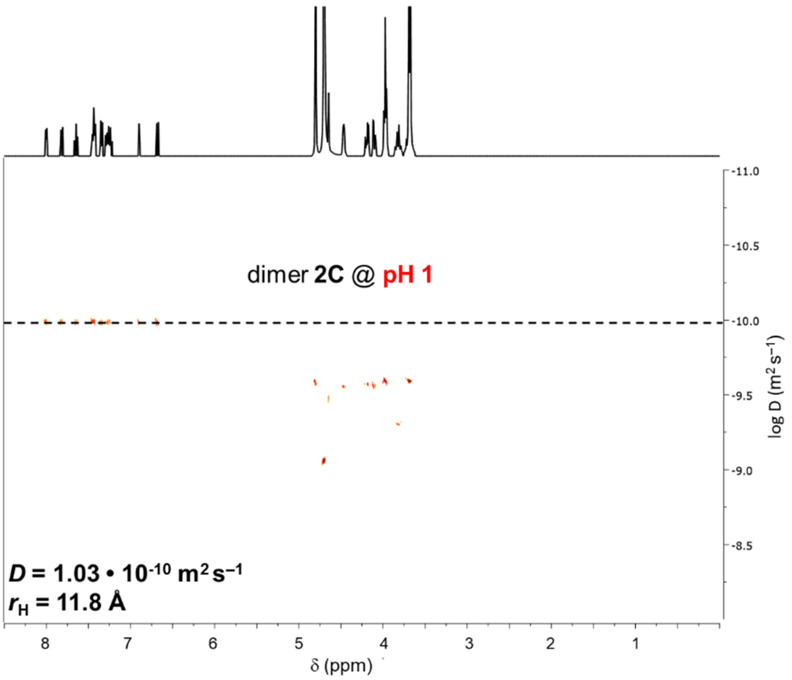

Figure S119. DOSY NMR spectra (600 MHz, TFE-d_3/D_2O 1:1, pD 1, 0.1 M CF_3SO_3D, 295 K) of dimer **2C.**

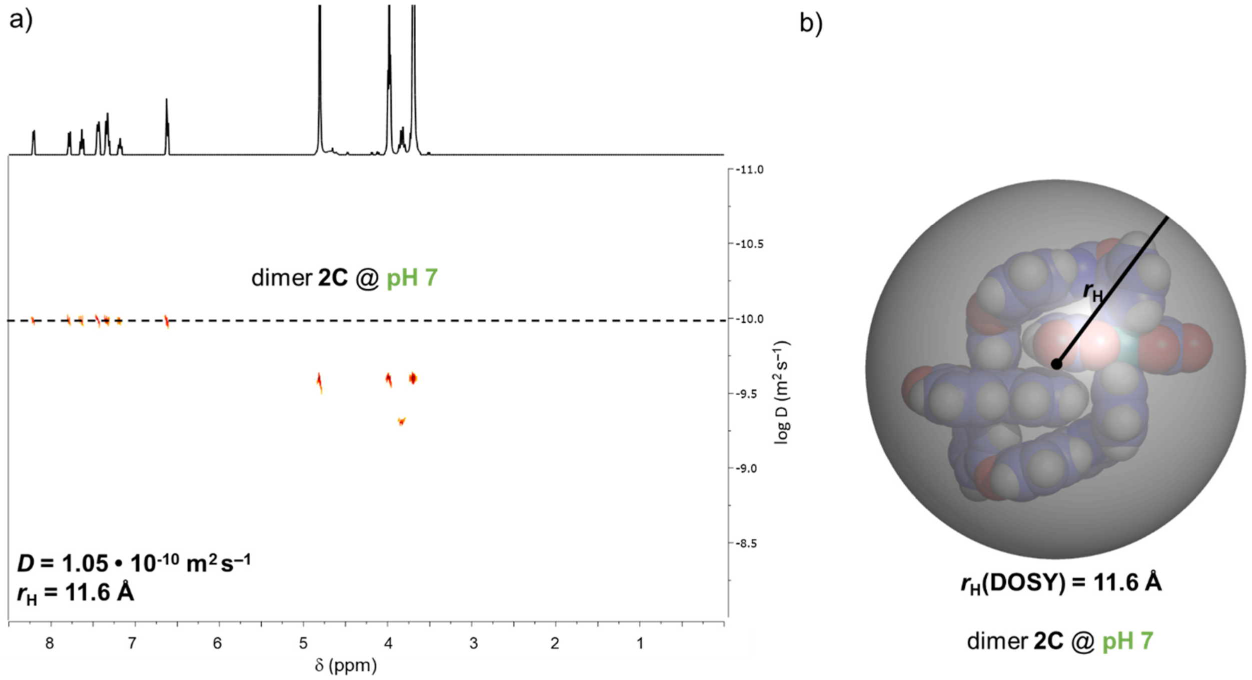

Figure S20. a) DOSY NMR spectra (600 MHz, TFE-d_3/D_2O 1:1, pD 7, 295 K) of dimer **2C**. b) Solvodynamic diameters are indicated as transparent spheres in the space-filling models of solid state structure of **2C.** The image b) has been prepared with PyMOL[S19]).

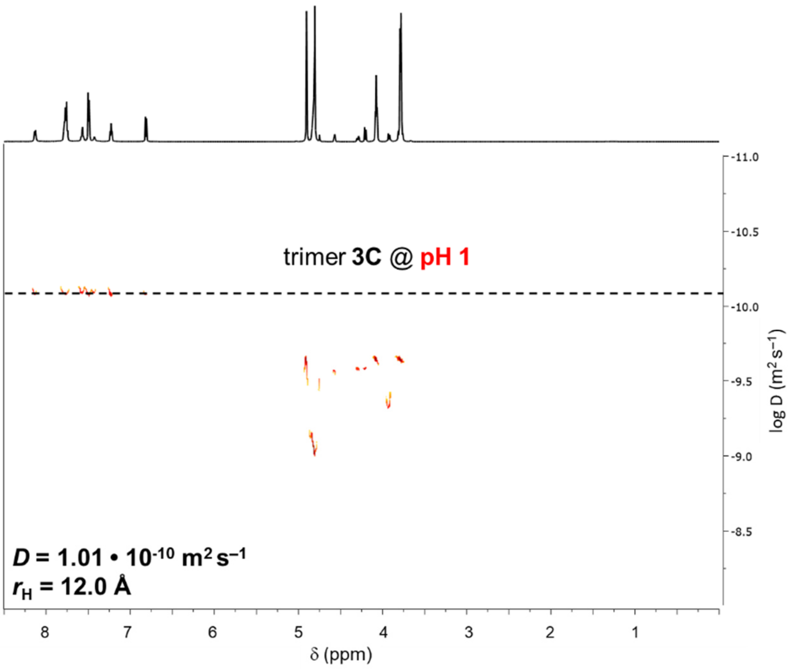

Figure S21. DOSY NMR spectra (600 MHz, TFE-d_3/D_2O 1:1, pD 1, 0.1 M CF_3SO_3D, 295 K) of trimer **3C.**

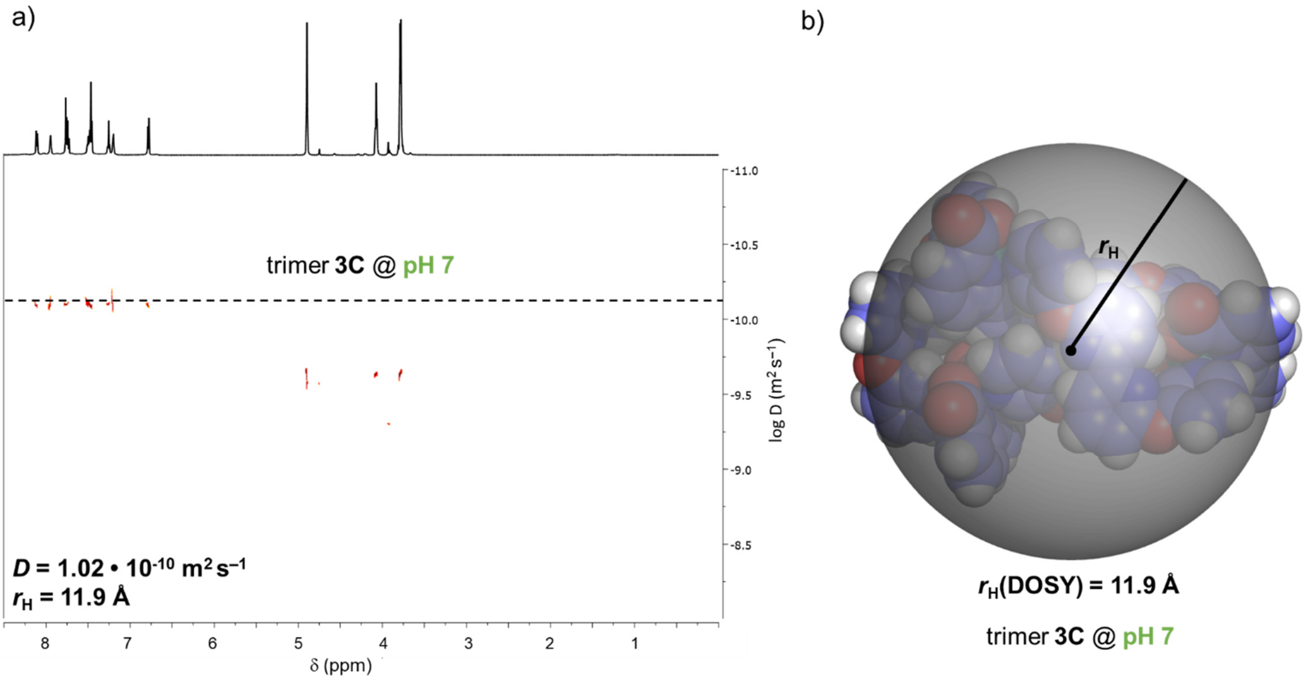

Figure S21. a) DOSY NMR spectra (600 MHz, TFE-d_3/D_2O 1:1, pD 7, 295 K) of trimer **3C**. b) Solvodynamic diameters are indicated as transparent spheres in the space-filling models of solid state structure of **3C.** The image b) has been prepared with PyMOL[S19]).

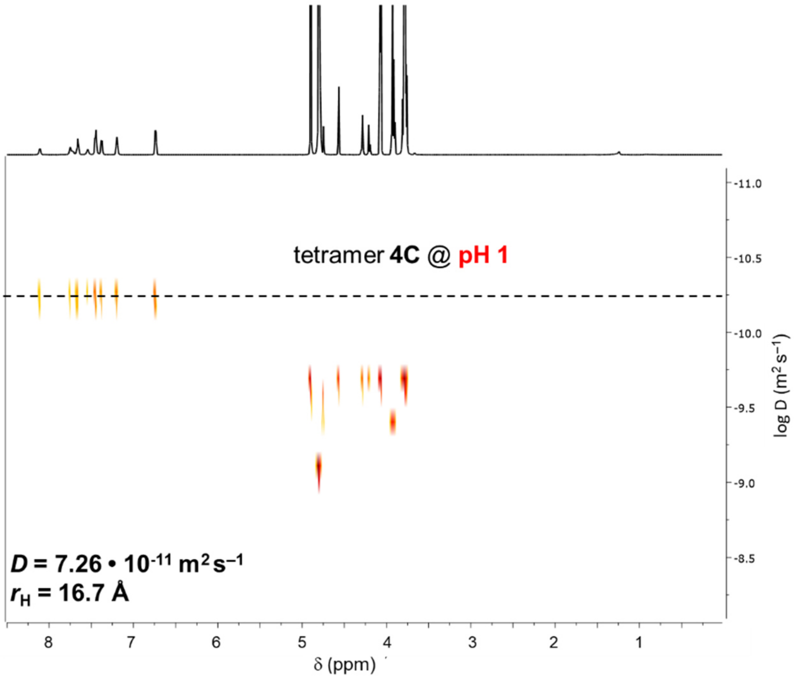

Figure S23. DOSY NMR spectra (600 MHz, TFE-d_3/D_2O 1:1, pD 1, 0.1 M CF_3SO_3D, 295 K) of tetramer **4C.**

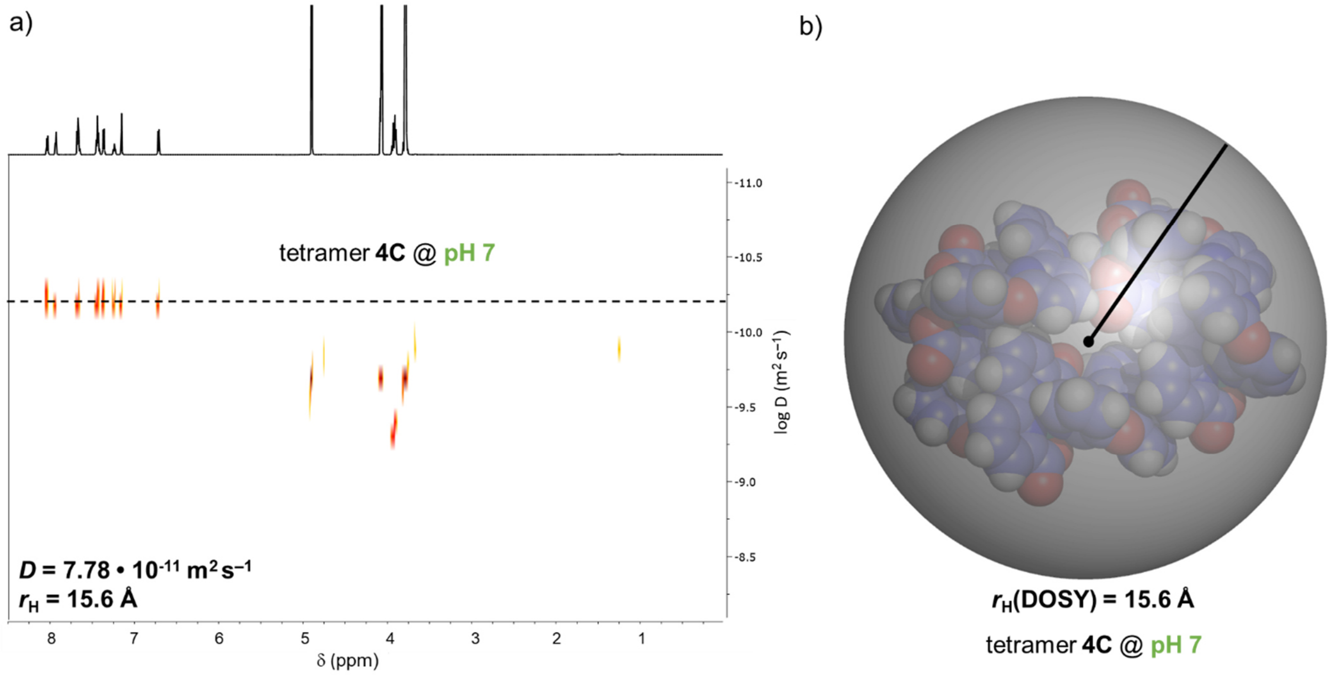

Figure S22. a) DOSY NMR spectra (600 MHz, TFE-d_3/D_2O 1:1, pD 7, 295 K) of tetramer **4C**. b) Solvodynamic diameters are indicated as transparent spheres in the space-filling models of solid state structure of **4C.** The image b) has been prepared with PyMOL[S19]).

Photocatalytic water oxidation

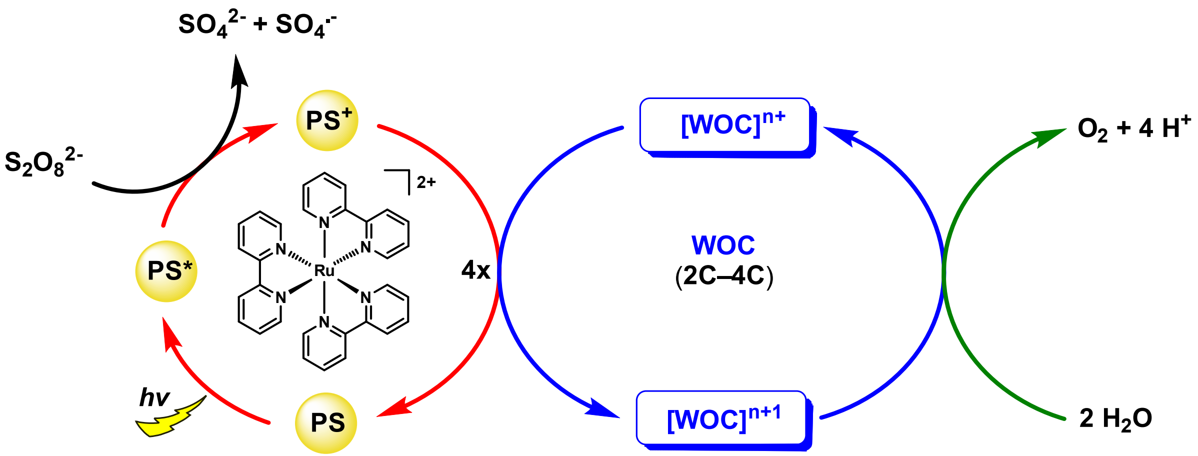

Figure S23. Schematic presentation of the photocatalytic water oxidation cycle in a three-component system containing $Na_2S_2O_8$ as sacrificial electron acceptor (SAE), $[Ru(bpy)_3]^{2+}$ as photosensitizer (PS) and complexes **2C**, **3C** or **4C** as water oxidation catalyst (WOC).

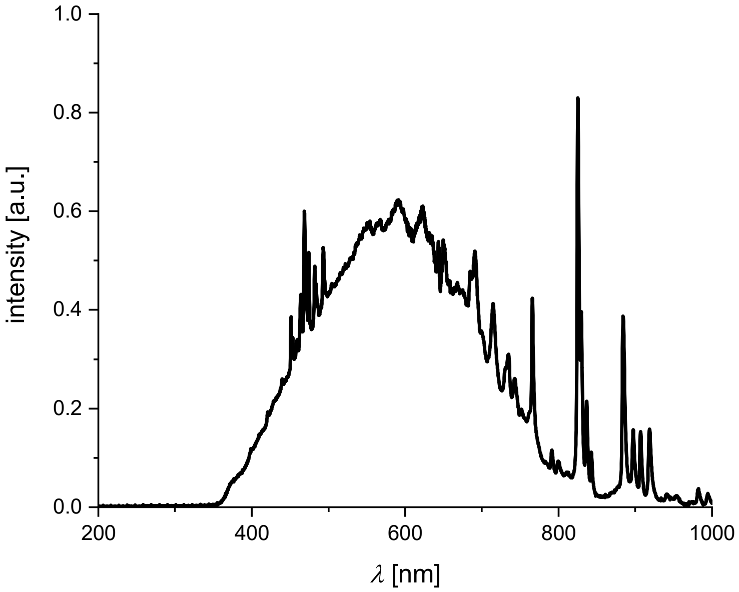

Figure S24. Emission spectrum of the xenon lamp used for photocatalytic water oxidation experiments.

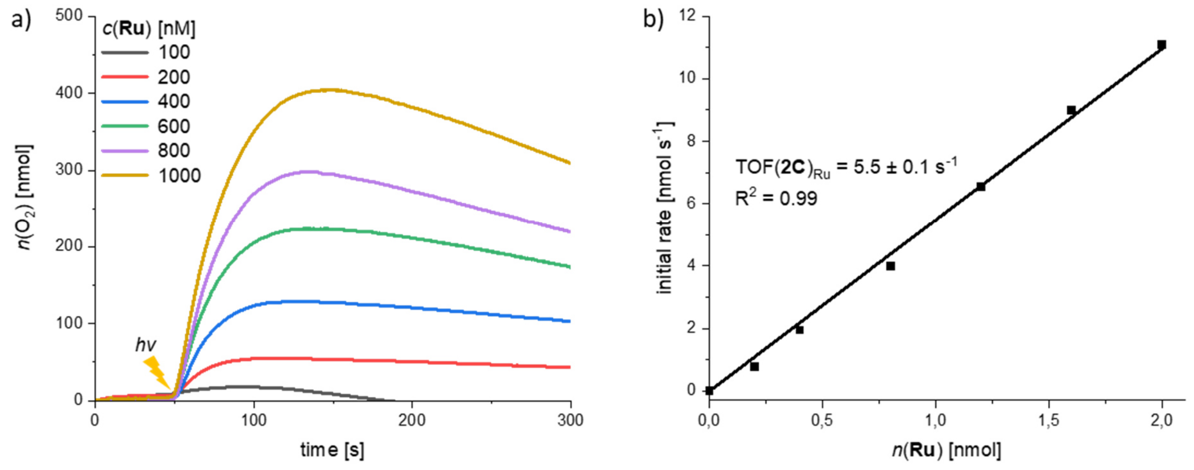

Figure S25. a) Concentration-dependent experiments with **2C** as WOC in CH_3CN/H_2O 4:6 (pH 7, 50 mM phosphate buffer), *c*(PS) = 1.5 mM, *c*($Na_2S_2O_8$) = 37 mM. The lighting symbol indicates the start of sample irradiation at *t* = 50 s. b) plot of the initial rates *vs*. the catalyst amount **per Ru unit** with linear regression for the determination of the TOF.

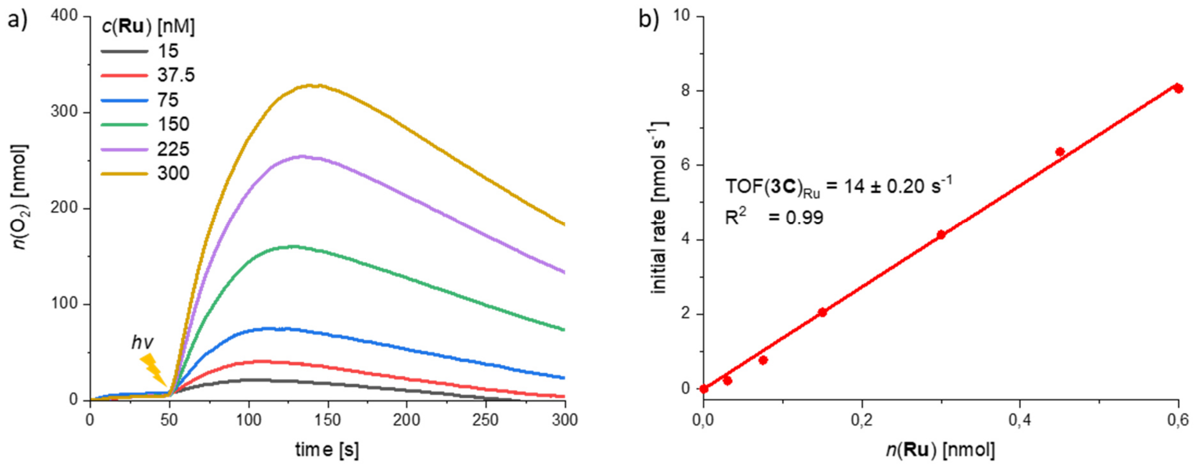

Figure S26. a) Concentration-dependent experiments with **3C** as WOC in CH_3CN/H_2O 4:6 (pH 7, 50 mM phosphate buffer), *c*(PS) = 1.5 mM, *c*($Na_2S_2O_8$) = 37 mM. The lighting symbol indicates the start of sample irradiation at *t* = 50 s. b) plot of the initial rates *vs*. the catalyst amount **per Ru unit** with linear regression for the determination of the TOF.

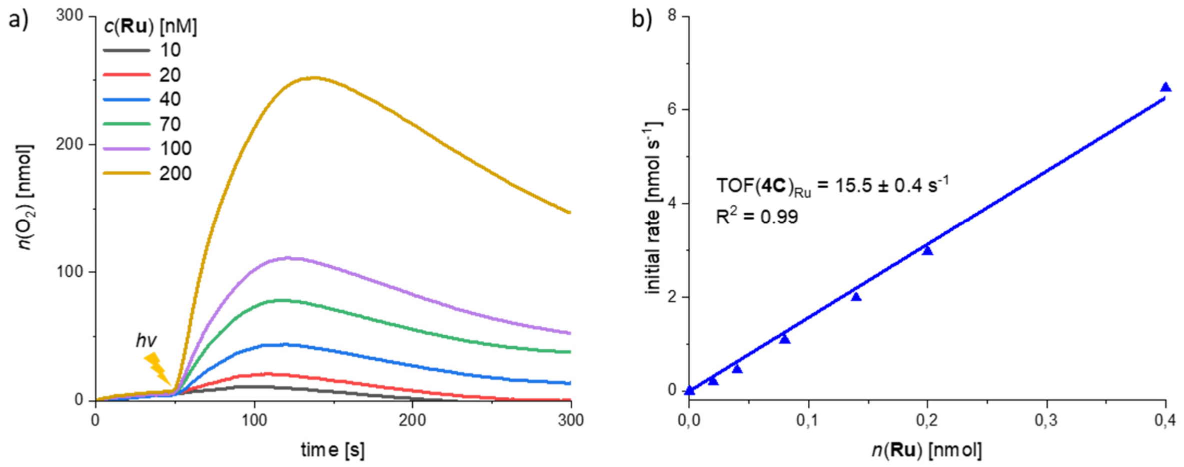

Figure S27. a) Concentration-dependent experiments with **4C** as WOC in CH_3CN/H_2O 4:6 (pH 7, 50 mM phosphate buffer), *c*(PS) = 1.5 mM, *c*($Na_2S_2O_8$) = 37 mM. The lighting symbol indicates the start of sample irradiation at *t* = 50 s. b) plot of the initial rates *vs.* the catalyst amount **per Ru unit** with linear regression for the determination of the TOF.

Chemical water oxidation

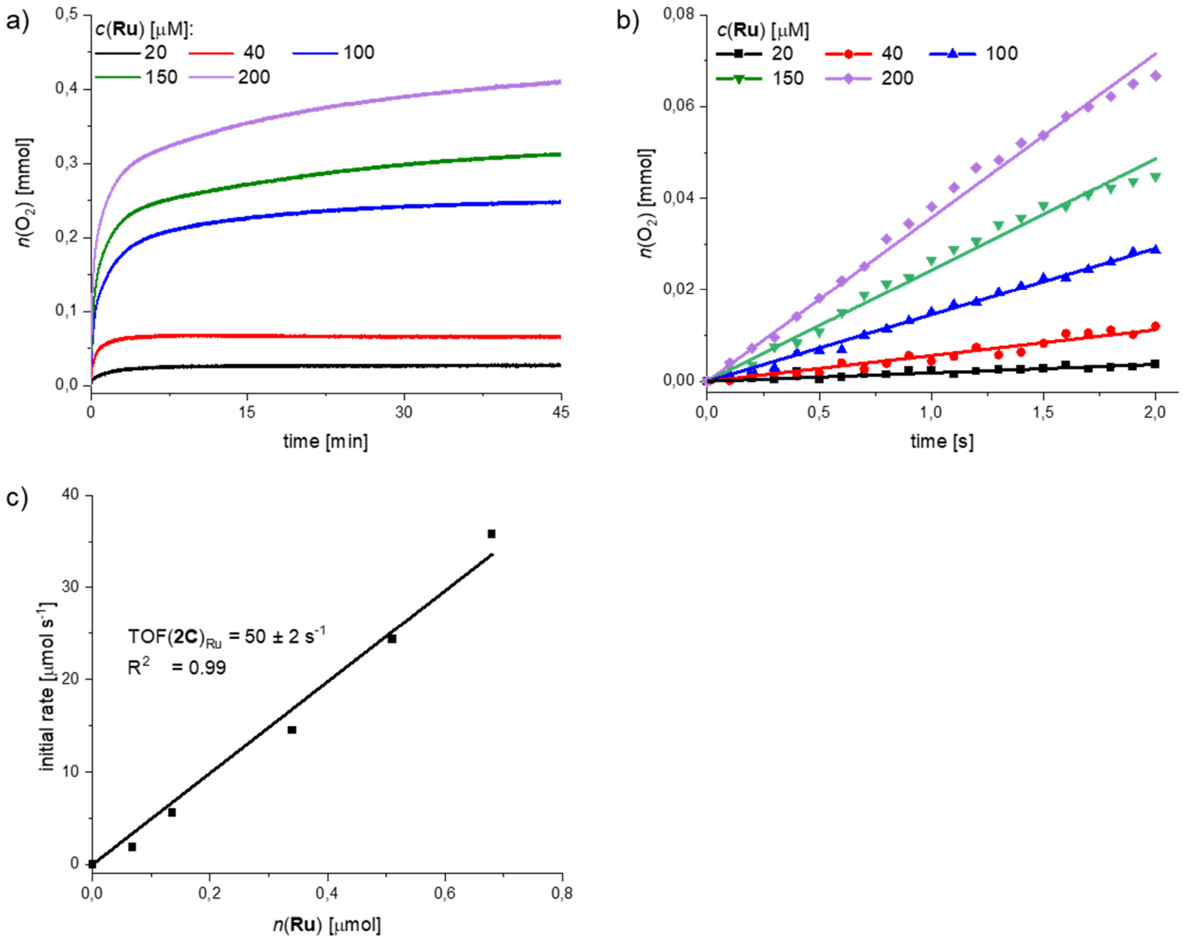

Figure S28. a) Oxygen evolution curves of **2C/Ru unit** at variable concentrations in CH_3CN/H_2O 4:6 (pH 1, triflic acid), *c*(CAN) = 0.6 M. b) linear regression of oxygen evolution for **2C/Ru unit** in the first 2 s of catalysis. c) plot of the initial rate vs. the catalyst amount with linear regression for the determination of the TOF_{Ru}.

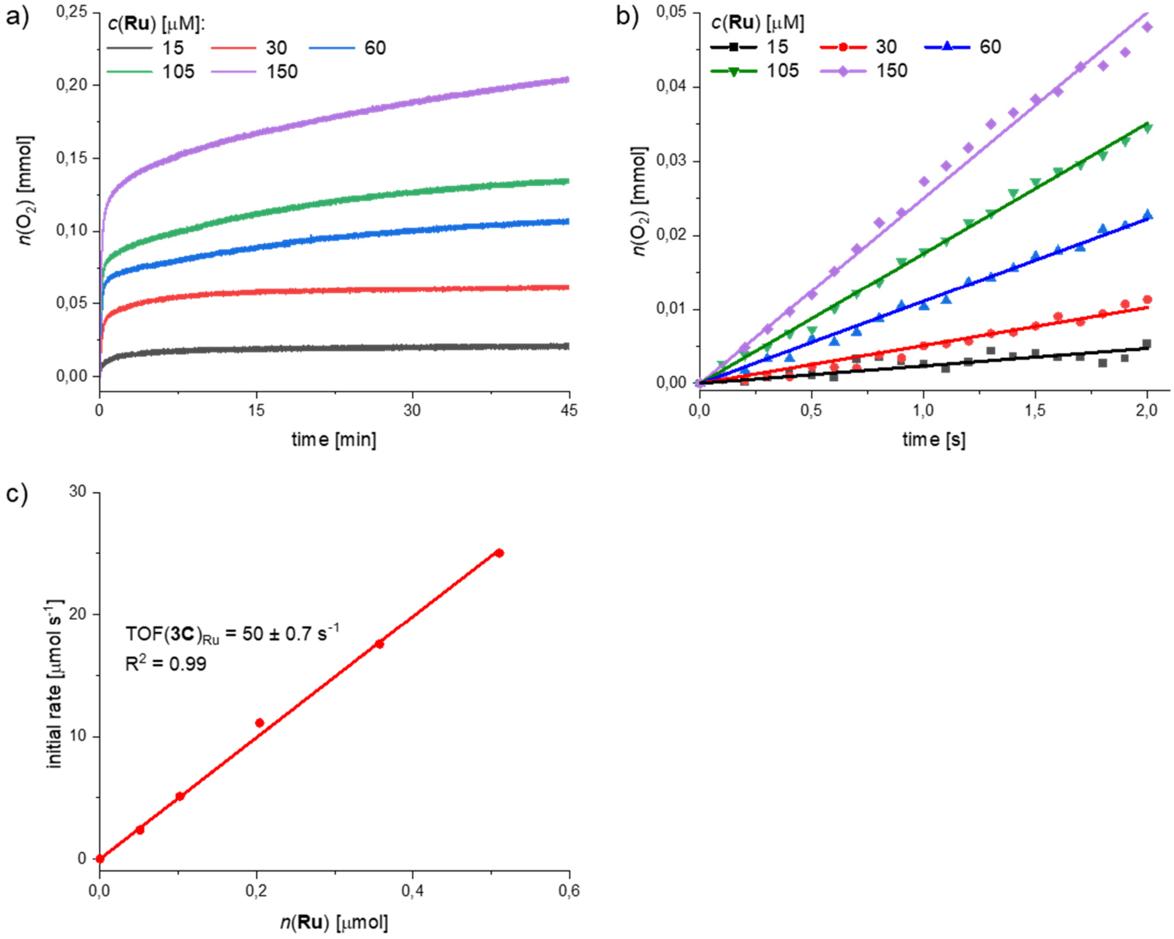

Figure S29. a) Oxygen evolution curves of **3C/Ru unit** at variable concentrations in CH_3CN/H_2O 4:6 (pH 1, triflic acid), *c*(CAN) = 0.6 M. b) linear regression of oxygen evolution for **3C/Ru unit** in the first 2 s of catalysis. c) plot of the initial rate vs. the catalyst amount with linear regression for the determination of the TOF_{Ru}.

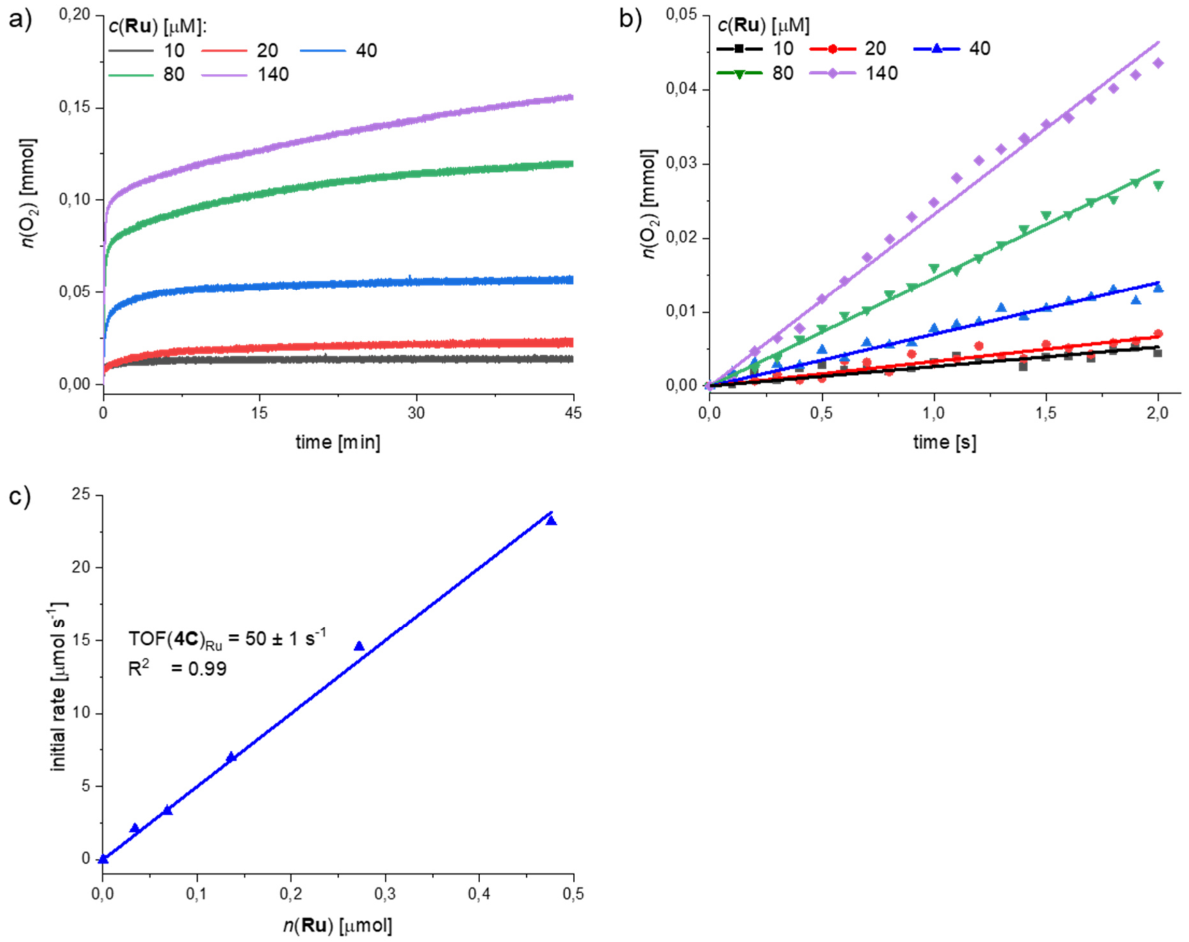

Figure S30. a) Oxygen evolution curves of **4C**/**Ru unit** at variable concentrations in CH_3CN/H_2O 4:6 (pH 1, triflic acid), *c*(CAN) = 0.6 M. b) linear regression of oxygen evolution for **4C**/**Ru unit** in the first 2 s of catalysis. c) plot of the initial rate vs. the catalyst amount with linear regression for the determination of the TOF_{Ru}.

Table S6. Catalytic activities and reported water oxidation mechanism for selected Ru WOCs in chemical and photocatalytic water oxidation.

Catalyst	Chemical water oxidation (CAN)			Photocatalytic water oxidation (PS/SEA)			Water Oxidation Mechanism
	TOF_{max} [s^{-1}]	TOF_{max}/ Ru [s^{-1}]	TON	TOF_{max} [s^{-1}]	TOF_{max}/ Ru [s^{-1}]	TON	
**2C**[a]	99	49.5	1200	11	5.5	400	WNA
**3C**[b]	149	49.5	1400	42	14	1500	WNA
**4C**[c]	200	50	1600	62	15.5	2200	WNA
**1**[d] [S20,S21]	41	41	2000	0.35	0.35	10	I2M
**2**[e] [S22,S23]	303	303	8360	0.24	0.24	140	I2M
**3**[f] [S24]	inactive	/	inactive	50	50	1050	WNA
**4**[g] [S25,S26]	n.d.	n.d	20800	n.d.	n.d.	640	I2M (Intra)
**5**[h] [S27,S28]	0.068	0.034	211	11	5.5	5300	WNA
**6**[i] [S18]	19	9.5	820	15.5	7.8	460	WNA
**OEG-MC2**[j] [S29]	12	6	200	1.1	0.6	36	WNA
**OEG-MC3**[k][S29]	26	8.7	2200	10	3.3	400	WNA
**MC3**[l] [S15,S17]	136	45	5300	11	3.7	430	WNA
**OEG-MC4**[m,S29]	42	10.5	2870	23	5.8	500	WNA

Experimental conditions:

[**a**] Chemical WO: 40:60 MeCN/H_2O (pH 1, triflic acid), *c*(CAN) = 0.6 M, *c*(**2C**) = 10–100 μM; Photocatalytic WO: 40:60 MeCN/H_2O (pH 7, 50 mM phosphate buffer), *c*(([Ru(bpy)$_3$]$^{2+}$) = 1.5 mM, *c*($Na_2S_2O_8$) = 37 mM, *c*(**2C**) = 50–500 nM.

[**b**] Chemical WO: 40:60 MeCN/H_2O (pH 1, triflic acid), *c*(CAN) = 0.6 M, *c*(**3C**) = 5-50 μM; Photocatalytic WO: 40:60 MeCN/H_2O (pH 7, 50 mM phosphate buffer), *c*(([Ru(bpy)$_3$]$^{2+}$) = 1.5 mM, *c*($Na_2S_2O_8$) = 37 mM, *c*(**3C**) = 5–100 nM.

[**c**] Chemical WO: 40:60 MeCN/H_2O (pH 1, triflic acid), *c*(CAN) = 0.6 M, *c*(**4C**) = 2.5–35 μM; Photocatalytic WO: 40:60 MeCN/H_2O (pH 7, 50 mM phosphate buffer), *c*(([Ru(bpy)$_3$]$^{2+}$) = 1.5 mM, *c*($Na_2S_2O_8$) = 37 mM, *c*(**4C**) = 2.5–50 nM.

[**d**] Chemical WO: H_2O (pH 1, triflic acid), *c*(CAN) = 0.4 mM, *c*(**1**) = 12-216 μM;[S20] Photocatalytic WO: H_2O (pH 7.2, phosphate buffer), *c*([Ru(bpy)$_3$]$^{2+}$) = 1 mM, *c*($Na_2S_2O_8$) = 10 mM, *c*(**1**) = 9.5 μM.[S21]

[**e**] Chemical WO: H_2O (pH 1, triflic acid), *c*(CAN) = 0.5 mM, *c*(**2**) = 114-216 μM;[S22] Photocatalytic WO: H_2O (pH 1, perchlorid acid), *c*([Ru(5-CF$_3$-bpy)$_3$]$^{2+}$) = 0.2 mM, *c*($Na_2S_2O_8$) = 1 mM, *c*(**2**) = 20 μM.[S23]

[**f**] Chemical WO: inactive. Photocatalytic WO: H_2O (pH 7, phosphate buffer), c([Ru(4,4'-COOEt-bpy)$_2$bpy]$^{2+}$) = 0.2 mM, c($Na_2S_2O_8$) = 10 mM, c(**3**) = 1–16 μM.[S24]

[**g**] Chemical WO: H_2O (pH 1, triflic acid), *c*(CAN) = 5 mM, *c*(**5**) = 50 nM;[S25] Photocatalytic WO: 6:4 MeCN/H_2O (pH 6.8, phosphate buffer), *c*([Ru(bpy)$_3$]$^{2+}$) = 1 mM, *c*($Na_2S_2O_8$) = 45 mM, *c*(**5**) = 2 μM.[S26]

[**h**] Chemical WO: H_2O (pH 1, triflic acid), *c*(CAN) = 0.1 M, *c*(**5**) = 0.1-1 mM;[S27] Photocatalytic WO: H_2O (pH 7, phosphate buffer), *c*([Ru(4,4'-COOEt-bpy)$_2$bpy]$^{2+}$) = 0.2 mM, *c*($Na_2S_2O_8$) = 20 mM, *c*(**5**) = 0.2 μM.[S28]

[**i**] Chemical WO: 40:60 MeCN/H_2O (pH 1, triflic acid), *c*(CAN) = 0.6 M, *c*(**6**) = 50-300 μM; Photocatalytic WO: 40:60 MeCN/H_2O (pH 7, phosphate buffer), *c*(([Ru(bpy)$_3$]$^{2+}$) = 1.5 mM, *c*($Na_2S_2O_8$) = 37 mM, *c*(**6**) = 12-200 nM. [S18]

[**j**] Chemical WO: 1:1 MeCN/H_2O (pH 1, triflic acid), *c*(CAN) = 0.6 M, *c*(**OEG-MC2**) = 96-494 μM; Photocatalytic WO: 1:1 MeCN/H_2O (pH 7, 50 mM phosphate buffer), *c*(([Ru(bpy)$_3$]$^{2+}$) = 1.5 mM, *c*($Na_2S_2O_8$) = 37 mM, *c*(**OEG-MC2**) = 0.05-5.8 μM.[S29]

[**k**] Chemical WO: 1:1 MeCN/H_2O (pH 1, triflic acid), *c*(CAN) = 0.6 M, *c*(**OEG-MC3**) = 50-250 μM; Photocatalytic WO: 1:1 MeCN/H_2O (pH 7, 50 mM phosphate buffer), *c*(([Ru(bpy)$_3$]$^{2+}$) = 1.5 mM, *c*($Na_2S_2O_8$) = 37 mM, *c*(**OEG-MC3**) = 60-2050 nM.[S29]

[**l**] Chemical WO: 1:1 MeCN/H_2O (pH 1, triflic acid), *c*(CAN) = 0.6 M, *c*(**MC3**) = 6-94 μM;[S15] Photocatalytic WO: 1:1 MeCN/H_2O (pH 7, phosphate buffer), *c*(([Ru(bpy)$_3$]$^{2+}$) = 1.5 mM, *c*($Na_2S_2O_8$) = 37 mM, *c*(**MC3**) = 60-880 nM.[S17]

[**m**] Chemical WO: 1:1 MeCN/H_2O (pH 1, triflic acid), *c*(CAN) = 0.6 M, *c*(**OEG-MC4**) = 10-60 μM; Photocatalytic WO: 1:1 MeCN/H_2O (pH 7, 50 mM phosphate buffer), *c*(([Ru(bpy)$_3$]$^{2+}$) = 1.5 mM, *c*($Na_2S_2O_8$) = 37 mM, *c*(**OEG-MC4**) = 6-600 nM.[S29]

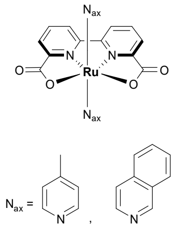

1 2

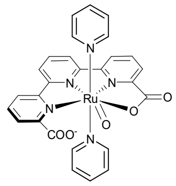

3

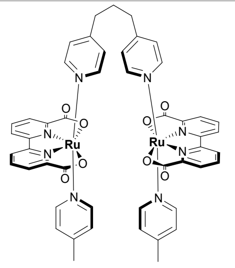

4

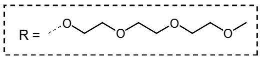

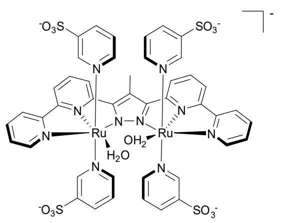

5

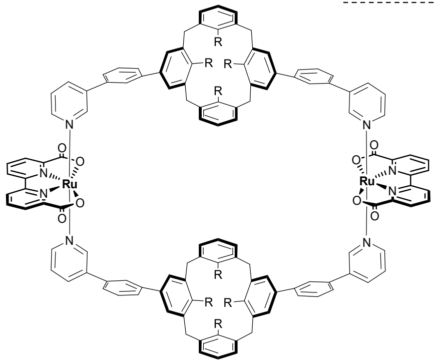

6

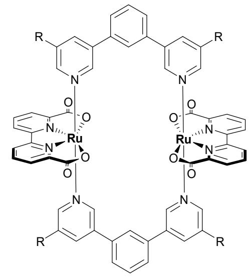

OEG-MC2

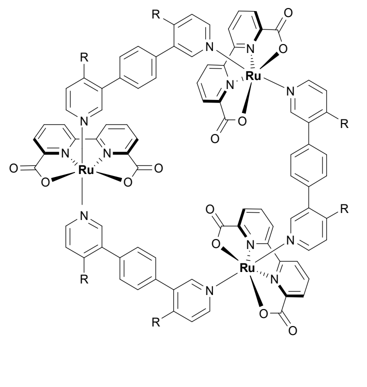

OEG-MC3 (R = OEG)
MC3 (R = H)

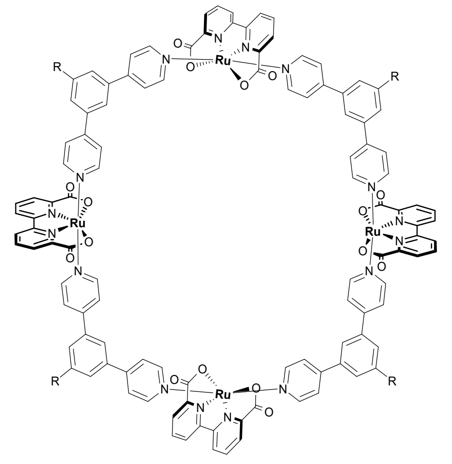

OEG-MC4

Figure S33. Chemical structures of selected mono- and multinuclear Ru WOCs.

Stability tests before chemical water oxidation

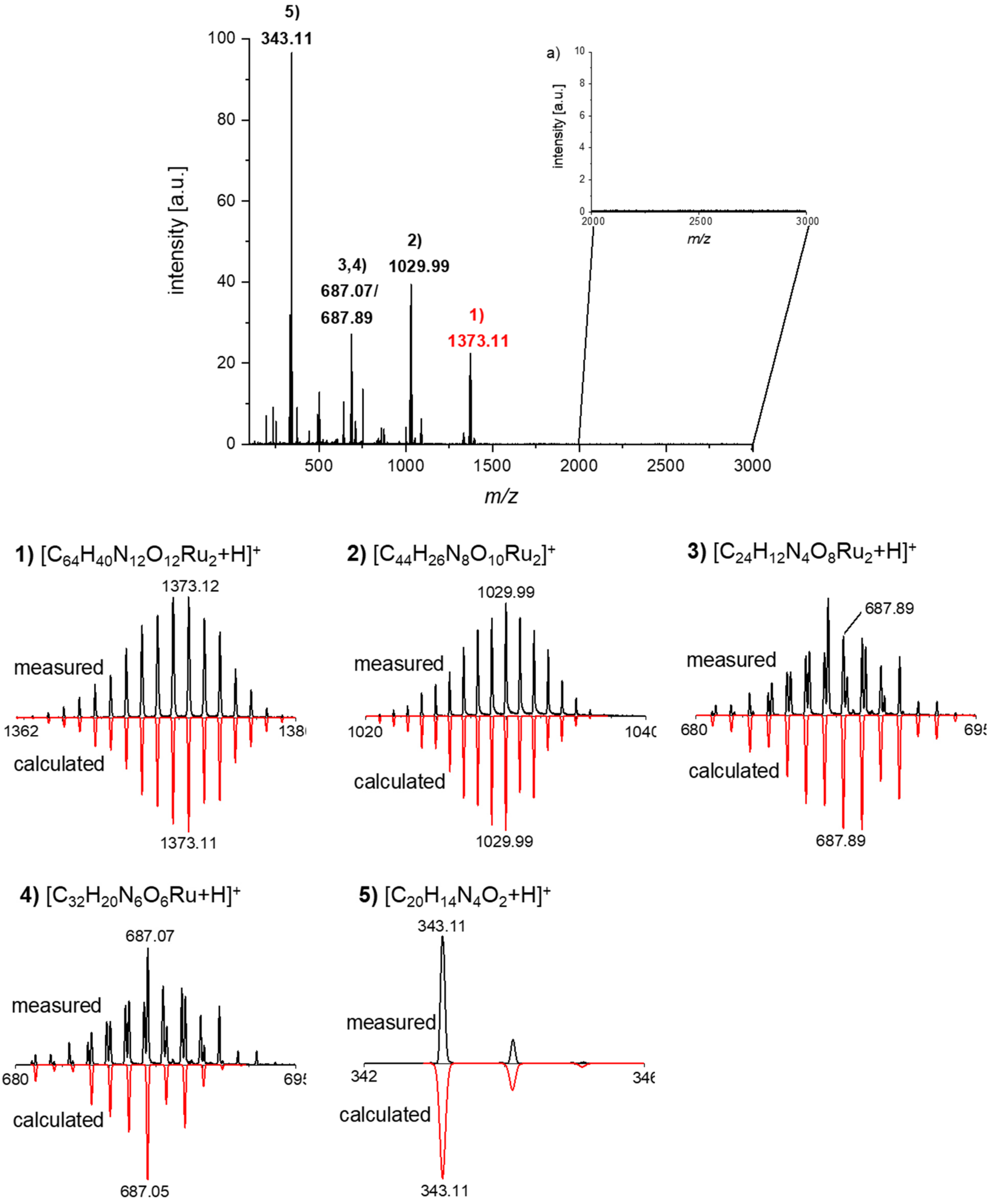

Figure S34. HR MALDI mass spectrum (CH_2Cl_2/CH_3OH 1:1, positive mode, DCTB) of **2C** before water oxidation catalysis The inset a) shows the amplified region between 2000–3000 m/z and insets 1–5) show the measured and calculated isotopic distribution of the most prominent fragmentation peaks.

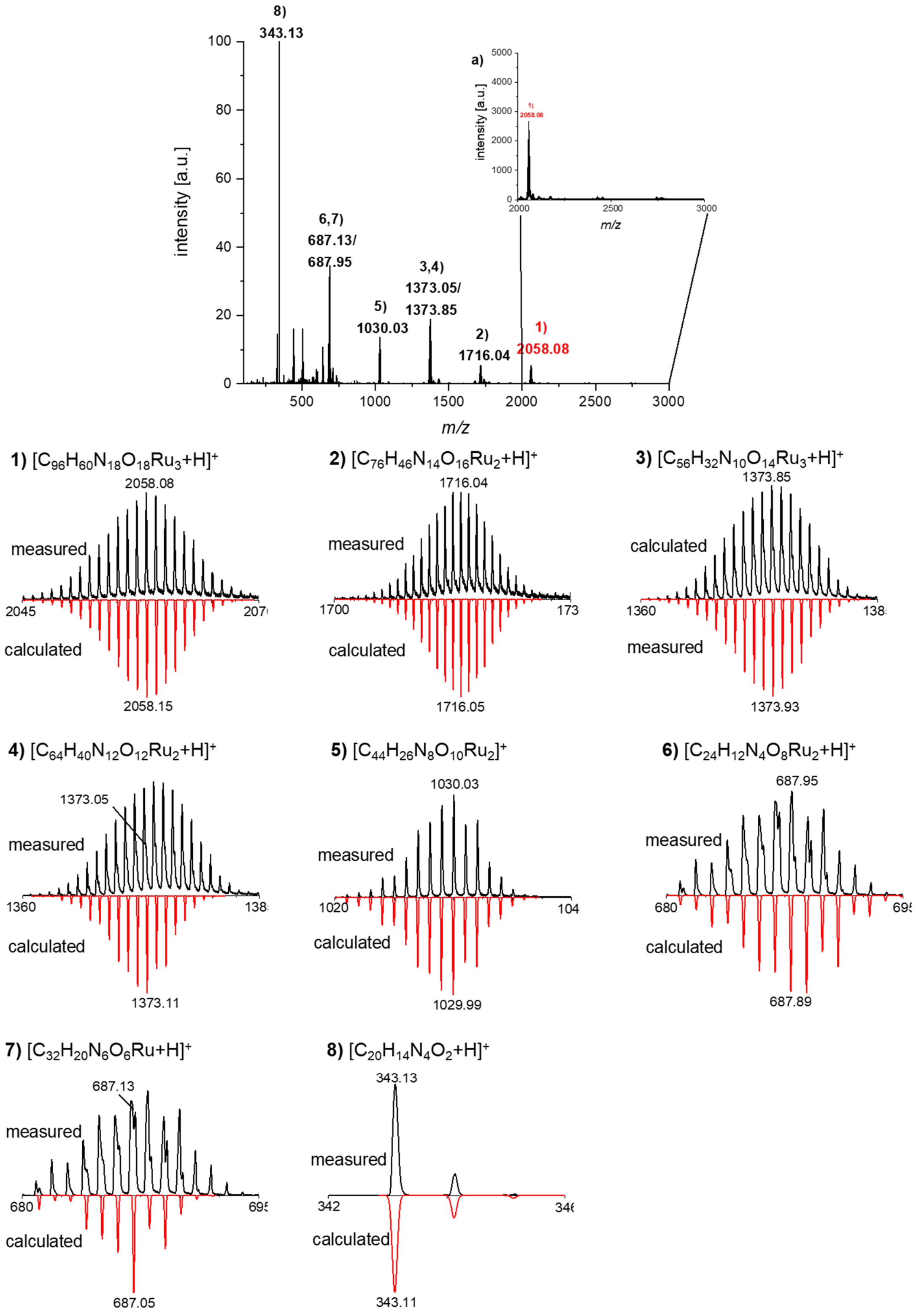

Figure S35. HR MALDI mass spectrum (CH_2Cl_2/CH_3OH 1:1, positive mode, DCTB) of **3C** before water oxidation catalysis The inset a) shows the amplified region between 2000–3000 m/z and insets 1–8) show the measured and calculated isotopic distribution of the most prominent fragmentation peaks.

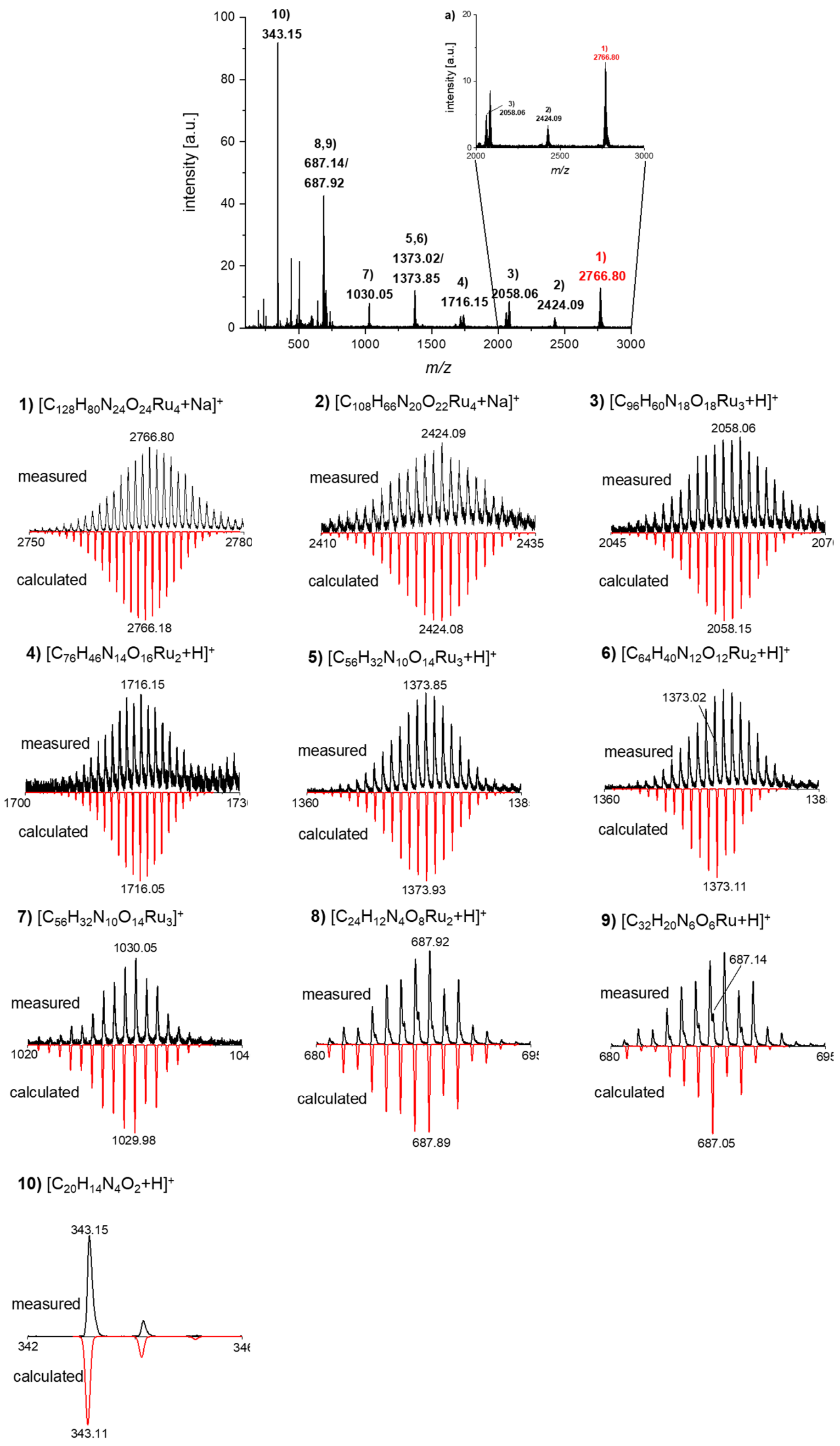

Figure S36. HR MALDI mass spectrum (CH_2Cl_2/CH_3OH 1:1, positive mode, DCTB) of **4C** before water oxidation catalysis The inset a) shows the amplified region between 2000–3000 m/z and insets 1–10) show the measured and calculated isotopic distribution of the most prominent fragmentation peaks.

Stability tests under conditions of chemical water oxidation

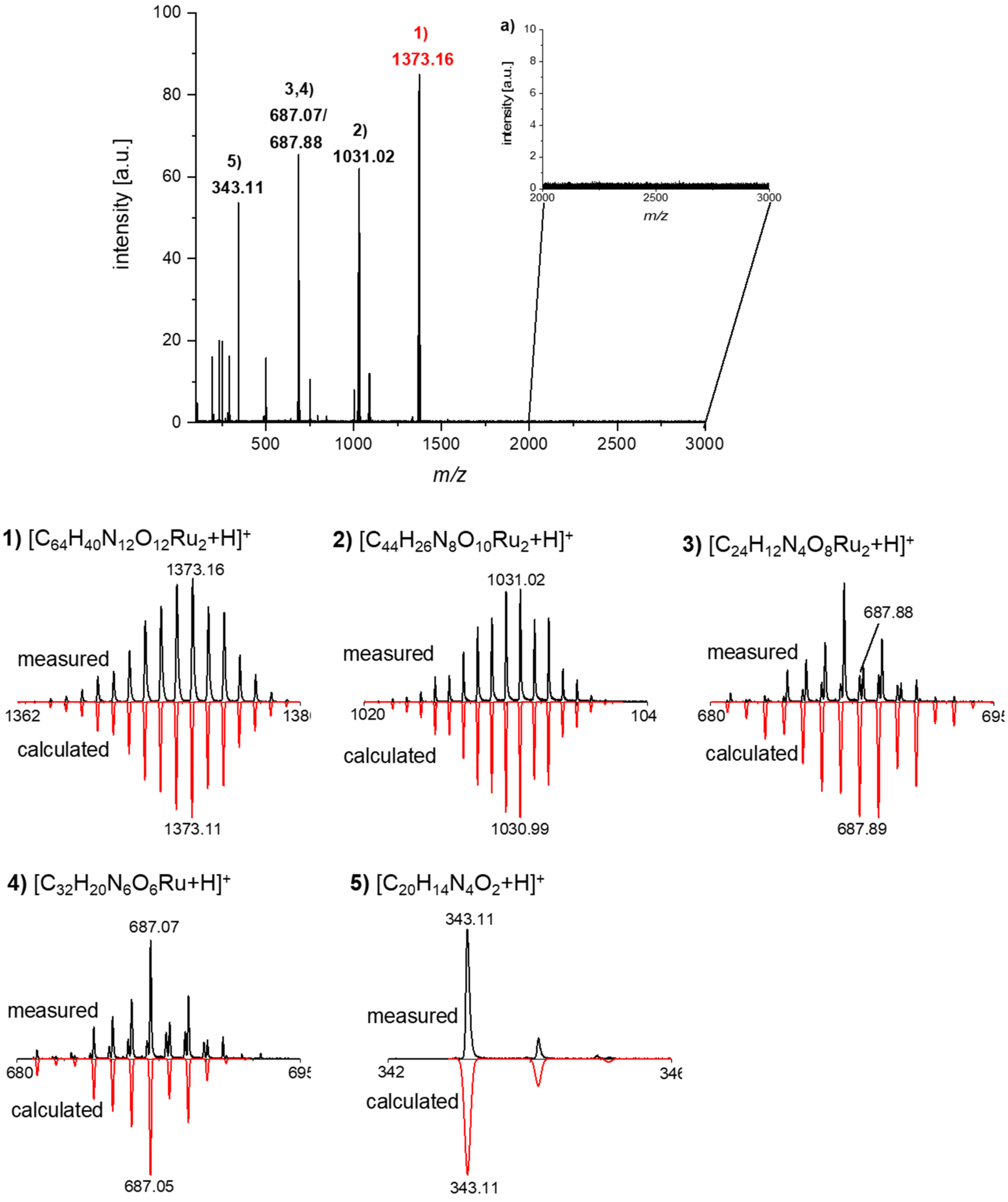

Figure S37. HR MALDI mass spectrum (CH_2Cl_2/CH_3OH 1:1, positive mode, DCTB) of **2C** after water oxidation catalysis (~40 catalytic cycles) with cerium ammonium nitrate (CAN) as oxidant in 4:6 CH_3CN/H_2O (pH 1, triflic acid). Before the measurement, the sample was reduced with ascorbic acid. The inset a) shows the amplified region between 2000–3000 m/z and insets 1–5) show the measured and calculated isotopic distribution of the most prominent fragmentation peaks.

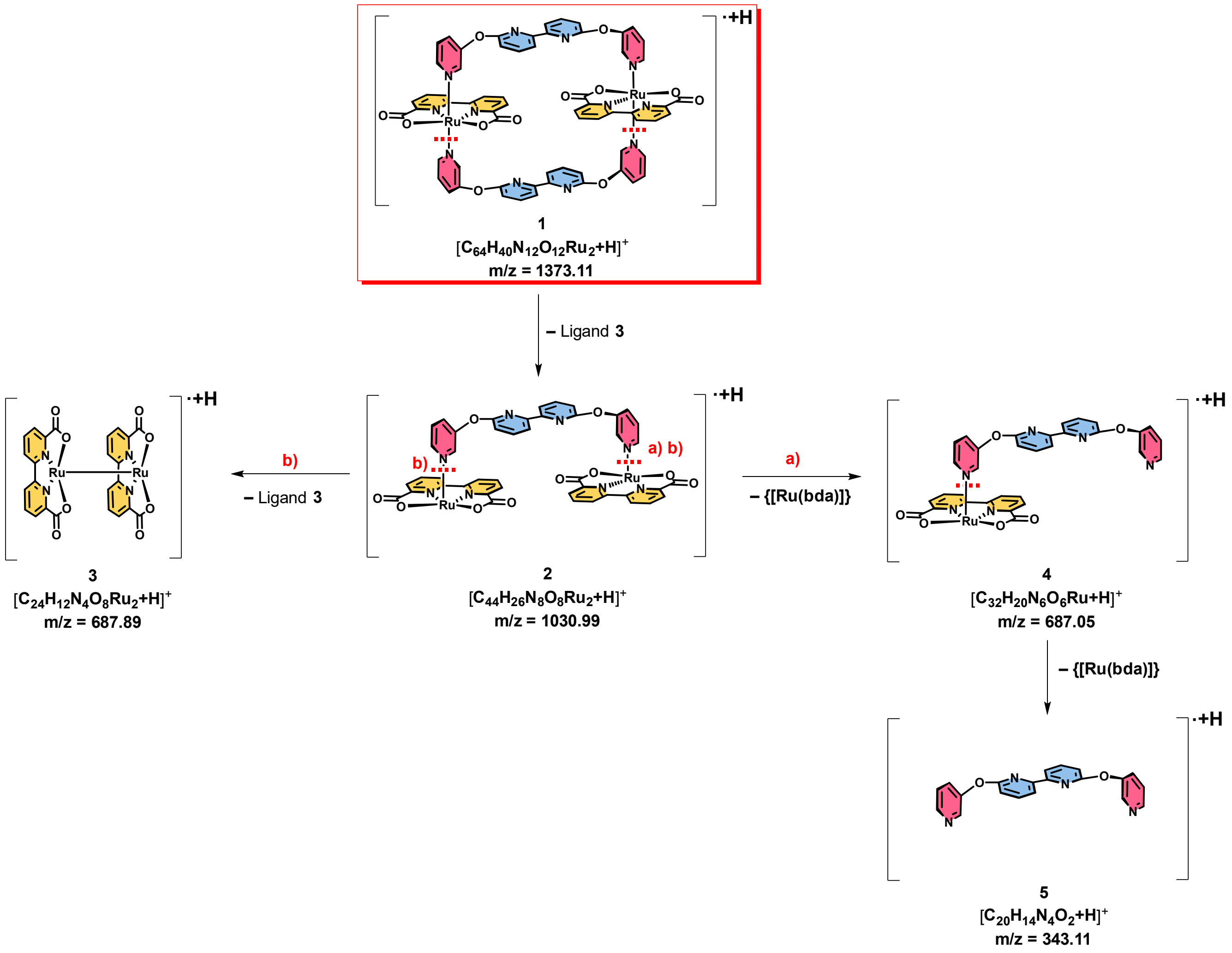

Figure S38. Proposed fragmentation pathway for dimer **2C** after chemical water oxidation catalysis.

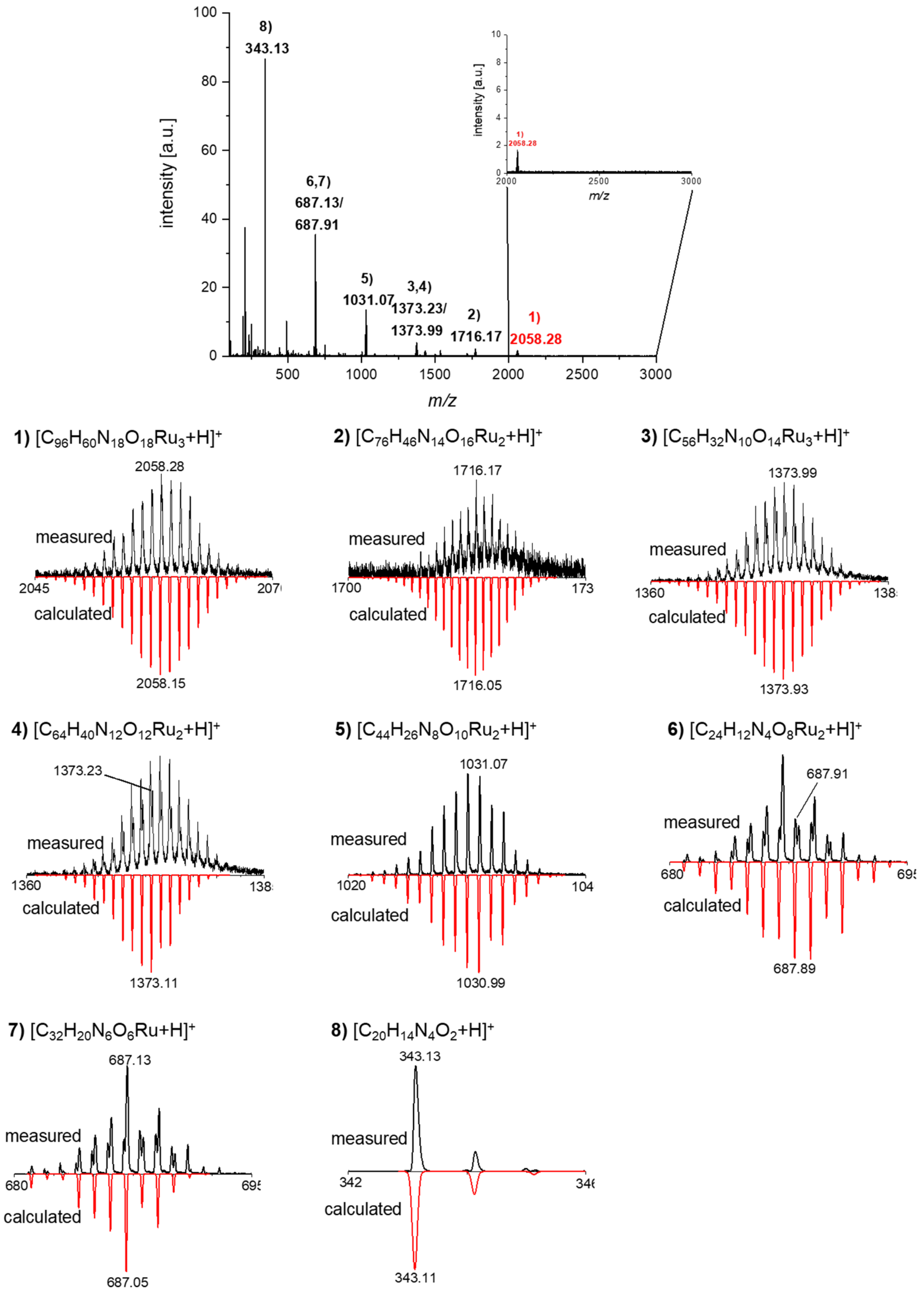

Figure S39. HR MALDI mass spectrum (CH_2Cl_2/CH_3OH 1:1, positive mode, DCTB) of **3C** after water oxidation catalysis (~40 catalytic cycles) with cerium ammonium nitrate (CAN) as oxidant in 4:6 CH_3CN/H_2O (pH 1, triflic acid). Before the measurement, the sample was reduced with ascorbic acid. The inset a) shows the amplified region between 2000–3000 m/z and insets 1–8) show the measured and calculated isotopic distribution of the most prominent fragmentation peaks.

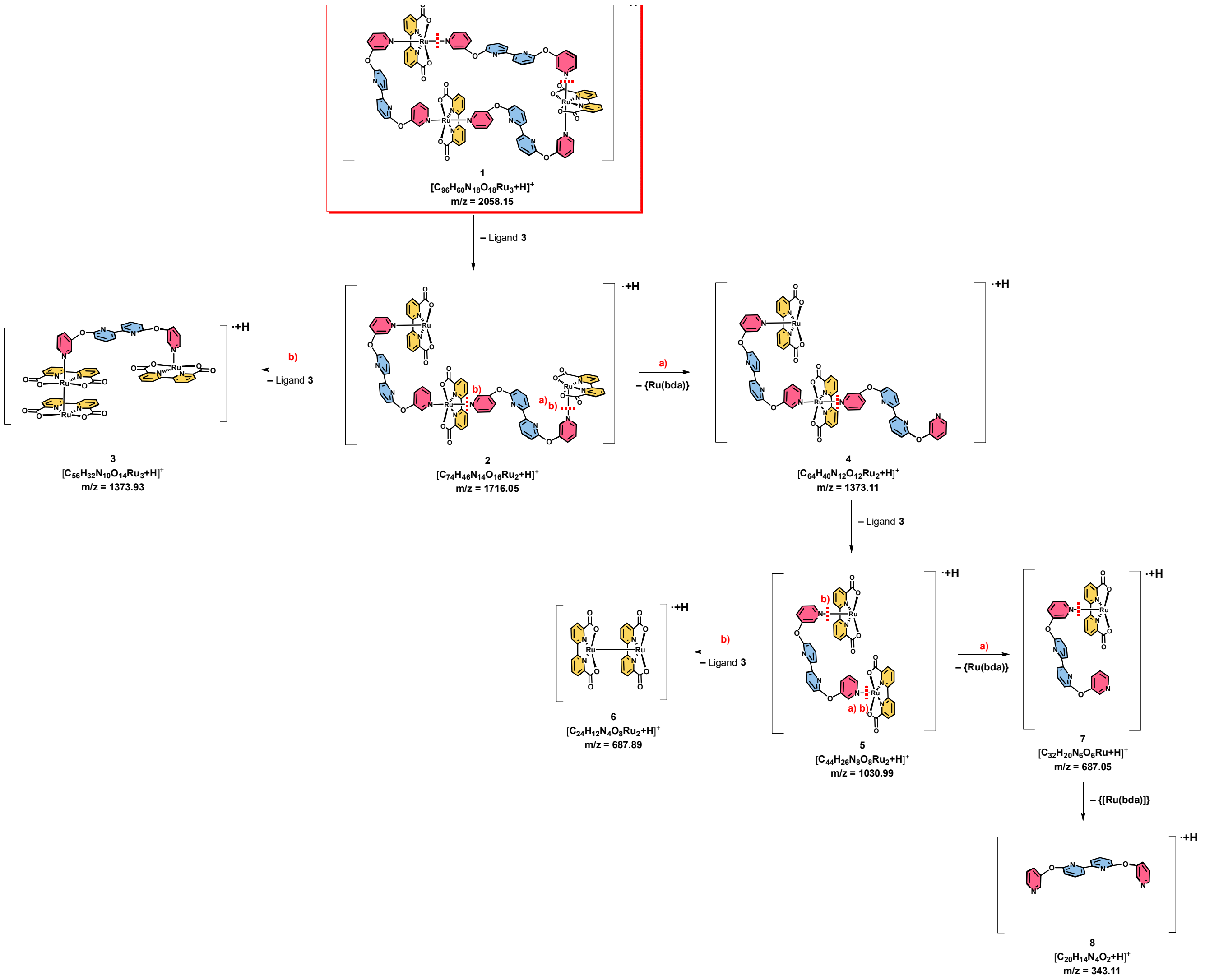

Figure S40. Proposed fragmentation pathway for trimer **3C** after chemical water oxidation catalysis.

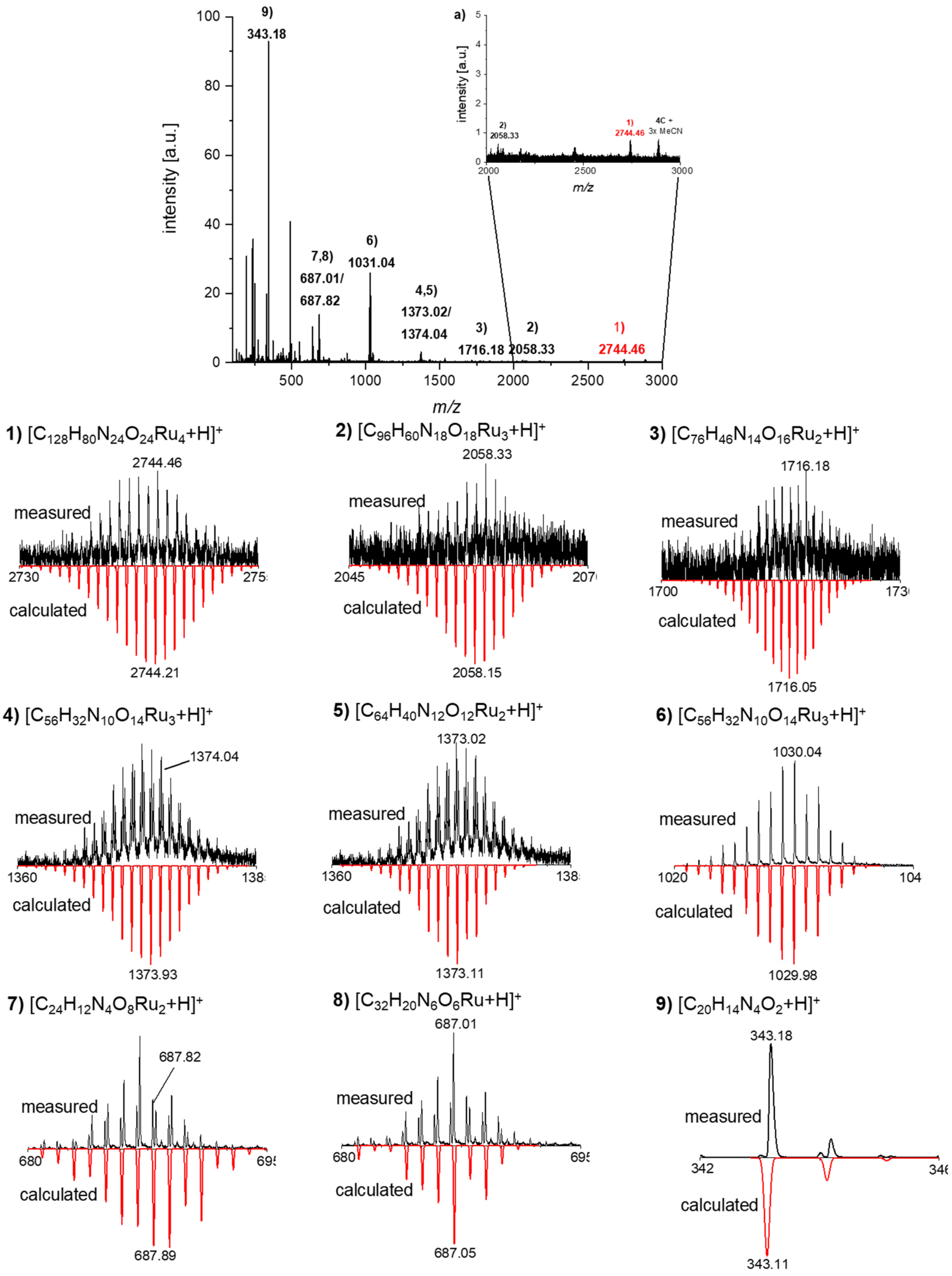

Figure S41. HR MALDI mass spectrum (CH_2Cl_2/CH_3OH 1:1, positive mode, DCTB) of **4C** after water oxidation catalysis (~40 catalytic cycles) with cerium ammonium nitrate (CAN) as oxidant in 4:6 CH_3CN/H_2O (pH 1, triflic acid). Before the measurement, the sample was reduced with ascorbic acid. The inset a) shows the amplified region between 2000–3000 m/z and insets 1–9) show the measured and calculated isotopic distribution of the most prominent fragmentation peaks.

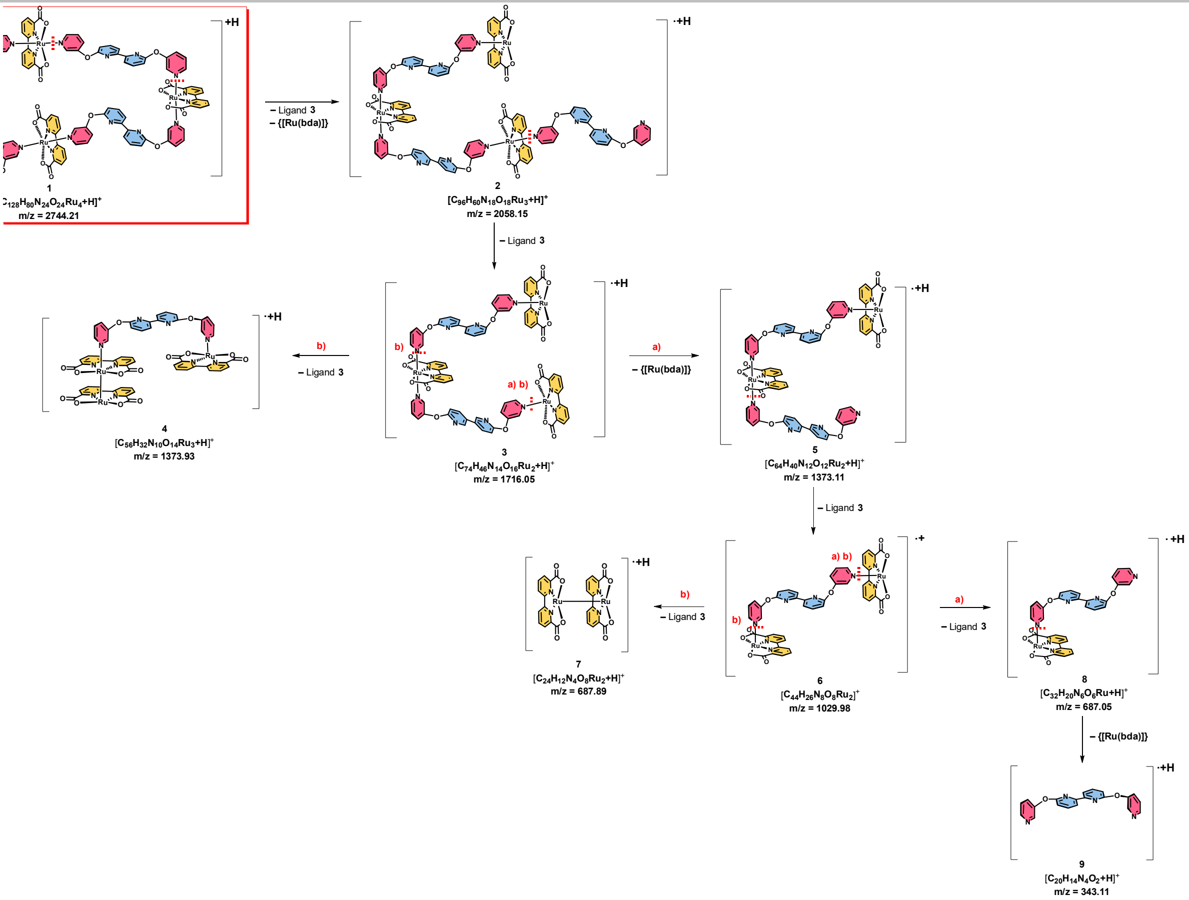

Figure S42. Proposed fragmentation pathway for tetramer **4C** after chemical water oxidation catalysis.

Kinetic Isotope Effect

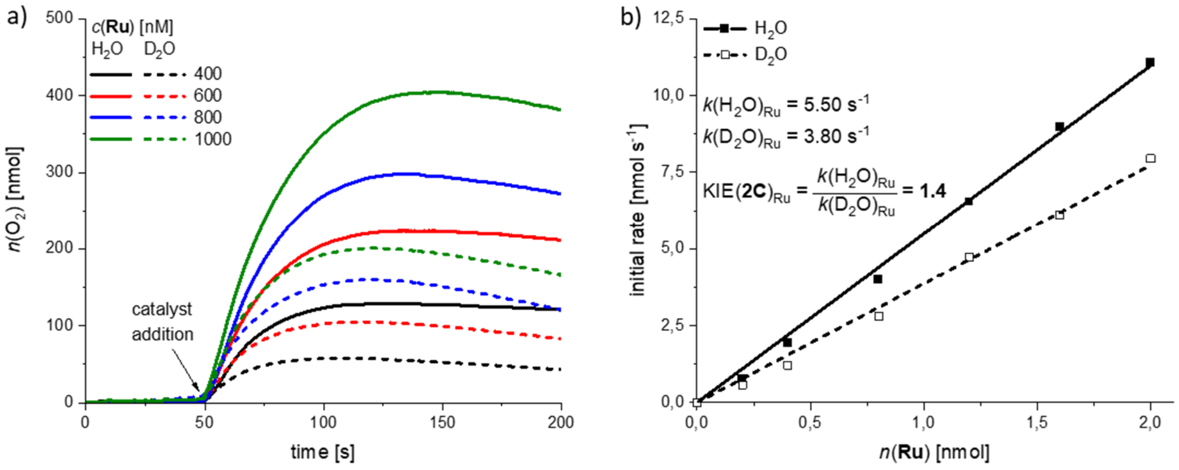

Figure S43. a) Concentration-dependent experiments for **2C per Ru unit** as WOC in CH_3CN/H_2O or D_2O 4:6 (pH 7, 50 mM phosphate buffer) *c*(PS) = 1.5 mM, *c*($Na_2S_2O_8$) = 37 mM. b) plot of the initial rates *vs.* the catalyst amount with linear regression for the determination of the individual reaction rates $k(H_2O)_{Ru}$ and $k(D_2O)_{Ru}$.

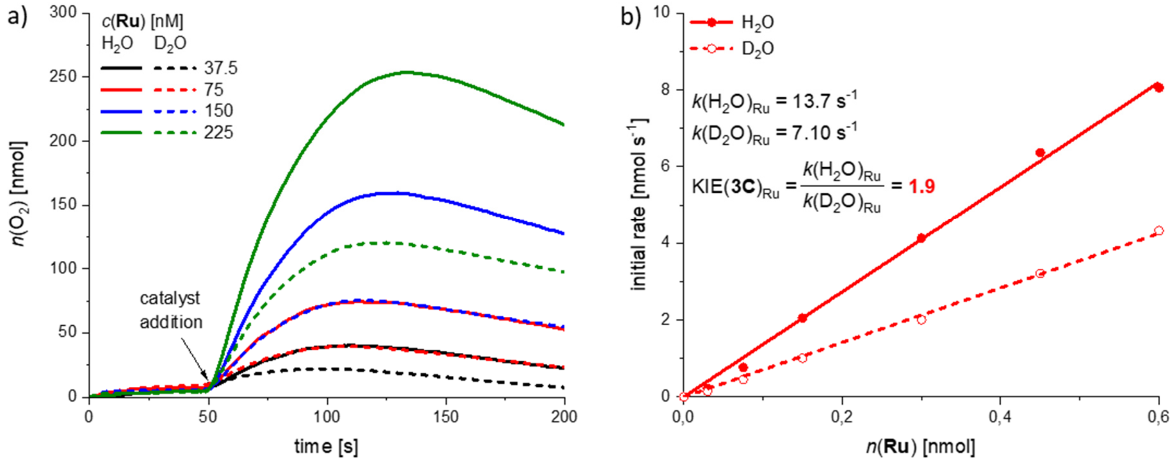

Figure S44. a) Concentration-dependent experiments for **3C per Ru unit** as WOC in CH_3CN/H_2O or D_2O 4:6 (pH 7, 50 mM phosphate buffer) *c*(PS) = 1.5 mM, *c*($Na_2S_2O_8$) = 37 mM. b) plot of the initial rates *vs.* the catalyst amount with linear regression for the determination of the individual reaction rates $k(H_2O)_{Ru}$ and $k(D_2O)_{Ru}$.

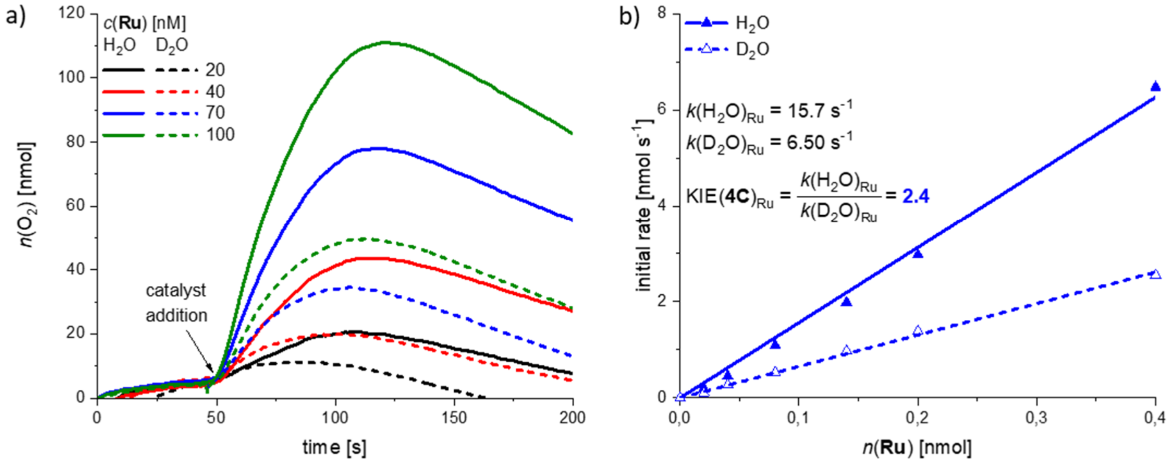

Figure S45. a) Concentration-dependent experiments for **4C per Ru unit** as WOC in CH_3CN/H_2O or D_2O 4:6 (pH 7, 50 mM phosphate buffer) *c*(PS) = 1.5 mM, *c*($Na_2S_2O_8$) = 37 mM. b) plot of the initial rates *vs.* the catalyst amount with linear regression for the determination of the individual reaction rates $k(H_2O)_{Ru}$ and $k(D_2O)_{Ru}$.

Electrochemistry

Table S7. Redox properties of the multinuclear complexes **2C**–**4C** under neutral (pH 7) aqueous conditions with 40% 2,2,2-trifluorethanol (TFE) as organic co-solvent. The measurements were performed in 0.1 M ionic strength phosphate buffered aqueous mixtures at the respective pH.[a]

Conditions	4:6 MeCN/H_2O (**pH 7**)		
Catalyst	*E vs.* NHE [V]		
	Ru^{III}/Ru^{II}	Ru^{V}/Ru^{IV}	Ru^{V}/Ru^{IV}
dimer **2C**	+0.67	+0.86	+1.04
trimer **3C**	+0.68	+0.84	+1.01
tetramer **4C**	+0.68	+0.83	+1.00

[a] CV and DPV measurements neutral conditions were performed at $c = 2.5 \cdot 10^{-4}$ M.

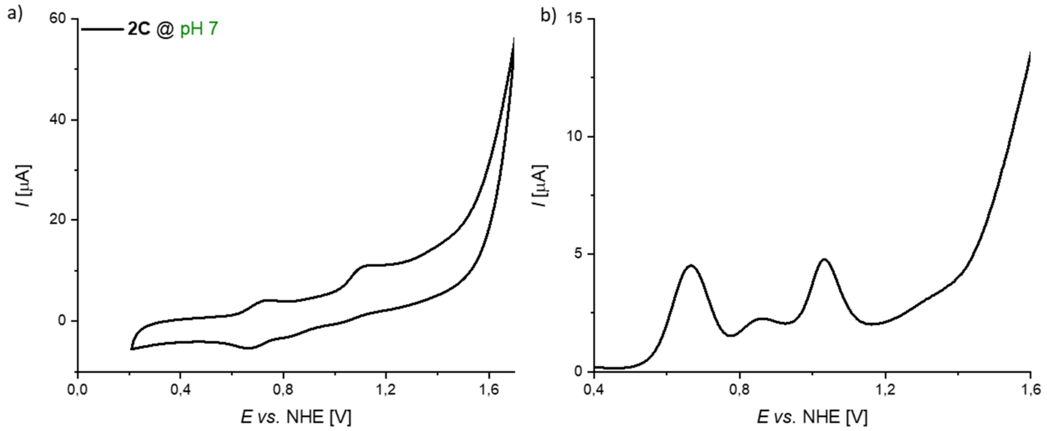

Figure S46. a) CV and b) DPV of **2C** in TFE/H_2O 4:6 (pH 7, phosphate buffer, $c = 2.5 \cdot 10^{-4}$ M).

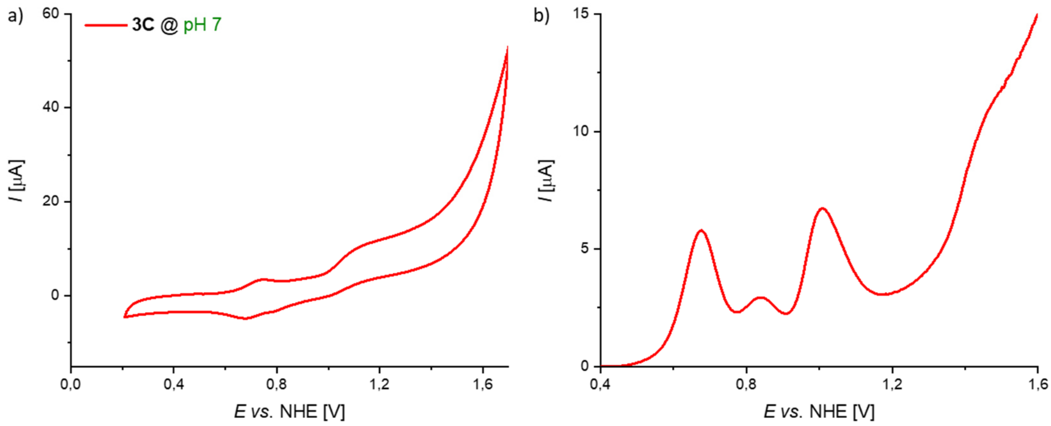

Figure S47. a) CV and b) DPV of **3C** in TFE/H_2O 4:6 (pH 7, phosphate buffer, $c = 2.5 \cdot 10^{-4}$ M).

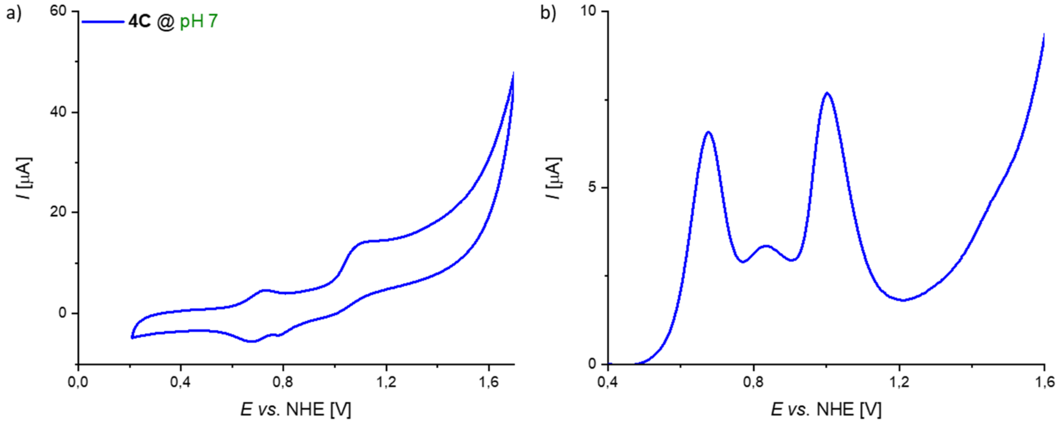

Figure S31. a) CV and b) DPV of **4C** in TFE/H_2O 4:6 (pH 7, phosphate buffer, c = 2.5·10^{-4} M).

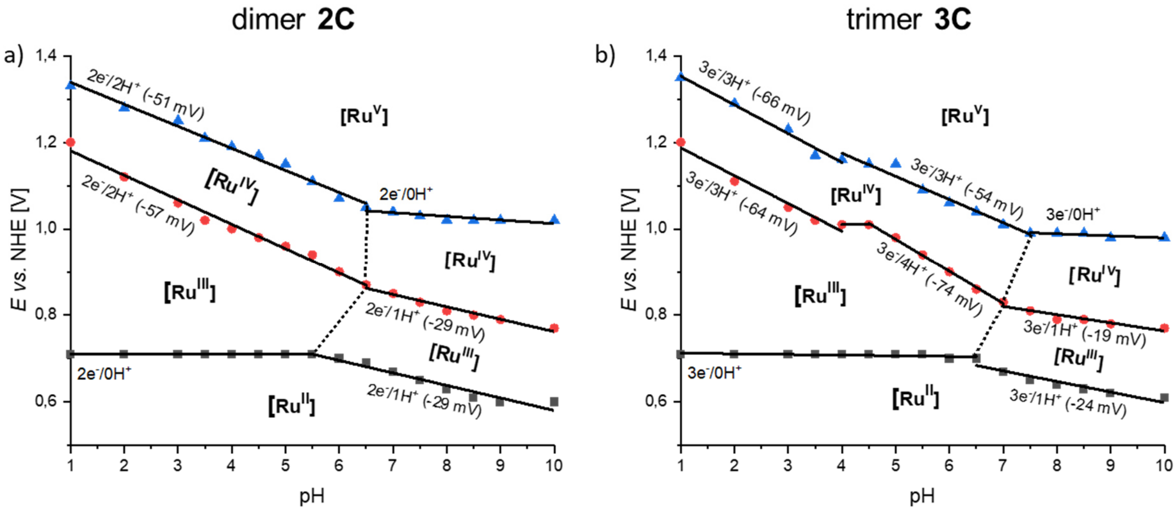

Figure S49. Pourbaix diagrams of a) dimer **2C** and b) trimer **3C**. Respective differential pulse voltammogram measurements were performed in TFE/H2O 4:6 (phosphate buffer at different pH values with I = 0.1 M, c(**WOC**) = 2.5·10^{-4} M).

NMR spectra

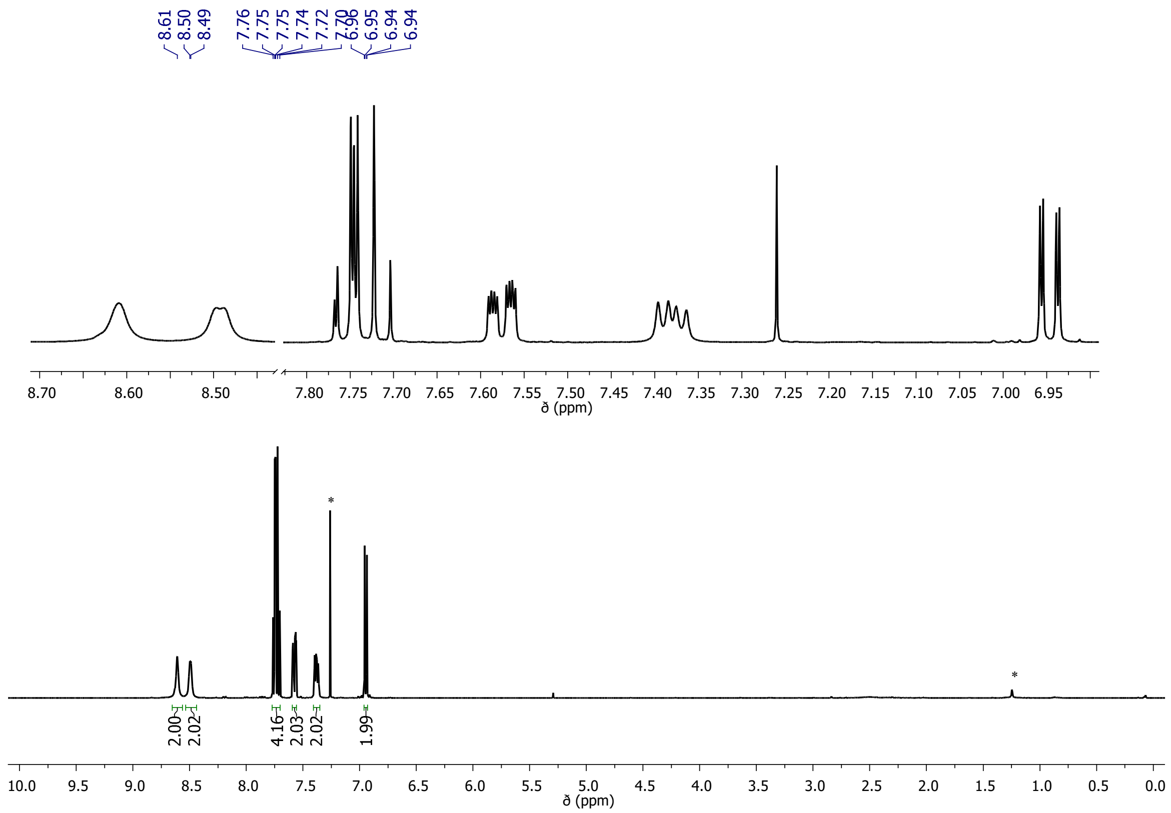

Figure S50. ^{1}H NMR spectrum (400 MHz, $CDCl_3$) of ligand **3** (* residual solvent).

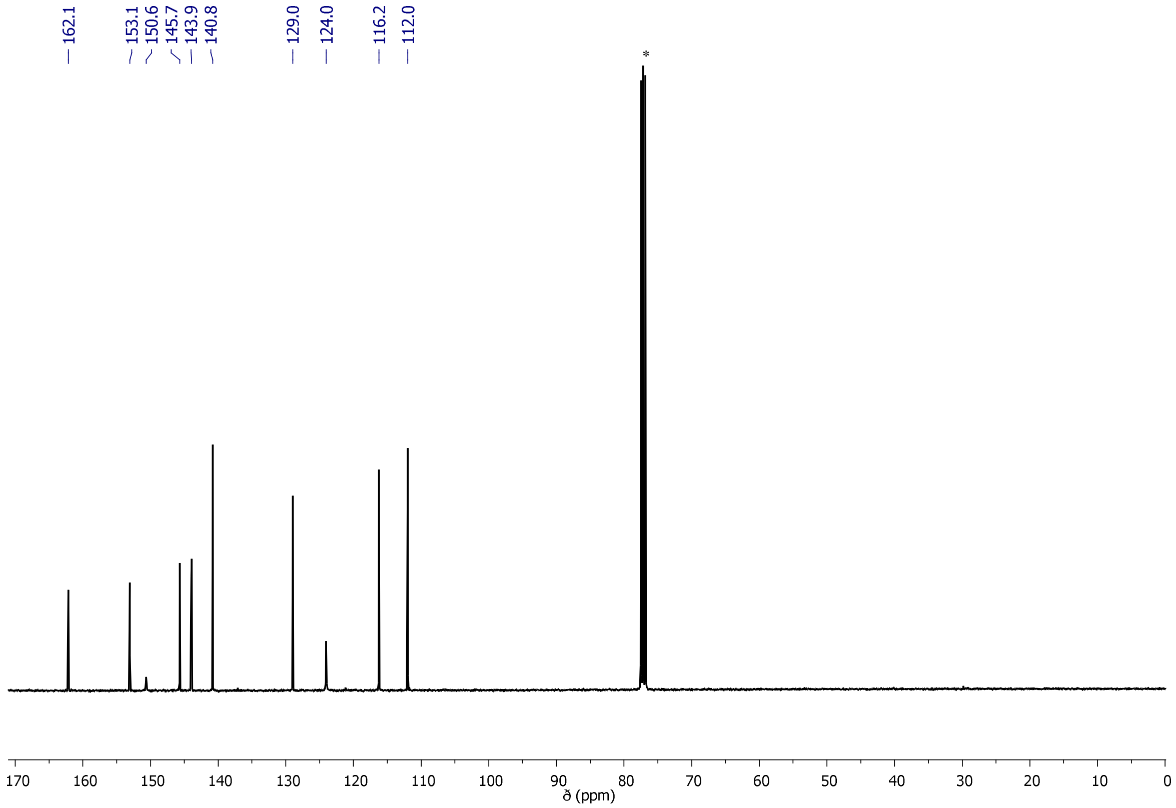

Figure S51. ^{13}C NMR spectrum (100 MHz, $CDCl_3$) of ligand **3** (* residual solvent).

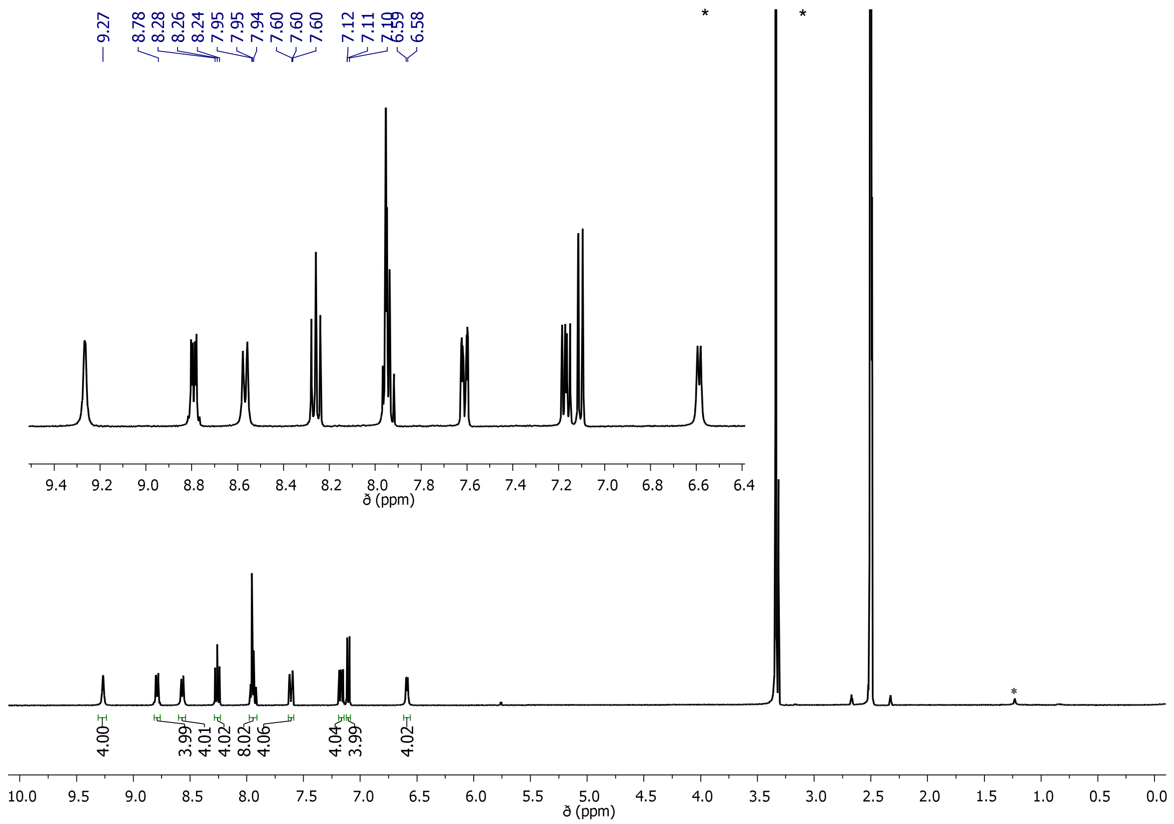

Figure S52. ^{1}H NMR spectrum (400 MHz, DMSO−d_6) of dimer **2C** (* residual solvent).

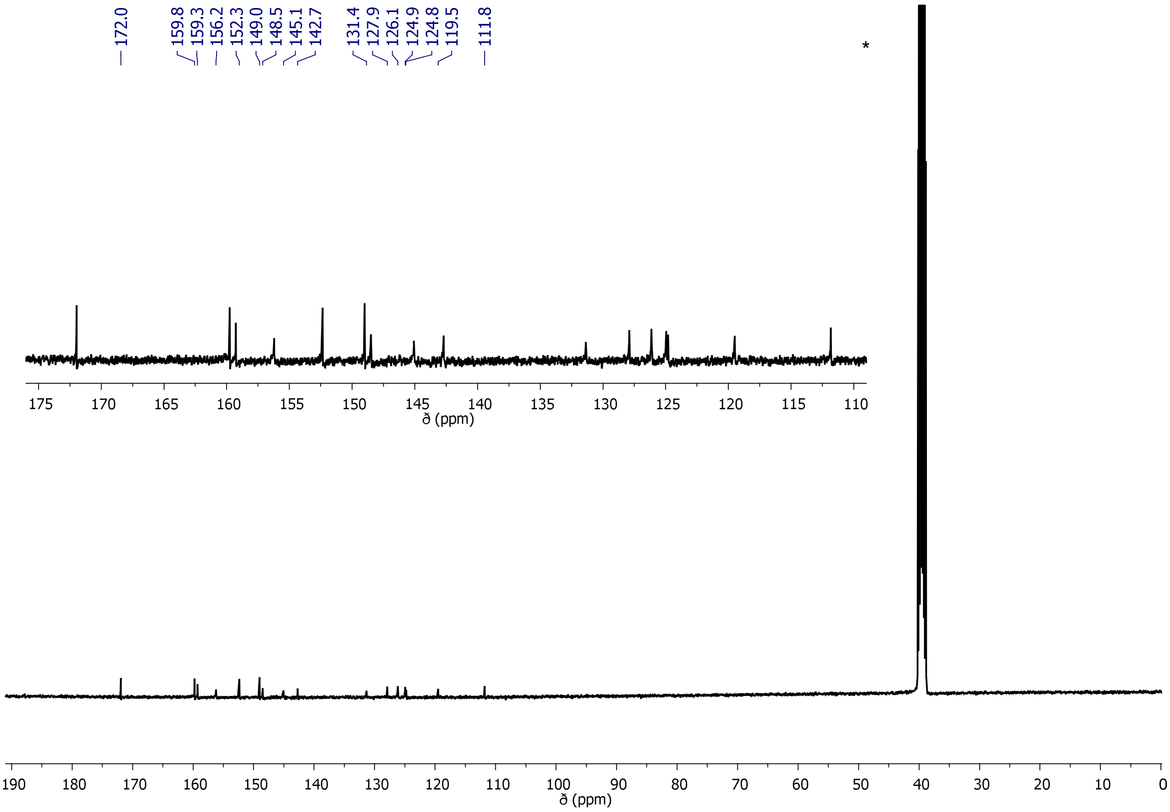

Figure S53. ^{13}C NMR spectrum (100 MHz, DMSO−d_6) of dimer **2C** (* residual solvent).

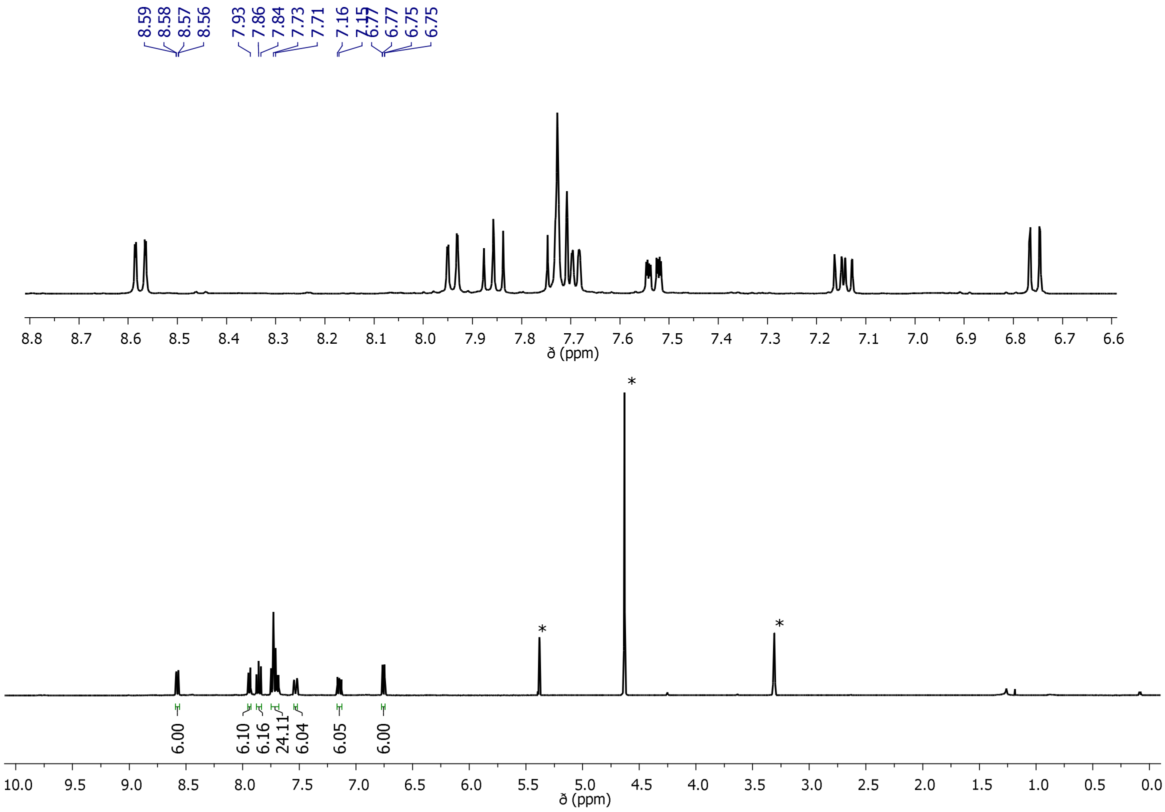

Figure S54. ^{1}H NMR spectrum (400 MHz, CD_2Cl_2/CD_3OD (1:1)) of trimer **3C** (* residual solvent).

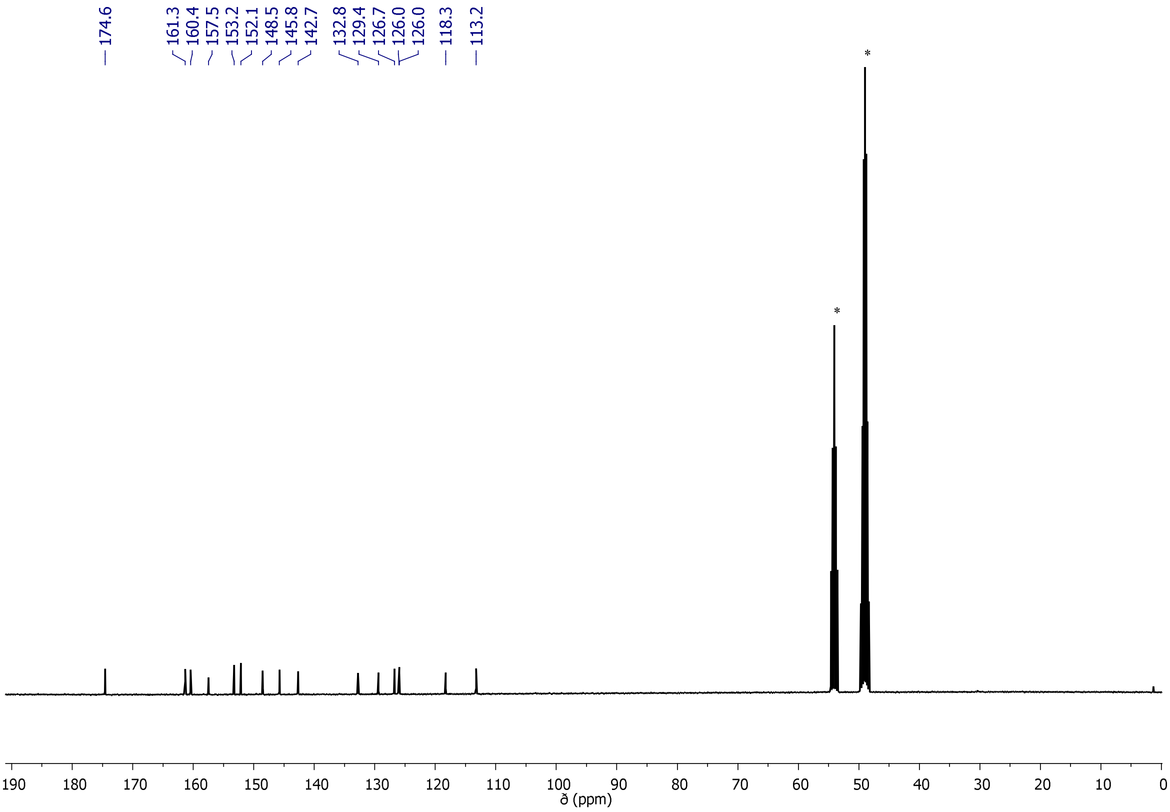

Figure S55. ^{13}C NMR spectrum (100 MHz, CD_2Cl_2/CD_3OD (1:1)) of trimer **3C** (* residual solvent).

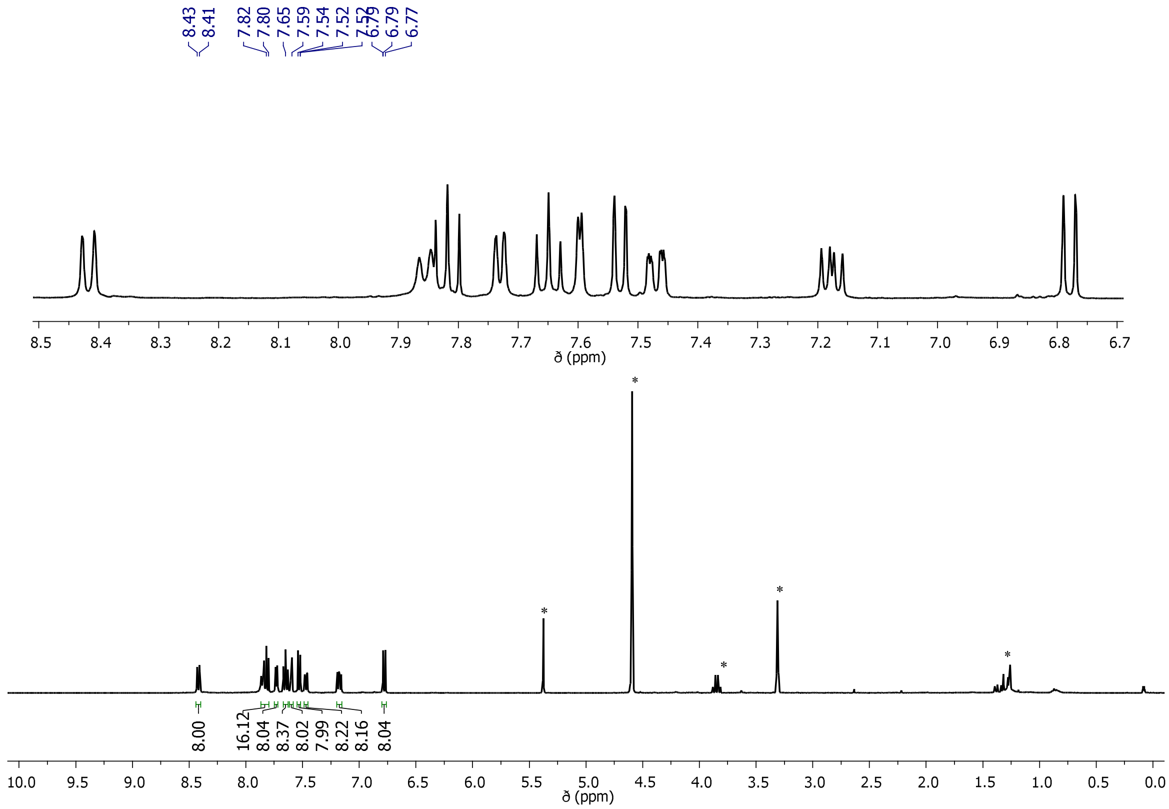

Figure S56. ^{1}H NMR spectrum (400 MHz, CD_2Cl_2/CD_3OD (1:1)) of tetramer **4C** (* residual solvent).

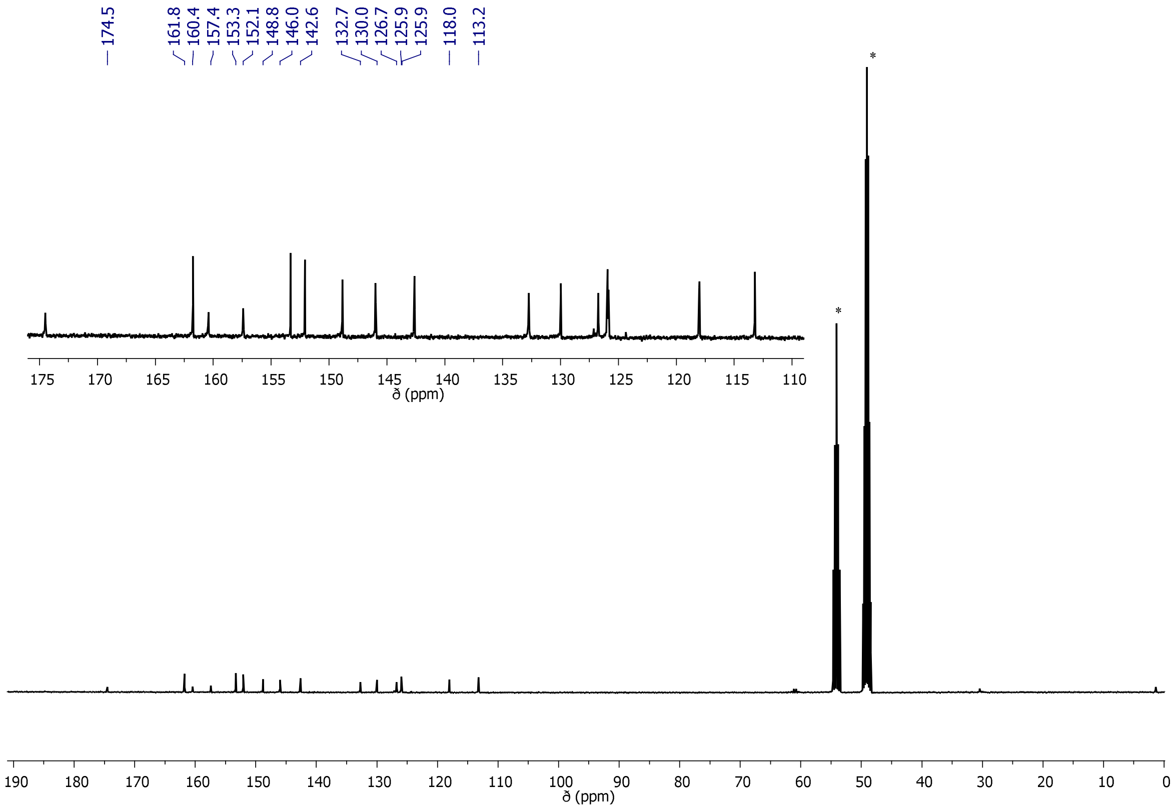

Figure S57. ^{13}C NMR spectrum (100 MHz, CD_2Cl_2/CD_3OD (1:1)) of tetramer **4C** (* residual solvent).

HR mass spectra

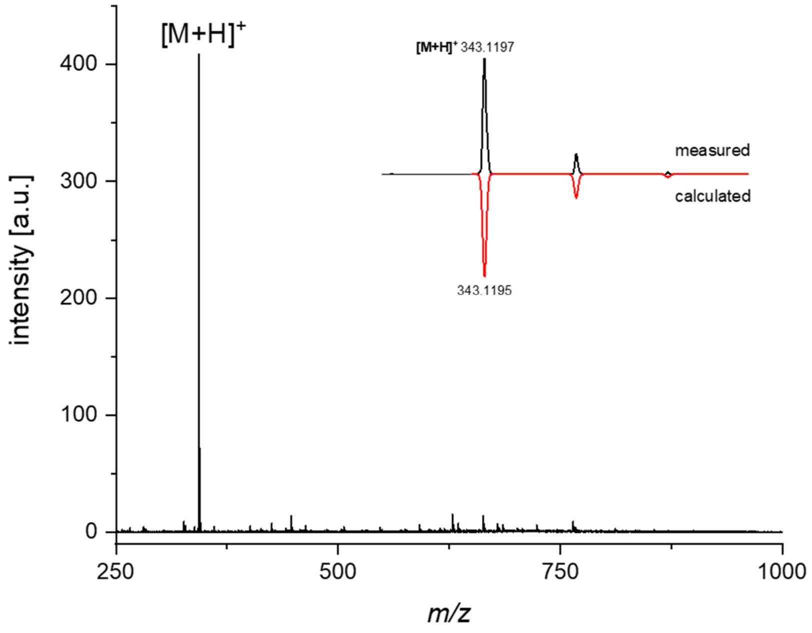

Figure S58. HRMS (ESI-TOF, pos mode) mass spectrum ($CH_3CN/CHCl_3$ 1:1, positive mode) of ligand **3** with the inset showing the measured and calculated isotopic distribution of $[M+H]^+$.

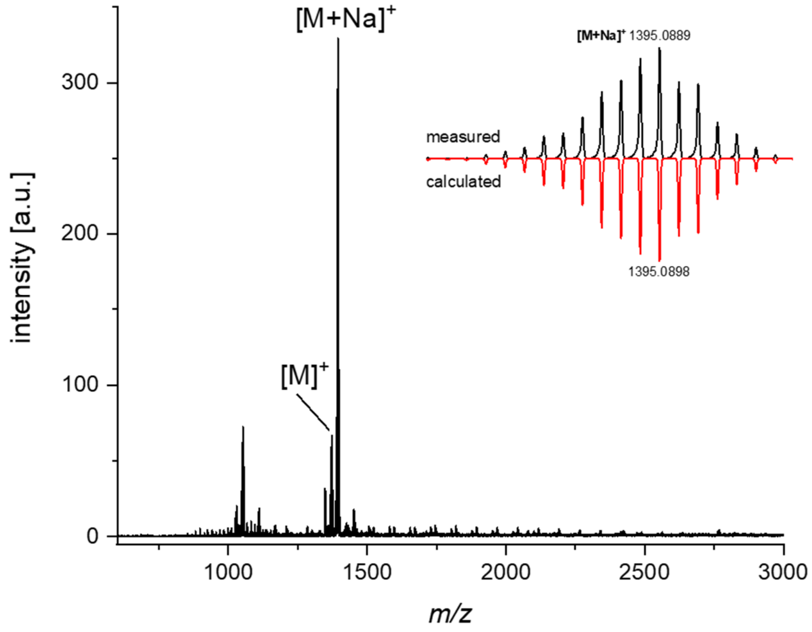

Figure S59. HRMS (ESI-TOF, pos mode) mass spectrum ($CH_3CN/CHCl_3$ 1:1, positive mode) of dimer **2C** with the inset showing the measured and calculated isotopic distribution of $[M+Na]^+$.

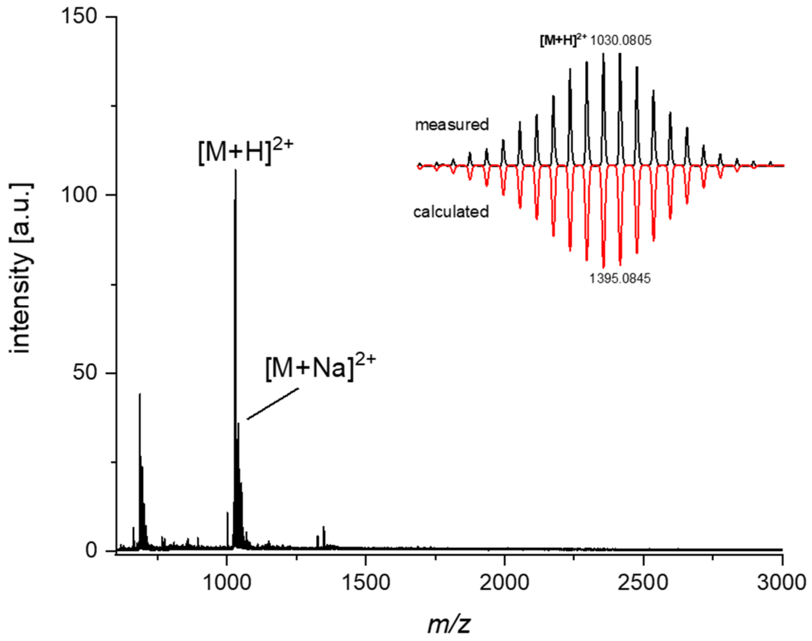

Figure S60. HRMS (ESI-TOF, pos mode) mass spectrum (CH_3OH/CH_2Cl_2 1:1, positive mode) of trimer **3C** with the inset showing the measured and calculated isotopic distribution of $[M+H]^{2+}$.

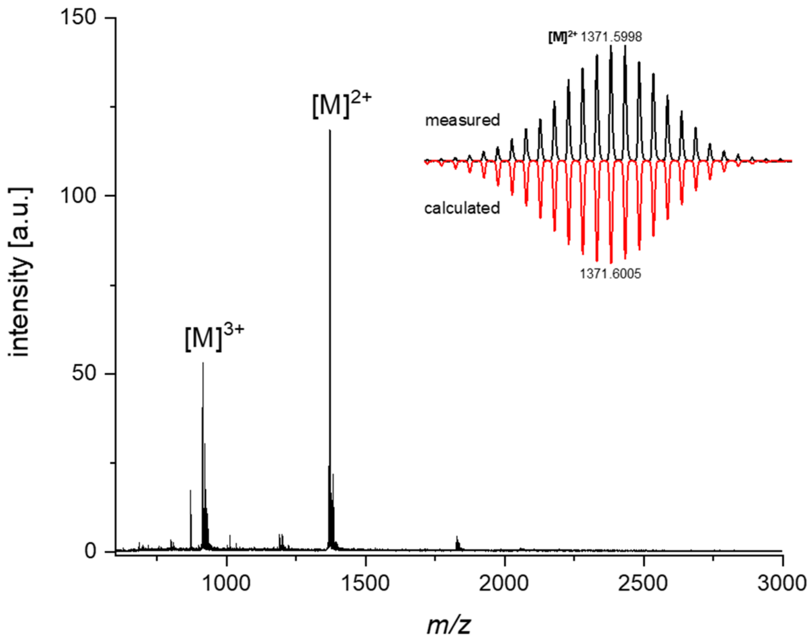

Figure S61. HRMS (ESI-TOF, pos mode) mass spectrum (CH_3OH/CH_2Cl_2 1:1, positive mode) of tetramer **4C** with the inset showing the measured and calculated isotopic distribution of $[M]^{2+}$.

References

[S1] C. L. Donnici, D. H. Maximo Filho, L. L. C. Moreira, G. Teixeira dos Reis, E. Cordeiro, I. M. Ferreira de Oliveira, S. Carvalho, E. B. Paniago, *J. Braz. Chem. Soc.* **1998**, *9*, 455–460.

[S2] I. P. Evans, A. Spencer, G. Wilkinson, *J. Chem. Soc. Dalton Trans.* **1973**, 204–209.

[S3] E. Dulière, M. Devillers, J. Marchand-Brynaert, *Organometallics* **2003**, *22*, 804–811.

[S4] F. Li, B. Zhang, X. Li, Y. Jiang, L. Chen, Y. Li, L. Sun, *Angew. Chem. Int. Ed.* **2011**, *50*, 12276–12279; *Angew. Chem.* **2011**, *123*, 12484–12487.

[S5] Y. Gao, X. Ding, J. Liu, L. Wang, Z. Lu, L. Li, L. Sun, *J. Am. Chem. Soc.* **2013**, *135*, 4219–4222.

[S6] G. R. Fulmer, A. J. M. Miller, N. H. Sherden, H. E. Gottlieb, A. Nudelman, B. M. Stoltz, J. E. Bercaw, K. I. Goldberg, *Organometallics* **2010**, *29*, 2176–2179.

[S7] F. Olivé, S. K. Chaudhari, K. R. Patil, A. Coronas, *Can. J. Chem. Eng.* **1996**, *74*, 163-169.

[S8] S. Gawęda, G. Stochel, K. Szaciłowski, *J. Phys. Chem. C* **2008**, *112*, 19131–19141.

[S9] W. Kabsch, *Acta Crystallogr. D* **2010**, *66*, 125–132.

[S10] Bruker, **2014**, XPREP Version 2014/2, Bruker AXS Inc., Madison.

[S11] G. M. Sheldrick, *Acta Crystallogr. A* **2015**, *71*, 3–8.

[S12] G. M. Sheldrick, *Acta Crystallogr. C* **2015**, *71*, 3–8.

[S13] A. L. Spek, *Acta Crystallogr. C* **2015**, *71*, 9–18.

[S14] A. L. Spek, *J Appl. Crystallogr.* **2003**, *36*, 7–13.

[S15] M. Schulze, V. Kunz, P. D. Frischmann, F. Würthner, *Nat. Chem.* **2016**, *8*, 576–583.

[S16] V. Kunz, J. O. Lindner, M. Schulze, M. I. S. Röhr, D. Schmidt, R. Mitrić, F. Würthner, *Energy Environ. Sci.* **2017**, *10*, 2137–2153.

[S17] A.-L. Meza-Chincha, J. O. Lindner, D. Schindler, D. Schmidt, A.-M. Krause, M. I. S. Röhr, R. Mitrić, F. Würthner, *Chem. Sci.* **2020**, *11*, 7654–7664.

[S18] N. Noll, F. Würthner, *Chem. Eur. J.* **2021**, *27*, 444–450.

[S19] The PyMOL Molecular Graphics Systeme, Version 2.4.2 Schrödinger, LLC.

[S20] L. Wang, L. Duan, B. Stewart, M. Pu, J. Liu, T. Privalov, L. Sun, *J. Am. Chem. Soc.* **2012**, *134*, 18868–18880.

[S21] L. Duan, Y. Xu, P. Zhang, M. Wang, L. Sun, *Inorg. Chem.* **2010**, *49*, 209–215.

[S22] L. Duan, F. Bozoglian, S. Mandal, B. Stewart, T. Privalov, A. Llobet, L. Sun, *Nat. Chem.* **2012**, 4, 418–423.

[S23] L. Wang, D. W. Shaffer, G. F. Manbeck, D. E. Polyansky, J. J. Concepcion, *ACS Catal.* **2020**, *10*, 580–585.

[S24] L. Francàs, R. Matheu, E. Pastor, A. Reynal, S. Berardi, X. Sala, A. Llobet and J. R. Durrant, *ACS Catal.*, **2017**, *7*, 5142–5150.

[S25] Y. Jiang, F. Li, B. Zhang, X. Li, X. Wang, F. Huang, L. Sun, *Angew. Chem. Int. Ed.* **2013**, *52*, 3398–3401; *Angew. Chem.* **2013**, *125*, 3482–3485.

[S26] F. Li, C. Xu, X. Wang, Y. Wang, J. Du, L. Sun, *Chin. J. Catal.* **2018**, *39*, 446–452.

[S27] S. Neudeck, S. Maji, I. López, S. Meyer, F. Meyer, A. Llobet, *J. Am. Chem. Soc.* **2014**, *136*, 24–27.

[S28] S. Berardi, L. Francas, S. Neudeck, S. Maji, J. Benet-Buchholz, F. Meyer, A. Llobet, *ChemSusChem* **2015**, *8*, 3688–3696.

[S29] D. Schindler, A.-L. Meza-Chincha, M. Roth, F. Würthner, *Chem. Eur. J.* **2021**, *27*, 16938–16946.
